# Acknowledgement to Reviewers of *Sensors* in 2018

**DOI:** 10.3390/s19010212

**Published:** 2019-01-08

**Authors:** Sensors Editorial Office

**Affiliations:** MDPI, St. Alban-Anlage 66, 4052 Basel, Switzerland

Rigorous peer-review is the corner-stone of high-quality academic publishing. The editorial team greatly appreciates the reviewers who contributed their knowledge and expertise to the journal’s editorial process over the past 12 months. In 2018, a total of 4507 papers were published in the journal, with a median time to first decision of 21 days and a median time to publication of 43 days. The editors would like to express their sincere gratitude to the following reviewers for their cooperation and dedication in 2018:
Aanstoos, James V.Lin, PengAarabi, ArdalanLin, ShanAaron, DavidLin, Shih-ChunAazam, MohammadLin, Tang-huangAbadal, SergiLin, Tsung-HungAbadi, Mojtaba MansourLin, Tzu-EnAbaid, NicoleLin, WeiAbate, AdamLin, Wen-YenAbawajy, Jemal H.Lin, XiaoshanAbbas, SawaidLin, Yang-weiAbbasi, Qammer H.Lin, Yuan-PinAbbod, MaysamLin, Yu-ChenAbd-Alhameed, RaedLin, Yu-ChengAbdelaziz, MahmoudLin, Yuh-ChungAbdelkarim, OmarLin, ZhaoyangAbdel-Motaleb, Ibrahim M.Lin, ZhibinAbdolee, RezaLin, ZuantaoAbdul-Aziz, AliLinden, MariaAbe, TakashiLindner, LarsAbeysekera, Saman S.Lindquist, Diana M.Abonyi, JánosLindquist, NathanAbot, JandroLing, SteveAbouhossein, AlirezaLingua, Andrea MariaAbramovich, HaimLinhares, JoãoÁbrányi-Balogh, PéterLinty, NicolaAbu Alkheir, AlaLinul, EmanoilAbu-Mahfouz, IssamLinzon, YoavAbu-Siada, AhmedLiolios, Konstantinos A.Abuzneid, Abdel-ShakourLiopo, AntonAccardo, DomenicoLiou, MichelleAcerbi, FabioLipnickas, ArūnasAcevedo, Rubén CarlosLis, Jose Vicente RosAcha, VictorLisani, José LuisAcharya, Tri DevLisauskas, AlvydasAcharya, U RajendraLisenkov, IvanAchterberg, Eric P.Lissenden, Cliff J.Ackleson, SteveLissenden, Clifford J.Acquah, Gifty E.Lissorgues, GaelleAdachi, YoshiakiLitvinova, LarisaAdán, AntonioLitwin, DariuszAdányi, NóraLiu, Alan Z.Adão, TelmoLiu, AnfengAddabbo, PiaLiu, BoAdegoke, OluwasesanLiu, ChangAdekanmbi, EzekielLiu, ChangqingAdelantado, FerranLiu, ChaoAdiletta, GiuseppinaLiu, Chien-HaoAdinoyi, AbdulkareemLiu, Chien-HungAdjrad, MounirLiu, Chien-ShengAfanasyev, IlyaLiu, ChuanjunAfsharipour, BabakLiu, Chung-ChiunAfzal, AdeelLiu, ChunleiAgafonov, VadimLiu, DongxiAgah, ArvinLiu, FayaoAgapiou, AthosLiu, FeiAgarwal, KushLiu, GuigenAgarwal, PriyanshuLiu, HonghaiAgarwala, ShwetaLiu, HongyunAgathoklis, PanajotisLiu, HongzhongAggelis, Dimitrios G.Liu, HuapingAghababaee, HosseinLiu, Hung-HuanAghajani, HalehLiu, Jen-ChiehAghakhani, AmirrezaLiu, JiAgili, Sedig S.Liu, JiangAglyamov, SalavatLiu, JiechaoAgocs, EmilLiu, JieminAgostini, MatteoLiu, JingAgranov, GennadiyLiu, JingfeiAgrawal, AnkitLiu, JingweiAguasvivas, SarahLiu, JinyunAguilar, LeocundoLiu, JunAguilar, Sergio RíosLiu, LiAguilar-Caballos, María PazLiu, LinfengAguilera, TeodoroLiu, LukaiAguirre-Salado, Carlos A.Liu, MengyuanAguirre-Soto, AlanLiu, MingAhi, KiarashLiu, QiAhlfors, SeppoLiu, QingAhmad, FauziaLiu, QiongAhmad, Muhammad WaseemLiu, RanAhmadi, KavehLiu, ShenghengAhmadinia, AliLiu, Shing-HongAhmed, Afaz UddinLiu, SuxingAhmed, FaridLiu, TaihongAhmed, KareemLiu, TaoAhmed, MohiuddinLiu, TianyuAhmed, QadeerLiu, Tzong-shiAhmed, RajibLiu, WanchunAhmed, SnoberLiu, WanliAhmed, Syed HassanLiu, WeiAhmed, ZeeshanLiu, Wei-ChengAhmet Enis, CetinLiu, Wei-MinAhmetovic, DraganLiu, WeitingAhn, Chae HyuckLiu, WeiweiAhn, Choon KiLiu, WenAhn, Ji-YoungLiu, Wen-FungAi, ZhumingLiu, XianglinAiazzi, BrunoLiu, XiaoguangAicardi, IreneLiu, XiaokangAiguo, SongLiu, XiaoxiAinsworth, Thomas L.Liu, XinAissaoui, NesrineLiu, XunchenAjadi, Olaniyi A.Liu, YangAkbar, Sheikh A.Liu, YeAkbari, HamedLiu, YejunAkhtar, ZahidLiu, YingAkitsu, TetsuyaLiu, YingjunAkram, Muhammad NadeemLiu, YingxiangAksanli, BarisLiu, YonggangAktan, A. EminLiu, YuAkturk, AkinLiu, YuHaoAla, GuidoLiu, YukangAlam, FakhrulLiu, YunAlam, Md. MehboobLiu, ZhenglinAlam, Muhammad MahtabLiu, ZhenyuAlam, MushfiqulLiu, ZhiAlamdari, Mehrisadat MakkiLivens, StefanAl-Ameri, TalibLlamas Bello, CésarAl-Amri, MohammadLlaria, AlvaroAlander, JarmoLlobet, EduardAlani, OmarLo Brutto, MauroAlapan, YunusLo, Cheng-yaoAlappattu, Denny P.Lo, Kin HingAlbano, MicheleLo, Kuang YaoAlbarracín, RicardoLo, Nai-WeiAlbella, PabloLobo-Castañón, María JesúsAlbéri, MatteoLobov, SergeyAlbert, JacquesLockhart, ThurmanAlberti, Antonio MarcosLodi Rizzini, DarioAlberti, GiancarlaLogothetis, FotiosAlberti, GiovanniLogozzo, SilviaAlberto, GrecoLogsdon, SallyAlberto, NéliaLohan, Elena SimonaAlberto, Perea MorenoLohr, ChristopheAlbizu, IgorLoiola, Murilo BellezoniAlbukhanajer, WissamLomas-Vega, RafaelAlbuquerque, Daniel FilipeLombardi, FedericoAlbuquerque, RobsonLondono, BerlinAlbusac, JavierLong, Nicholas J.Alcaíno, PabloLong, XiAlcalá-Fdéz, JesúsLongo, DomenicoAlcarria, RamonLongstaff, Andrew PeterAlcock, LisaLonini, LucaAl-Dahidi, SameerLopatka, MarianAldrigo, MartinoŁopato, PrzemysławAleixandre Herrero, ManuelLopes, Sérgio I.Aleixandre, ManuelLópez De Lacalle, Luis N.Alejo, DavidLópez Espí, Pablo LuisAleksandrova, MariyaLópez Ruiz, NuriaAlencastre-Miranda, MoisesLopez Santos, OswaldoAlessi, AntoninoLópez, Antonio M.Aletta, FrancescoLópez, CristinaAlexandridis, ThomasLópez, Miguel ÁngelAlexandropoulos, GeorgeLópez-Alonso, José ManuelAlexandropoulos, George C.Lopez-De-Teruel, Pedro E.Alfa, AttahiruLópez-Granado, OtonielAlfano, BrigidaLopez-Hernandez, Francisco JoseAli, Md. AzaharLópez-Huerta, FranciscoAli, G. G. Md. NawazLopez-Iturri, PeioAli, HassanLópez-Lorente, Ángela InmaculadaAli, MdLópez-Mellado, ErnestoAli, MonsurLopez-Molina, CarlosAli, RaianLópez-Nava, Irvin HusseinAli-Löytty, SimoLopez-Otero, PaulaAlimenti, FedericoLópez-Salcedo, José AAlin, MoldoveanuLopomo, Nicola FrancescoAlirezaie, MarjanLoprencipe, GiuseppeAl-Isawi, Rawaa H KLoranger, SébastienAlizadeh, MojtabaLorenz, EckehardAljankawey, Abdualah S.Lorenzo Abad, María EncarnaciónAljaroudi, AliredaLorenzoni, LauraAl-Jumeily, DhiyaLorussi, FedericoAl-Khasawneh, AhmadLosada-Perez, PatriciaAlkhatib, HamzaLoseto, GiuseppeAllard, BrunoLoskot, PavelAllen, GrantLou, TiejiongAllende-Alba, GerardoLou, XinhuiAllwood, GaryLouro, PaulaAlmeida, AitorLovas, TamásAlmenárez, FlorinaLovchik, RobertAlmomani, AbdallahLove, Jeffrey J.Al-Naffakh, NeamahLovell, DavidAlobaidi, WissamLovisolo, LisandroAlomari, AbdullahLowe, ShehanAlonso González, ItziarLowry, MarkAlonso, ÁlvaroLozano Rogado, JesúsAlonso, LuisLu, ChaoAloqaily, MoayadLu, Chia-FengAlparone, LucianoLu, Chih-WenAlsadik, BasharLu, Ching-TaAlsaleem, FadiLu, Chun-ShienAl-Sarawi, SaidLu, GeyuAlshaer, HamadaLu, huihuiAlsharoa, AhmadLu, HuiminAlsheikh, Mohammad AbuLu, JianAlsina-Pagès, Rosa MaLu, JiangAl-Tahir, RaidLu, KuangdaAltan, HakanLu, LunaAltini, MarcoLu, NingAlvarelhão, JoséLu, PanÁlvarez Franco, Fernando J.Lu, PingAlvarez Lopez, YuriLu, RongxingAlvarez, FedericoLu, SiliangÁlvarez, FernandoLu, Tzu ChuenAlvarez, Juan CarlosLu, WeidangAlvarez-Bermejo, Jose AntonioLu, XiyuanAlvarez-Garcia, Juan AntonioLu, YiAlvarez-Loza, PabloLu, YipengÁlvarez-Tamayo, Ricardo I.Lubal, PřemyslAlves, Ana Cristina OliveiraLubczonek, JacekAlves, Daniele Barroca MarraLucchi, ElenaAlves, José CarlosLuck, RudyAlves, TiagoLuckner, MarcinAl-Yaari, AmenŁuczak, SergiuszAmanowicz, MarekLudeno, GiovanniAmato, UmbertoLudwig, BerndAmatya, SurajLudwig, FrankAmbike, SatyajitLughofer, EdwinAmbrož, MihaLui, AntonnyAmbrozinski, LukaszLui, EricAmediek, AxelLuís, MiguelAmelard, RobertLukač, NikoAmendola, SaraLum, Guo ZhanAmezcua Correa, RodrigoLumentut, Mikail F.Amezquita-Sanchez, Juan PabloLuna Moreno, DonatoAmezquita-Semprun, KendrickLuna, CarlosAmigoni, SoniaLunner, ThomasAmin, M. JunaidLuo, ChaominAmiri, Amir MohammadLuo, FeiAmiri, Iraj SadeghLuo, Jia-NingAmiruzzaman, MdLuo, JunhaiAmmer, KurtLuo, MingAmory-Mazaudier, ChristineLuo, NingqiAmpatzidis, DimitriosLuo, ShanAmselem, GabrielLuo, TonyAmundarain, AiertLuo, XiwenAn, LuLuo, YanhuaAn, NanLuo, YiAn, QiLuo, YongAn, YunkyuLuo, ZhengtangAnagnostopoulos, MariosLuo, ZhiyuanAnand, VijayakumarLupan, OlegAnashkina, Elena A.Lupu, NicoletaAnastasova, SalzitsaLuqman, Muhammad MuzzamilAnastassiu, HristosLuque, AmaliaAnbarjafari (Shahab), GholamrezaLuque-Nieto, Miguel-AngelAncillao, AndreaLusk, AnneAnders, JensLusk, CraigAndersen, AndersLussier, BenjaminAndersen, Jens ChristianLuxey, CyrilAndersson, Gert ILv, ChenAndersson, KarlLvova, LarisaAndersson, Per OlaLyakhov, AndreyAndia, ManuelLyamin, NikitaAndoga, RudolfLysenko, Dmitry A.Andre, DanielLyutakov, OleksiyAndreescu, Gheorghe-DanielM. Garcia, NunoAndreev, SergeyM. M. Kahaki, SeyedAndreoni, GiuseppeMa, BingpengAndressen, KjetilMa, ChangzhengAndrew, Ngo Chun YongMa, Der-MingAndrew, Trisha L.Ma, HuiAndrey Andreevich, GolovanMa, JiaAndriukaitis, DariusMa, JiajuAndročec, DarkoMa, JianAneece, Itiya PMa, JianguoAng, Chee WeiMa, JiayiAngeles, JorgeMa, JinwenAngelidis, PantelisMa, MingAnggraini, Sri AyuMa, NingAnghel, AndreiMa, WeiAnghelache, GabrielMa, WenpingAngrisano, AntonioMa, XingAnishchenko, LesyaMa, Yu TakAnisi, MohammadMa, Yuan-RonAnnamdas, Venu Gopal MadhavMa, YuqiangAnnanouch, Fatima EzahraMa, ZhenliangAnnus, PaulMa, ZhouAntal, HibaMaas, Hans-GerdAntao, Dion S.Määttä, SaraAnthes, RichardMacedo, LuísAntolini, FrancescoMach, PavelAntona, MargheritaMachac, JanAntoni, JeromeMachacek, ZdenekAntoniadis, IoannisMachado, AlencarAntonopoulos, AngelosMachado, RenatoAntonopoulos, Christos (Spain)Machaj, JurajAntonopoulos, Christos (Freece)Macher, Janet M.Aoki, KazuhiroMacher, WolfgangAoki, YosukeMachida, KatsuyukiAoyagi, TakahiroMachida, ShinjiroAparicio, NievesMacias-Guarasa, JavierAparicio, SofíaMacias-Lopez, Elsa MaríaAparicio-Pardo, RamonMačiulienė, MonikaApelt, FedericoMacnae, James C.Apetrei, ConstantinMadeira, Rui NevesApollo, NicholasMadokoro, HirokazuAppiah, KofiMadrigal, Carlos A.Arafa, AhmedMadruga, Francisco J.Aragues, RosarioMadsen, Claus B.Arakawa, TakahiroMadurapperuma, BuddhikaAramesh, MortezaMaga, DusanArana, MiguelMagalhães, Luís GonzagaAranda, MiguelMagda, JulesArandjelović, OgnjenMagdalena, PalaczAranha, JoseMagdziak, MarekAraque Quijano, Javier LeonardoMagele, ChristianArastounia, MostafaMagierowski, SebastianArata, JumpeiMagna, GabrieleAraujo, AlvaroMagnan, PierreAraujo, FilipeMagne, SylvainAraujo, GustavoMahabir, RonAraujo, William Reis DeMahanti, AniketAravand, M. AliMaher, SimonArazuri, SilviaMahmood, AhmedArboleya, AnaMahshid, Sahar SadatArcia-Moret, AndrésMaidment, NigelArdid Ramírez, MiguelMaimaitiyiming, MatthewArdigo, LucaMaiolino, PerlaArdila-Rey, Jorge AlfredoMaiorana, EmanueleArdizzi, MartinaMaita, FrancescoArges, ChristopherMaiz, JonArghira, NicoletaMajerus, SteveArgüello, FranciscoMajewski, JacekArgyri, Anthoula AMajewski, MaciejAri, Ado Adamou AbbaMajhi, Sanjit ManoharAricò, PietroMaji, ParthaAriga, KatsuhikoMakantasis, KonstantinosAriño, CristinaMakarona, E.Aritsugi, MasayoshiMakarov, Valeri A.Ariza-Lopez, Francisco JavierMakhoul, AbdallahArjunan, Sridhar PoosapadiMaki, KosukeArmada, ManuelMakihara, YasushiArnesano, MarcoMakihata, MitsutoshiArnold, Francisco JoséMakino, YoshioAroca, RafaelMakonin, StephenArreaga-Salas, David E.Makrakis, DimitriosArribas, JavierMakris, ChristosArriola, AitorMaktoomi, Mohammad AArroyo Ohori, KenMakur, AnamitraArroyo, NetzahualcóyotlMalajner, MarkoArroyo-Mora, Juan PabloMalakar, NabinArrue, Begoña C.Malara, PietroArscott, SteveMalassiotis, SotirisArtese, GiuseppeMaldague, XavierArtiemjew, PiotrMalecha, KarolArvanitis, KonstantinosMalekian, RezaArvin, FarshadMalgosa-Sanahuja, JosemariaAryal, AchyutMalik, AdityaAryal, JagannathMalik, HassanAryan, PouriaMalik, Rizwan AhmedArzuaga, EmmanuelMalka, DrorAsadnia, MohsenMallardo, VincenzoAsami, ToshihikoMallick, MahendraAsanuma, IchioMalloggi, FlorentAscorbe, JoaquinMalmberg, PerAsensio Marco, CesarMalone, Brendan P.Asghari, HosseinMalumbres, Manuel PérezAsher, WilliamMalvezzi, MonicaAshjaei, MohammadMalvoni, MariaAshley, JonMalyuskin, OleksandrAshok, AshwinMammone, NadiaAsif, Muhammad TayyabMan, KalokAsikuzzaman, MdManakhov, AntonAsimakopoulos, GrigoriosMancini, SimoneAskarinejad, HosseinMandal, SoumyajitAsorey-Cacheda, RafaelMandanici, EmanueleAssunção, PedroManea, IonAstrain, José JavierManera, ValeriaAtalay, OzgurManfred, KatherineAtamuradov, VepaMangan, MichaelAthanasiou, LamprosMangina, EleniAthanasopoulos, NikolaosMani, Ganesh KumarAtia, MohamedMani, VeerappanAtkinson, GaryManich, SalvadorAttia, AhmedManikas, AthanassiosAttila, KertészManikas, Theodore W.Atukeren, ErdalManjakkal, LibuAu, Kwok Wai SamuelManjare, ManojAu, Sam H.Manjarrés, DianaAubry, AugustoManjunath, ManuboluAuguste, Jean-LouisMankey, GaryAugustine, RobinMann, SteveAugusto Costa Fernandes, MarceloManno-Kovacs, AndreaAugustyniak, PiotrManogaran, GunasekaranAureli, MatteoManojlović, Lazo M.Auriat, AngelaManso Callejo, Miguel ÁngelAuvinet, EdouardMansouri, AlaminAuxilia, MalarMante, Pierre-AdrienAvci, OnurMantzaris, Alexander V.Avdelidis, Nicolas P.Manzanares-Lopez, PilarAvgouropoulos, GeorgeManzano-Agugliaro, FranciscoAvila-Paredes, Hugo J.Manzoni, PietroAvramescu, ViorelMao, LeiAvtar, RamMao, Zhi-HongAwad, Ali IsmailMaran, ViniciusAwatsuji, YasuhiroMarapareddy, RamakalavathiAyala, Helon Vicente HultmannMarasso, Simone LuigiAyers, Paul D.Marcato, JoséAzar, MassoodMarcelli, RomoloAzarbayejani, MohammadMarchetti, MarioAzim, RezaulMarchi, GabrieleAziz, MustafaMarchiano, RégisAzorín-López, JorgeMarciniak, TomaszAβmann, MarcMarcos, MarcosBáča, TomášMarcos, MargaBachler, MartinMardanbegi, DiakoBacigalupo, AndreaMarek, LukasBackhouse, ChrisMarfievici, RamonaBa̧czyk, Marcin KamilMargheri, AndreaBadea Doni, MihaelaMaria, CorosBadger, MereteMariani, StefanoBadihi, HamedMarin, AndreaBadura, PawełMarinakis, VangelisBae, EuiwonMarinello, FrancescoBae, HyungdaeMarinesco, StéphaneBae, Ihn-hanMarinescu, AndreiBae, Kwang-HoMarinić-Kragić, IvoBae, Sung-HoMariotto, IsabellaBae, YoungchulMarjovi, AliBaek, DonghyunMarkakis, Evangelos K.Baek, Kwang RyulMarken, FrankBaeza, Francisco JavierMarkevicius, VytautasBaeza, MireiaMarkiewicz-Kȩszycka, MariaBaez-Nieto, DavidMarkopoulos, Angelos P.Baghaie, AhmadrezaMarkowski, KonradBaghdasaryan, TigranMarks, MichalBagnaninchi, PierreMarks, Robert S.Bahrami, ShahabMarmelat, VivienBai, Chun-xueMarmugi, LucaBai, LuMarozas, VaidotasBai, OuMarpu, Sreekar BabuBai, ShiqiangMarques, CarlosBai, YuMarques, Carlos Alberto FerreiraBailey, DonaldMarques, GonçaloBailly, Jean-StéphaneMarques, Manuel Joaquim BastosBaiocchi, ValerioMarques, Manuel JorgeBajer, JosefMarrazza, GiovannaBaker, TharMarrero, DomingoBakran, MarkMarsal, Lluis F.Bakuła, KrzysztofMarsh, StuartBakuła, MieczysławMarshall, JulianBala, MikloshMarshall, Mark T.Balador, AliMarszałek, ZbigniewBalaji, BharathanMarta, Ferreiro-GonzálezBalaji, BhashyamMartalò, MarcoBalakrishnan, DeepanMartin, AidaBalali, VahidMartin, SergioBalazadegan-Sarvrood, YasharMartincic, EmileBalázs, BoldizsárMartínez Torres, JavierBalázs, PéterMartínez Zaldívar, Francisco JoséBaldrich, EvaMartínez, AntoniBalduzzi, GiuseppeMartinez, DominiqueBalenović, IvanMartinez, Francisco J.Balestra, GabriellaMartinez, JoaquinBall, John E.Martínez, José-FernánBall, TonioMartinez, ManuelBallesta, MonicaMartinez, NicoleBallesta-Claver, JulioMartinez, Santiago GilBallesteros, PalomaMartinez-Agirre, AlexBalloy, VivianeMartínez-Aires, María DoloresBaloch, ZafarMartínez-Bisbal, M. CarmenBalsa-Barreiro, JoseMartinez-Carranza, JoséBalss, UlrichMartínez-García, EdgarBalzano, WalterMartinez-Hernandez, UrielBalzer, JanMartinez-Martin, EsterBanan Sadeghian, RaminMartínez-Martínez, VíctorBanavar, Mahesh K.Martinez-Millana, AntonioBandodkar, Amay J.Martínez-Mora, Juan A.Banerjee, PriyankaMartinez-Rios, AlejandroBang, HyochoongMartinez-Tarifa, Juan ManuelBang, OleMartinez-Triguero, JoaquinBañón, LuisMartínez-Villaseñor, Ma. LourdesBao, YiMartin-Gutierrez, JorgeBao, YinyinMartini, AlbertoBao, ZhenanMartin-Lopez, SoniaBaptista, Pedro VianaMartín-Mateos, PedroBaraldi, PieroMartino, LucaBaran, RemigiuszMartins Da Silva, MairaBaray, Jean-LucMartins De Mendonça, LaércioBarba, SalvatoreMartins, Felipe NascimentoBarbarella, MaurizioMartins, João P. A.Barbarin, YohanMartins, JorgeBarbato, MarcoMartins, Marcos SBarbedo, Jayme Garcia ArnalMartins, Wallace AlvesBarber, RamónMartín-Sacristán, DavidBarbera, JoaquinMartins-Filho, Luiz S.Barbillon, GrégoryMartín-Yerga, DanielBarbon Jr, SylvioMárton, LorincBarbone, PaulMartowicz, AdamBarbosa, Ramiro S.Maruda, Radosław W.Barbosa, RommelMarzo, TizianoBarbosa, Talles Marcelo G De AMarzocca, CristoforoBarbu, AdrianMas, DavidBarclay, DavidMasago, YoshifumiBardera, AntonMasala, CarlaBarfidokht, AbbasMasamune, SadaoBarile, ClaudiaMascarenas, DavidBarmatov, EvgenyMascarenhas, Nelson D.A.Barmpoutis, PanagiotisMašek, PavelBaron, RonanMasek, VlastimilBarone, Pier MatteoMashima, DaisukeBarra, SilvioMasiello, GuidoBarrado, CristinaMasiero, AndreaBarreca, FrancescoMasini, Barbara MaviBarreca, SalvatoreMasip, DavidBarrera, EduardoMaślikowski, ŁukaszBarrett, BrynleMasoero, MarcoBarriot, Jean-PierreMassad-Ivanir, NaamaBarros, Michael TaynnanMassaouti, MariaBarros, PabloMassaroni, CarloBarsan, Madalina M.Masse, AntoineBarsanti, Sara GonizziMastmeyer, AndreBarsi, ArpadMatano, FabioBarsocchi, PaoloMatatagui, DanielBartolomeu, PauloMateo, Carlos M.Barton, MichaelMateo, DavidBartoněk, DaliborMateos, CristianBartonova, AlenaMateos, JavierBartsch, HeikeMathias, Mauro HugoBartusek, KarelMathwig, KlausBartyś, MichałMatić, FranoBartzanas, ThomasMatikas, Theodore E.Bartzis, JohnMatikonda, SiddharthBaryannis, GeorgeMatinmikko-Blue, MarjaBaryla, RadoslawMatko, VojkoBaś, BogusławMatos, AníbalBas, JoanMatos, Joao NunoBasarab, Miklhail AlekseevichMatos, SergioBasaran, CemalMatsubara, TakashiBascetta, LucaMatsukura, HarukaBaselice, FabioMatsumura Araujo, RicardoBashkatov, Alexey NikolaevichMatsumura, KentaBašić, TomislavMatsuura, KazunoriBasiri, AnahidMatsuura, YujiBaskin, IgorMatsuyama, TatsushiBassler, MichaelMattar, CristianBassoli, RiccardoMatthews, James C.Bastakoti, Bishnu PrasadMatys, JacekBastidas, David M.Mauk, Michael G.Bastide, RémiMaune, HolgerBastos, Maria LuísaMauri, Jaime LloretBastos-Filho, Teodiano FreireMauri, RobertoBasu, AnirbanMayer, ChristopherBatard, ChristopheMayer, DirkBathrellos, GeorgeMayer, SimonBatule, Bhagwan SahebraoMaynard, Jonathan J.Bau Macià, JosepMayorov, AlexanderBaù, MarcoMaza, IvanBaudais, Jean-YvesMazaheri, MandanaBauer, PeterMažeika, DaliusBawaneh, KhaledMazeika, LiudasBazaei, AliMAZIZ, AliBazin, SaraMazurek, PrzemysławBažok, RenataMazzà, ClaudiaBazydło, GrzegorzMazzaro, GregoryBazzi, AlessandroMazzeo, GiovanniBeach, KevinMazzeo, Pier LuigiBeard, MichaelMazzetto, FabrizioBeauchamp, JonathanMcAlaney, JohnBęben, AndrzejMcCamley, JohnBecek, KazimierzMcCool, ChristopherBechelany, MikhaelMcFall, KevinBechhoefer, EricMcFarlane, NicoleBeck, JohnMcGilvray, KirkBecker, MarceloMcGinnis, RyanBecker, MatthiasMcheick, HamidBecker, RolfMcIntyre, Dustin L.Beckett, KeithMcKeague, MaureenBedekar, VishwasMcKeever, SusanBedogni, LucaMcLennan, KristaBedolla, DianaMcLeod, EuanBeerens, PeterMcllhagga, WilliamBeganovic, NejraMcLoughlin, Benjamin J.Begušić, DinkoMcnamara, ShamusBeheim, Glenn M.McNaughton, AnnaBehmann, JanMcPheron, Benjamin DBehunin, RyanMcWilliam, StewartBejinariu, Silviu-IoanMeaney, PaulBekas, Dimitrios G.Meara, PaulBekasiewicz, AdrianMedina Quero, JavierBekele, EsubeMedina, DanielBekiaris, GeorgiosMedjroubi, WidedBeko, MarkoMedola, FaustoBel, AlbertMedrano, CarlosBelagiannis, VasileiosMedrano, NicolásBelavič, DarkoMeek, Sanford G.Belay, KalayuMeekes, JoostBelehaki, AnnaMeese, Ernst A.Belfadel, DjedjigaMegías Jiménez, DavidBelfiore, Nicola PioMegret, PatriceBelgiu, MarianaMehan, SushantBeli, DaniloMeharouech, AmiraBell, Muyinatu A. LedijuMehdipour, ImanBellaiche, MartineMehic, MiralemBellekens, BenMehmood, RashidBellido, Francisco J.Mehta, SiddharthaBellvert, JoaquimMei, LeiBeltrame, GiovanniMei, Shao-HuiBeltran, ChrisMeijerink, AllardBelyaev, EvgenyMeikle, WilliamBen Zineb, TarakMeisen, TobiasBen, YueyangMeiss, AlbertoBenavidez, PatrickMejia-Beltran, EfrainBender, PaulMejuto, Juan CarlosBenedetto, AndreaMekhalfi, Mohamed LamineBenedicenti, LuigiMekikis, Prodromos-VasileiosBenetti, MassimilianoMelbye, HasseBenevides, PedroMelchionna, MicheleBenhabiles, HalimMelchor, JuanBeni, ValerioMelendez, Johan H.Benkhelifa, ElhadjMelendez-Pastor, IgnacioBennett, MichaelMelgar, DiegoBenni, StefanoMeli, EnricoBennis, MehdiMeli, LeonardoBenson, LaurenMelnik, EvaBentes, CarlosMelnykowycz, MarkBenz, HeatherMelo, JoséBenzerrouk, HamzaMeloni, AlessioBeranek, LadislavMelo-Pinto, PedroBerardi, Valentino PaoloMelvin, William L.Berbecaru, DianaMemedi, MevludinBerder, OlivierMemmolo, V.Berdjag, DenisMemoli, GianlucaBerenguel Soria, ManuelMenachery, AnoopBerényi, AntalMencarelli, FabioBereś-Pawlik, ElżbietaMendelson, YitzhakBerezin, Mikhail Y.Mendes, Ricardo UgliaraBergamasco, FilippoMendes, Virgilio BBergamini, ElenaMéndez, AlexisBerge, ThereseMendez, DiegoBergeot, NicolasMenegaldo, Luciano L.Bergmann, AlexanderMeneguette, RodolfoBergmann, NeilMeneses, FilipeBergmann, OlafMeng, FanbenBeriáin, AndoniMeng, WeiBerkovic, GarryMeng, WeizhiBerkvens, RafaelMeng, xiaolinBermudez Edo, MariaMeng, XuBernabe Garcia, SergioMeng, ZhaozongBernacki, BruceMeng, ZiboBernard, YvesMengarelli, AlessandroBernardini, RiccardoMenini, PhilippeBernardino, JorgeMeo, MariannaBernardo, LuisMeola, AntonioBernardos, AndreaMeola, CarosenaBernas, MarcinMera, DavidBernat, JakubMerazzo, Karla J.Bernier, MartinMercader, JosepBernini, RomeoMercatoris, BenoitBerruti, Teresa MariaMerchán, PilarBerthault, PatrickMercier, DavidBertocci, FrancescoMerciol, FrançoisBertoldo, SilvanoMercorelli, PaoloBertrand, SylvainMerenda, MassimoBertsch, ArnaudMerheb, JoeBertucci, AlessandroMeriggi, PaoloBerveglieri, AdilsonMerlen, AlainBesada, EvaMerletti, RobertoBesada, Juan A.Merlo, SabinaBesic, NikolaMerrison, Jonathan P.Bestak, RobertMersmann, FalkBétaille, DavidMesaros, AnnamariaBettini, PaoloMesas-Carrascosa, Francisco JavierBeutel, JanMeschino, SimoneBevacqua, MartinaMescia, LucianoBeverini, Nicolo’Meseguer, RocBevly, David M.Meshgi, KouroshBeyer, MichaelMesodiakaki, AgapiBeyerer, JurgenMesservey, ThomasBeyerer, JürgenMessina, FabrizioBeyreuther, ElkeMessina, MarcoBezodis, IanMessinger, MaxBhadra, SharmisthaMetcalfe, BenjaminBhakta, SnehasisMetelli, GiovanniBhalla, NikhilMetzner, MoritzBhandari, AyushMeza, PabloBhardwaj, AnshumanMeza-Ruiz, Iván VladimirBhattacharjee, TapomayukhMezei, IvanBhattacharya, DevanjanMezghani, FaroukBhattarai, KeshavMeziani, YahyaBhave, DevayaniMhaskar, PrashantBhosale, SheshanathMheen, BongkiBhuiyan, Md YeasinMiah, Abdul HalimBhuiyan, Mohammad Zahidul H.Miah, KhalidBhullar, Rajinder P.Miani, Rodrigo SanchesBi, HongshengMiao, JianminBi, ZhumingMiao, MengheBiagetti, GiorgioMiao, QiangBiagi, LudovicoMichael Ho, Siu ChunBiałoń, MariettaMichaelides, Michalis P.Bianchi, ValentinaMichaelsen, EckartBianco, VittorioMichalet, XavierBianconi, FrancescoMichalik, PiotrBianucci, PabloMichalos, GeorgeBibbo, DanieleMichard, FredericBica, IoanMichel, Ann-Katrin UrsulaBicen, A. OzanMicheli, LauraBie, RongfangMichie, CraigBien, AndrzejMichieletto, StefanoBieniaś, JarosławMichnik, RobertBiggs, TrentMicijevic, EsadBihl, Trevor J.Micucci, DanielaBikov, AndrásMieloszyk, MagdalenaBilleci, LuciaMihai, LauraBinelo, Manuel OsorioMihail, Radu PaulBinietoglou, IoannisMihara, MasahitoBiondi, FilippoMihaylova, LyudmilaBiondi, RiccardoMihelj, MatjažBiro, ElliotMihovska, AlbenaBischof, Hans-PeterMikhaylov, KonstantinBisello, AdrianoMikhelson, KonstantinBisio, IgorMiki, NorihisaBiswas, DwaipayanMiki, YasuhiroBiswas, SoumavaMikkelsen, KaareBithas, Petros S.Mikš, AntonínBittencourt, Luiz FernandoMilani, SimoneBjorklund, Robert B.Milanic, MatijaBjorninen, ToniMilanova, MariofannaBłachowicz, TomaszMilej, DanielBlachowski, BartlomiejMilella, AnnalisaBlais, AntoineMilic, VladimirBlakely, JonathanMilitino, Ana FernándezBlakely, Jonathan N.Miller, AlexanderBlanc, SaraMiller, BorisBlanco-del- Campo, ÁlvaroMiller, JimmieBlanco-López, María CarmenMiller, PaulBlanco-Rojas, DoloresMilowska, KarolinaBlasco, RubenMiluski, PiotrBlazevic, ZoranMimila-Arroyo, JaimeBleichner, MartinMin, Byung-WookBlekas, KonstantinosMin, MartBlendowski, MaxMinaee, ShervinBlesa Izquierdo, JoaquimMinakshi, ManickamBLIN, StéphaneMinamikawa, TakeoBlišťan, PeterMinamiki, TsukuruBlock, Jean-ClaudeMinardo, AldoBlock, StephanMinas, GraçaBlood, DanielMinav, TatianaBlumrosen, GaddiMinchew, BrentBlunt, PaulMinervini, MassimoBoada, Beatriz L.Minet, PascaleBoada, Maria Jesus LopezMing, ZuhengBobacka, JohanMingesz, RobertBobulski, JanuszMinghini, MarcoBochenkov, VladimirMinho, JoBochud, NicolasMiniaci, MarcoBock, ThomasMinussi, Carlos RobertoBocquet, MichaelMiranda, AntonioBodini, IleanaMiranda, JoaoBoehler, QuentinMiranda-Castro, RebecaBoes, AndreasMirás-Avalos, Jose ManuelBogena, HeyeMirbozorgi, AbdollahBohbot, JonathanMiribel-Catala, PereBohlin, AlexisMiridonov, SergueiBohorquez, JorgeMiron, SebastianBoland, JessicaMiroshnichenko, AndreyBoldt, MartinMirza, DibaBoletsis, CostasMirzavand, RashidBollella, PaoloMishne, DavidBolomey, Jean-CharlesMishra, Akhilesh KumarBolotin, Yu V.Mishra, AmitabhBolshov, Mikhail A.Mishra, Geetesh KumarBolten, AndreasMishra, Kumar VijayBolzmacher, ChristianMishra, Rupesh K.Bomberg, MarkMishra, SachidanandaBonache, JordiMiskowicz, MarekBonaci, TamaraMissell, FrankBonafoni, StefaniaMissell, Frank P.Bonaiuto, VincenzoMisurcova, LadislavaBonastre Pina, Alberto MiguelMitchell, CatherineBondorf, SteffenMitchell, JohnBonenberg, LukaszMitchell, PaulBongiorno, DanielMitchell, SteveBonham, Andrew J.Mitianoudis, NikolaosBonì, RobertaMitishita, EdsonBonin-Font, FranciscoMitobe, KazuhisaBontempi, NicolòMitra, SrinjoyBoquete, LucianoMiu, DanaBorboni, AlbertoMiwa, TomioBorecki, MichalMiyazaki, HiroyukiBorges Vieira, AlexMizouni, RabebBorghi, AlessandraMizsei, JánosBorghini, GianlucaMizuno, TomoyukiBorhani, AlirezaMizuno, YosukeBorio, DanieleMlejnek, PavelBorisov, SergeyMo, YuBorkowski, AndrzejMoafipoor, ShahramBorkowski, PiotrMobasheri, AminBorla, OscarMobashsher, Ahmed ToahaBormashenko, EdwardMocanu, Irina GeorgianaBorràs Sol, JúliaModares, JalilBorrelli, LuigiModenese, LucaBorri, SimoneModlitbova, PavlinaBoscaini, DavideModoni, GianfrancoBosch Ruiz, MarcMoerman, DavidBosch, StephanMoghadas, DavoodBoschi, FedericoMogo, SandraBose, DebojitMoh, MelodyBose, NeilMoh, SangmanBosisio, Ada VittoriaMohammad Karim, AlirezaBossé, ÉloiMohammadi, JavadBosser, PierreMohammed, AlyaaBossuyt, FrederikMohammed, Beadaa JasemBoswell, KevinMohan, Ganesh Babu MalliBotella, CarmenMohan, MidhunBotteron, CyrilMohd-Yasin, FaisalBouaziz, MoncefMohorcic, MihaelBoubchir, LarbiMois, GeorgeBoubeta-Puig, JuanMoiseev, SergeyBouchard, KevinMoisescu, Mihnea AlexandruBoucouvalas, Anthony C.Moldoveanu, FloricaBoudaden, JamilaMolin, Jose PauloBoudko, SvetlanaMolina Cantero, Alberto J.Boudon, FrédéricMolina Martinez, Jose MiguelBouk, SafdarMolinari, AndreaBoukhobza, JalilMolinier, MatthieuBoulogeorgos, Alexandros-Apostolos AMolinos Vicente, Eduardo JoseBourikas, LeonidasMöller, ReinhardBourillot, EricMolnar, MiklosBourqui, JeremieMolnar, VieroslavBousdar Ahmed, DinaMonaco, John V.Bousia, AlexandraMonasse, PascalBousquet, Jean FrançoisMondal, SudipBousquet, Jean-FrançoisMondal, Tapas KBoutaayamou, MohamedMonekosso, DorothyBoutayeb, HalimMongay Batalla, JordiBoutejdar, AhmedMongil-Manso, JorgeBoutet, AntoineMonica, RiccardoBouvet, Pierre-JeanMonico, Joao Francisco GaleraBouwmans, ThierryMoniri, MansourBouzefrane, SamiaMonno, YusukeBovenga, FabioMonowar, Muhammad MostafaBowen, JohnMontalvo, CarlosBowler, SimonMontanari, Gian CarloBoybay, Muhammed SaidMontano, LuisBoyer, MarcMontavont, NicolasBoyer, RemyMonte Lima, João Paulo Silva DoBozzano, RobertoMonteiro, Miguel PimentaBračun, DragoMontero, R. SánchezBrad, RemusMontes, MarcosBrad, StelianMontes-García, VerónicaBradac, ZdenekMontrucchio, BartolomeoBradley, SiobhanMontuori, AntonioBraem, BartMontuori, RosarioBraga, Guilherme S.Monzón-Hernández, DavidBragos, RamonMoo, PeterBrancato, VirginiaMoon, HyeonjoonBrandão, AlexandreMoon, JongsubBrandão, Lincoln CardosoMoon, Joo HyunBrandão, Michele A.Moon, Kee S.Brandl, MartinMoore, PhilipBrás, SusanaMoosmann, JulianBrassai, Sándor TihamérMora, AndreBraun, AlexanderMora, HiginioBravo Acha, MikelMorabito, Francesco CarloBravo, JoséMoradi, SaberBray, JoeyMorais, RaulBrčić, DavidMorais, SergiBrehar, RalucaMorales Luna, GuillermoBremer, KortMorales, Diego P.Brescia, MassimoMorales-Narváez, EdenBrethé, Jean FrançoisMoraru, LuminiţaBreus, TamaraMoreira, Cleumar Da SilvaBreuß, MichaelMorell, AntoniBrewer, GeorgeMorelli, AlessandroBrida, PeterMorelli, JordanBridwell, David AMoreno Catalá, MaríaBrincker, RuneMoreno-Salinas, DavidBrinkhoff, JamesMoreno-Valenzuela, JavierBriois, PascalMorgan, GrahamBrisolara, Lisane Brisolara DeMori, SaverioBrock, HeikeMorison, GordonBrockherde, WernerMoritz, Guilherme LuizBroderick, PatriciaMoriuchi-Kawakami, TakayoBrolly, MatthewMoriyama, ToshifumiBröring, ArneMoro, LorenzoBrosed, Francisco JavierMoron, CarlosBroumandan, AliMoroni, DavideBrouzgou, ΑngelikiMoroni, MonicaBrown, AdrianMorosi, SimoneBrown, John N.A.Morozs, NilsBrown, JustinMorris, TimBrown, Tim W. C.Morshed, BashirBrowning, AndrewMorsy, SalemBrščić, DraženMortari, DanieleBruckner, GudrunMortazavi, JackBruder, StephenMoscato, VincenzoBruggeman, PeterMoschitta, AntonioBruggemann, Troy SterlingMoschou, DespinaBrumen, BoštjanMoseke, ClausBrumfield, Brian E.Moshou, DimitriosBrun, XavierMostafaei, HabibBruna, ArcangeloMota, EdjairBrundage, Michael P.Mota, Rafael Perazzo BarbosaBrunelli, DavideMotalleb, MahdiBrunesi, EmanueleMotamedi, Gholam K.Brunete, AlbertoMotella, BeatriceBruniecki, KrzysztofMotta, GianmarioBruno, A. C.Motta, NunzioBruno, JohnMottet, DenisBruno, RaffaeleMouapi, AlexBruns, TimMouillot, FlorentBrunton, EmmaMoujahid, AbdelmalikBruschini, ClaudioMoulick, AmitavaBrusey, JamesMoura, JoseBrust, MatthiasMourato, SandraBrutti, AlessioMouri, KousukeBrzeski, PiotrMousas, ChristosBrzostowski, KrzysztofMousavi Lajimi, S. AmirBu, HonggangMousavi, S. MostafaBucciarelli, MartaMousavi, ZekraBuccini, AlessandroMoussallam, YvesBuch, Anders GlentMoutou Pitti, RostandBuchs, GillesMozaffari, MohammadBucinskas, VytautasMozos, IoanaBuckley, JohnM’sirdi, Nacer KouiderBudaszewski, DanielMu, XuanBudge, ScottMuaaz, MuhammadBudzan, SebastianMuanenda, YonasBugajski, MaciejMuceli, SilviaBui, NicolaMúčka, PeterBujnowski, AdamMudie, KurtBukin, OlegMuench, FalkBukkapatnam, Satish T.S.Muhamadali, HowbeerBukowska, JolantaMuhlethaler, PaulBulic, NevenMujica, LuisBull, GeoffMukin, RomanBun, PhilippeMuley, PranjaliBunge, Christian-AlexanderMullen, LindaBunyak, FilizMüller, AlexandruBuonocore, FrancescoMüller, AndreiBurattini, LauraMüller, GerhardBurger, ThomasMüller, IngmarBurgos, NinonMulvana, HelenBuric, MichaelMumolo, EnzoBuriro, AttaullahMumtaz, ShahidBurmistrov, I.N.Munari, MarcoBurnett, Andrew D.Muñoz, Carlos Quiterio GómezBurnos, PiotrMuñoz, JoseBurrascano, PietroMuñoz-Barreto, JonathanBurri, SamuelMuñoz-Gea, Juan PedroBursi, LucaMunoz-Organero, MarioBurud, IngunnMunschy, MarcBurugupally, Sindhu PreethamMunster, PetrBuscaglia, MarcoMunteanu, Florentina-DanielaBuse, HelenMunz, MichaelBustillo, AndresMura, AndreaButeau, SylvieMuralikrishnan, BalaButrymowicz, DariuszMuratori, MatteoButt, HaiderMuravyov, Sergey V.Butta, MattiaMurayama, RiichiBystrov, AleksandrMurimi, RenitaByun, Hyung-GiMuroyama, MasanoriCaballero, AntonioMurphy, MauriceCaballero, OlgaMurphy, MichaelCaballero, PinoMurray, NicholasCabboi, AlessandroMurroni, MaurizioCabral, JorgeMurtra, Andreu CorominasCabreira, TauaMurugan, Nirosha J.Cabrera, HumbertoMurvay, Pal-ȘtefanCabrera-Munoz, NestorMusić, JosipCaciolli, AntonioMusleh, BasamCaesarendra, WahyuMust, IndrekCaffarri, StefanoMustapha, SamirCaggianese, GiuseppeMuszyński, TomaszCaggiano, AntonioMutka, AlanCai, BaopingMutlu, RahimCai, HongmingMütze, TorstenCai, JieMydlarz, CharlieCai, JinguangMyler, Harley R.Cai, JinhaiMylonas, GeorgiosCai, ZhongyuMylvaganam, SabaCaiani, EnricoMyre, JosephCaimmi, MarcoNa, Wongi SCain, StephenNabok, AlexeiCaja, GerardoNaccarato, Kleber PinheiroCakmak, OnurNađ, ĐulaCalabretta, Maria MaddalenaNadeem, TamerCalabrò, VincenzoNadendla, Venkata Sriram SiddhardhCalado, João M. F.Nadolny, ZbigniewCalbimonte, Jean-PaulNaeem, UmairCaldas, PauloNagai, KoheiCalderoni, LucaNagano, HanatsuCaleffi, MarcelloNagao, ToshiyasuCalì, MicheleNagaraja, ShishirCaliano, GiosueNagatani, NaokiCallico, Gustavo M.Nagayama, TomonoriCalore, EnricoNaghshvarianJahromi, MahdiCalveras, AnnaNagle, AnnaCalvo, RoqueNagles, EdgarCalvo-Lobo, CésarNaik, Ganesh RCalvo-Zaragoza, JorgeNaimi, MohamedCalzada, IgorNaït-Abdesselam, FaridCamacho, DavidNajafi, BijanCamara, CarmenNajibi, NasserCamarinha-Matos, LuisNakagawa, KeiCambareri, ValerioNakamiya, ToshiyukiCambou, BertrandNakamura, AtsushiCamp, CharlesNakanishi, MasakiCampanella, Carlo EdoardoNakano, KimihikoCamparo, JamesNakano, MichihikoCampbell, CarleneNakano, TadashiCampbell, Sawyer D.Nakata, ToshiyukiCampo, CelesteNakayama, YuCampolo, ClaudiaNalepa, JakubCampopiano, StefaniaNam, Doan Ngoc ChiCampos, Carlos Alberto VieiraNam, InhoCampos, RicardNam, In-HyunCampos-Taberner, ManuelNama, NiteshCamps, AdrianoNamiesnik, JacekCampuzano, SusanaNamieśnik, JacekCamuñas-Mesa, LuisNamiot, DmitryCamurri, MarcoNanayakkara, ThrishanthaCandeias, PauloNanni, LorisCandelieri, AntonioNano, AdelaCanet Ferrer, JosepNanver, Lis K.Cañete, Francisco JavierNanzer, JeffreyCannistraro, SalvatoreNapa, Sae-BaeCano Garcia, Jose ManuelNapoletano, PaoloCano, AlbertoNaqvi, Syed MohsenCano, Juan-CarlosNar, FatihCano, Maria DoloresNarakathu, Binu BabyCanovas, AlejandroNaranjo Hernández, José EugenioCantisani, GiuseppeNaranjo, Jose EugenioCanut, Carlos GranellNaranjo-Hernández, DavidCao, BingqiangNarayanan, AjitCao, ChangyongNarayanan, Ram M.Cao, HungNardelli, MimmaCao, JindeNardini, GiovanniCao, NingNardone, RobertoCao, QunshengNarducci, FabioCao, YueNaseer, NomanCao, ZhangNaser, M. Z.Cao-Paz, Ana MaríaNashed, Youssef S.G.Capella, Juan VicenteNäsholm, Sven PeterCapizzi, GiacomoNasimuddin, N.Capobianco, GiuseppeNasir, SaeedCapoen, BrunoNasiri, NoushinCaponero, Michele ArturoNasiriavanaki, MohammadrezaCaporale, DaniloNaskar, SusmitaCaporaso, NicolaNäslund, Lars-ÅkeCapozzoli, LuigiNassar, SamehCapra, AlessandroNatarajan, MaheshCapuano, RosamariaNatsuaki, RyoCarabin, GiovanniNava, GiovanniCarballar, AlejandroNavarro Martín, JoanCarbonaro, NicolaNavarro Pedreño, JoseCarbone, GiuseppeNavarro, ElenaCardenal, JavierNavarro, GabrielCardenas-Juarez, MarcoNavas-Cortés, Juan A.Cardoso, JorgeNawrat, AleksanderCarette, JérômeNaya Villaverde, Miguel ÁngelCarey, BenjaminNazeer, MajidCarfora, Maria FrancescaNdieyira, JosephCaricato, Anna PaolaNeagoe, MirceaCariou, ClaudeNeagu, GabrielCaris, MichaelNecsulescu, Dan-SorinCarletti, VincenzoNeculoiu, DanCarlson, Blair E.Negri, Rogério GalanteCarlson, Daniel FrazierNeipp, CristianCarminati, MarcoNekrasov, AlexeyCarmo, JoãoNele, LuigiCarmona-Duarte, CristinaNelson, Alexander H.Carneiro, DavideNelson, Jill K.Carneiro, GlaucoNelson-Wong, ErikaCarnero Moya, María Del CarmenNemala, HumeshkarCarotenuto, VincenzoNemestothy, NandorCarp, StefanNeri, GiovanniCarrabs, FrancescoNeri, PaoloCarrasco, AlejandroNeubert, JeremiahCarreno-Luengo, HugoNeuenschwander, JuergCarrino, FrancescoNeumann, ClausCarrio, AdrianNeumann, HeikoCarrion, DanielaNeumann, HolgerCarro Fernandez, GermánNeumann, IngoCarroll, MarkNeumann, Patrick P.Carron, AndreaNeuner, HansCartalis, ConstantinosNevat, IdoCaruso, AntonioNeveln, IzaakCarvajal, FernandoNewe, ThomasCarvalho Monteiro, Ricardo NunoNewman Frey, Kimberly E.Carvalho, AshwinNex, FrancescoCarvalho, DanielNg, BrianCarvalho, LidiaNg, Derrick Wing KwanCarvalho, PauloNg, Eldwin JiaqiangCarvalho, VítorNg, JosephCasalini, StefanoNg, NormanCasalino, GabriellaNgo, Ha-DuongCasals, AlíciaNgo, Trung ThanhCasals, OlgaNguyen Thanh, KienCasanella, RamonNguyen, Binh P.Casari, PaoloNguyen, ChuongCasas Ferreira, Ana MaríaNguyen, Dat TienCasas, Francisco JavierNguyen, Duong VanCasas, OscarNguyen, HungCasas, RobertoNguyen, Linh VietCasaseca, PabloNguyen, Nam-TrungCasavola, AlessandroNguyen, Ngoc A.Casciati, FabioNguyen, Ngoc HungCasciati, SaraNguyen, Ngoc TuCaselli, FedericaNguyen, NhaCases Rius, Joan RamonNguyen, TamCasilari Pérez, EduardoNguyen, Tam Q.Casimiro, AntónioNguyen, Thien D.Caso, GiuseppeNguyen, TrieuCasquel, RafaelNguyen, Tu (Ryan)Cassidy, John F.Nguyen, Van DungCasson, AlexNguyen-Trong, NghiaCastagna, RossellaNiaz, Muhammad TabishCastagnetti, CristinaNiazi, Imran KhanCastagnola, ElisaNic Chormaic, SileCastaldi, FabioNiccolai, AlessandroCastano, Lina M.Nicholas, ChristofidesCasteleiro-Roca, Jose LuisNichols, AndyCastiglione, AnielloNico, GiovanniCastiglione, ArcangeloNicodemo, GianfrancoCastilla, GuillermoNicolae, MaximilianCastillejo Parrilla, PedroNicolaescu, IoanCastillo Sequera, José LuisNicolaou, NicolettaCastillo, EncarnacionNicolau, Maria JoãoCastillo, Luis FernandoNicolay, PascalCastro Do Amaral, GustavoNie, LimingCastro, MickaëlNiederleithinger, ErnstCasu, FrancescoNielsen, Boye SchnackCasula, GiuseppeNielsen, JohnCatalà Mallofré, AndreuNiemi, TapioCataldo, RosellaNieuwenhuisen, MatthiasCataliotti, AntonioNiggemann, OliverCatherwood, Philip A.Nightingale, AdrianCatry, ThibaultNiitsu, KiichiCattelani, LucaNikas, George K.Cava, José Manuel GonzálezNikolaev, ViacheslavCavalcante, Charles CasimiroNikolaidis, IoanisCavallari, Marco RobertoNikolaidou, MaraCavallini, AndreaNikolakopoulos, KonstantinosCavenaghi, Marcos AntônioNikolakopoulos, PantelisCazorla, MiguelNikolaou, SymeonCecchi, StefaniaNikolelis, Dimitrios P.Cechowicz, RadosławNilghaz, AzadehCecílio, JoséNilsson, Hans-ErikCefalas, A.C.Nilsson, TobiasCegla, FredericNimmagadda, Shastri L.Celaya-Padilla, José M.Ning, ZhiCelebi, EmreNino, PasqualeCelenk, MehmetNinomiya, SeishiCellini, FilippoNiroumand-Jadidi, MiladCenedese, AngeloNishida, HitoshiCennamo, NunzioNishio, MayukoCenten, PeterNishio, YoshifumiCeramicola, SilviaNissen, IvorCercenelli, LauraNissinen, JanCerdà, ArtemiNita, Bogdan G.Cerda, FernandoNittel, SilviaCernadas, EvaNitti, MicheleCerny, TomasNiu, SimiaoCerotti, DavideNivedita, NiveditaCerra, DanieleNoborio, KosukeCerri, Marcel OtavioNocerino, EricaCerro, GianniNogales-Bueno, JulioCervantes, EmilioNoghanian, SimaCervantes, JairNolan, HughCervantes, MichelNomura, YoshihikoCervigón, RaquelNorgia, MicheleCetin, Kristen S.North, Max M.Cetinkaya, OktayNosrati, MehdiCetó, XavierNothwang, WilliamCh. Miliaresis, GeorgeNotti, DavideCha, Young-JinNouretdinov, IliaChacón, JesúsNouri, HassanChadli, MohammedNoussan, MichelChaerle, LauryNovac, Bucur-MirceaChahl, JavaanNovak, EdChai, JiajueNovák, PetrChakeri, AlirezaNovellino, AlessandroChakrabarty, AnjanNowak, AleksanderChakraborty, ImonNowel, KrzysztofChalioris, ConstantinNowicki, MichałChamonine, MikhailNowicki, Michał R.Chamoso, PabloNowosielski, AdamChan, Chia-TaiNozato, HideakiChan, JeffersonNozick, VincentChan, Kam Wai CliffordNtotsios, EvangelosChan, PakNtouskos, ValsamisChan, Yu-WeiNuchkrua, ThananaChandhar, PrabhuNumkam Fokoua, EricChandrawati, RonaNunes, FernandoChang, Chao-TsunNuñez Andrés, AmparoChang, Chia-ChenNúñez Vicencio, AlfredoChang, Chih-YungNuñez, NeftaliChang, Dah-ChungNunziata, FerdinandoChang, Guo-EnNykiel, GrzegorzChang, Herng-huaO’Mahony, ConorChang, Hsueh-HsienO’Rorke, RichardChang, Jin HoObara, BoguslawChang, JunOberholster, AbrieChang, Kai-ChunObien, Marie EngeleneChang, Kang-MingOboe, RobertoChang, Kuo-JenO’Brien, Casey P.Chang, LeonardoOcaña, ManuelChang, MichaelOchoa-Zezzati, AlbertoChang, Min-KuanOćwieja, MagdalenaChang, Pei-ZenOesterschulze, EgbertChang, Sang-YoonOgawa, ShinpeiChang, Seung-HwanOggeri, ClaudioChang, Sheng-PoOggioni, AlessandroChang, Shu-WeiOgi, KeijiChang, Soon-JyhOgihara, NaomichiChang, Tai-WooOh, Beom-SeokChang, Te-WeiOh, Byung KwanChang, TianyingOh, DaegunChang, Wan-JungOh, EunkeuChang, Won-DuOh, Han BinChang, XiaojunOh, Ki-YongChang, Yau-ZenOh, SechangChang, Young KiOh, Seung JaeChang, Yu-ChungOh, Soong JuChan-Hon-Tong, AdrienOh, Tae KeunChao, HuikuanOhgi, YujiChappel, EricOhneiser, OliverChardonnens, JulienOhno, SeigoChargé, PascalOhrhallinger, StefanCharlton, PeterOikonomou, KonstantinosCharrier, JoelOishi, Andrew C.Chartier, AgnesOjha, Varun KumarChaspari, TheodoraOkarma, KrzysztofChateauneuf, AlaaOkazaki, ShinjiChatti, KiranamO’Keefe, KyleChaturvedi, ItiOken, Barry S.Chaturvedi, PracheeOkita, TsuyoshiChatzakis, JohnOkumura, ShiroChatzigiannakis, IoannisOlaizola, Igor GarcíaChau, Kwok-wingOlaru, AndreiChau, Lai-KwanOlatunbosun, Oluremi AyotundeChau, PhucOlaya, Angel GarcíaChaudet, ClaudeOldeland, JensChaudhury, AyanOletic, DinkoChava, Rama KrishnaOlfs, Hans-WernerChavanne, XavierOliva, GabrieleChávez, EstebanOlivares, JimenaChe, WeitaoOliveira, AnabelaChecchin, PaulOliveira, Francisco P.M.Chen, XiangdongOliveira, João P.Chen, BaojunOliveira, LuísChen, BinbinOliveira, RicardoChen, BoOliveri, AlbertoChen, Bo-HaoOliviero, MassimoChen, Bor-SenOloumi, DanielChen, ChenOlson, BritaChen, ChiOlson, Colin C.Chen, ChiachungOlson, Elizabeth S.Chen, Chien-yuanOlson, Michael W.Chen, Chi-HuaOlszewski, RobertChen, Chuh-YungOlthuis, WouterChen, Chun-TaOmura, YasuhisaChen, CunjianOmurtag, AhmetChen, DaiqinOneto, LucaChen, Der-chinOniga, FlorinChen, FeiO’Nils, MattiasChen, Gang S.Ono, KanjiChen, GuanboOno, YumieChen, GuangxueOnorati, TeresaChen, HaoyuanOppegaard, BrettChen, HongyangOprea, SimonaChen, Hsing-ChungOpromolla, RobertoChen, Hsiu-MeiOrbach, RonChen, HuaiqiongOrbaek White, AlvinChen, Huei-Yen WinnieOrciuoli, FrancescoChen, HuiOrdieres-Meré, JoaquínChen, JiaweiOrdoñez Morales, Francisco JavierChen, JunO’Reilly, Martin A.Chen, JunpingOrlando, DaniloChen, Jyh-JianOrozco Barbosa, LuisChen, Kuan-FuOrozco, JahirChen, Kun-YungOrozco-Lugo, Aldo GustavoChen, LiangOrsini, AndreaChen, Liang-BiOrsino, AntoninoChen, LiangyinOrtega, BeatrizChen, Lien-WuOrtega, JulioChen, Ling-jyhOrtega-Casanova, JoaquínChen, LongweiOrtegon, JaimeChen, Lung-ChienOrth, AntonyChen, NanOrtiz, JesusChen, PengOrtolani, MarcoChen, Qi AlfredOrtolani, MicheleChen, QianOsaba, EnekoChen, QiushuiOsen, Ottar L.Chen, Roland K.O’Shea, TimChen, RuiminOstasevicius, VytautasChen, SanjianÖsterberg, PatrikChen, Shiuan-YehOstovar, SaeidehChen, SiOstrowska, KseniaChen, Ssu-HanOsuch, TomaszChen, Sung-LiangO’Sullivan, ThomasChen, WenxiOsuna-Enciso, ValentínChen, Whai-EnOswald, BeateChen, WuOswald-Tranta, BeateChen, XianfengOsypiuk, RafałChen, XiaomingOszust, MariuszChen, XingOtebolaku, AbayomiChen, XudongOterkus, ErkanChen, XueminOtero, PabloChen, XunOtnes, RoaldChen, Ya-ShuOton, ClaudioChen, YawenO’Toole, John M.Chen, Yen-DaO’Toole, MatthewChen, Yen-LinOtremba, ZbigniewChen, Yeou-JiunnOtsuka, YuichiChen, Yi-ChaoOtte, MichaelChen, YizhengOu, JianzhenChen, Yuan-TsungOu, ShumaoChen, Yu-HsuanOu, Ting-ChiaChen, Yung-YuOuahabi, M. AbdeldjalilChen, ZhenOuchi, KazuoChen, ZhenghuaOuk, Tan-SotheaCheneler, DavidOuyang, BingCheney, MargaretOuyang, LiangqiCheng, BingbingOuyang, MengxingCheng, ChengØvstedal, OlaCheng, ChiachiØvsthus, KnutCheng, Chiang-hoOwens, Róisín M.Cheng, Chien-FuOyamada, YujiCheng, Huanyu (Larry)Ozawa, AkiraCheng, JuanOzawa, HiroshiCheng, Jun-HuOzer, EkinCheng, NanOzevin, DidemCheng, QiangÖzger, MustafaCheng, Wood-hiOzgur, ErolCheng, YongcunP. Mathur, AdityaCheng, ZhiqiangPaar, GerhardChernbumroong, SaisakulPaavola, MarkoCheruzel, LionelPacchierotti, ClaudioChessa, ManuelaPacheco Tanaka, David AlfredoChessa, StefanoPacheco-Blanco, BélgicaChevalier, Francois LePacheco-Peña, VictorChevalier, PascalPaczosa-Bator, BeataCheynet, EtiennePadalkar, SonalChhatkuli, AjadPadmanabhan, JagannathChiadò, AlessandroPadmanabhan, PreethiChiang, Cheng-TaPados, Dimitris AChiang, Chen-KuoPadrón, Luis A.Chiang, Chen-YuPaek, JeongyeupChiang, Chia-ChinPaffenroth, RandyChiang, Jung-ShengPagana, GuidoChiang, Kai-WeiPage, AlexChiatto, MatteoPagès-Zamora, AlbaChiavaioli, FrancescoPagonis, Dimitrios – NikolaosChien, HwaPaheding, SidikeChien, Jun-ChauPahl, ClausChien, Ying-RenPaik, Soon YoungChifu, Viorica RozinaPaiva, SaraChilo, JoséPaixão, AndréChilson, PhillipPajewski, LaraChilvery, AshwithPajovic, MilutinChin, Bryan A.Pak, AlexeyChin, Cheng SiongPak, SoojongChin, LixinPakrashi, VikramChindamo, DanielPal, AmitangshuChing, Congo Tak ShingPalacios-Laloy, AgustinChintakunta, HarishPalacios-Navarro, GuillermoChiriacò, Maria SerenaPalacz, MagdalenaChirico, AlicePalágyi, KálmánChiu, Long SPalamartchouk, KirillChiu, Nan-FuPalanisamy, SelvakumarChiu, YiPalazzi, ValentinaChiueh, Pei-TePalego, CristianoChizari, HassanPalermo, EduardoChizhik, Alexey I.Paleu, ViorelChmelik, FrantisekPałka, NorbertCho, AmPalla, MirkóCho, Byoung-KwanPallotta, LucaCho, ChungyeonPalma, FabrizioCho, ChunheePalmeri, RobertaCho, GihwanPalmerini, Giovanni B.Cho, HansangPalmieri, MarcoCho, HyuntaePalumbo, DavideCho, MyungjinPalumbo, FelixCho, Seong YunPalzer, StefanCho, SoojinPan, DaCho, SungraePan, GuangyuanCho, Yong SooPan, Huang HsingCho, Young ImPan, JianliChoi, Bong JunPan, PengminChoi, Cheon WonPan, QinChoi, Dong-HoonPan, ShijiaChoi, HeungjaePan, Tung-MingChoi, InheePan, W. DavidChoi, JaehyukPan, XueniChoi, Jee WoongPan, ZhigangChoi, Jeong-WooPanagiotakis, CostasChoi, JihoonPanagou, EfstathiosChoi, JongeunPanaousis, EmmanouilChoi, JungilPanaras, GiorgosChoi, JunilPanasiti, Maria SerenaChoi, Kup-SzePanat, RahulChoi, Min-HyungPancrazio, JosephChoi, OukPandarese, GiuseppeChoi, Seung-BokPandey, GauravChoi, SujiPandey, PremChoi, Woo-SeokPandey, RishikeshChoi, YerimPandith, AnupChoi, YoungcholPandya, AbhilashChon, Ki H.Panfilo, GiannaChong, AlvinPang, Da-ChenChong, Jo WoonPang, FufeiChong, See YennPang, ShintaroChoo, Pei LingPang, XiaobingChortos, AlexPani, DaniloChorzepa, Mi G.Panousopoulou, AthanasiaChou, Hsi-TsengPantano, MariaChou, Jung-ChuanPantazi, Xanthoula EiriniChou, Shih-FengPantoli, LeonardoChou, Tien-YinPanwar, NishthaChou, Tse HengPaoletti, PaoloChoudhury, SalimurPaolo, FavaliChouhan, Shailesh SinghPaolozzi, AntonioChouinard, Jean-YvesPapa, UmbertoChoukulkar, AdityaPapadakis, NikolaosChow, CaseyPapadavid, GeorgeChow, Chi-WaiPapadimitriou, Konstantinos I.Christian, EitzingerPapadopoulos, FragkiskosChronopoulos, Spyridon K.Papadopoulos, Georgios Z.Chrpa, LukasPapadopoulos, TheofilosChrysoulas, ChristosPapaefstathiou, Giannis S.Chryssolouris, GeorgePapaelias, MayorkinosChu, Cheng-ShanePapageorgas, PanagiotisChu, JinkuiPapaj, JanChu, Ta-ShunPapakonstantinou, ApostolosChua, BeeleePapalou, AngelikiChua, Ming YamPapoutsidakis, Michail G.Chuang, Cheng-HungPappalardo, Carmine MariaChuang, Kuo-ChihPappas, Odysseas A.Chuang, Yung-ChungParada Medina, RaúlChui, Hsiang-ChenParada, RaulChui, Kwok TaiParaforos, Dimitrios S.Chum, JaroslavParasuraman, RamviyasChun, HongguParedes, FerranChun, SejongParedes, HugoChung, IlyongParedes, Jose A.Chung, Moo K.Paredes, José ManuelChung, SungwonParedes-Madrid, LeonelChung, Tein-Yaw DavidParekh, SapunChung, Yao-LiangParente, ClaudioChung, YongwhaPareschi, FabioChurnside, JamesPariota, LuigiCiaccheri, LeonardoParis, ClaudiaCiampalini, AndreaParis, ClaudioCiampolini, PaoloPark, ByungwoonCianca, ErnestinaPark, Chan WonCicchetti, RenatoPark, ChansikCichon, JacekPark, Deog-SuCicone, AntonioPark, Dong SunCidronali, AlessandroPark, Jae-HyeungCiecholewski, MarcinPark, Jae-HyoungCieplak, MaciejPark, JaehyunCieslak, JérômePark, JaesikCięszczyk, SławomirPark, Jang SikCigna, FrancescaPark, Jang-UngCikajlo, ImrePark, Jee WoongCimalla, VolkerPark, Jong WoongCintuglu, Mehmet H.Park, Jong-SeungCioara, TudorPark, Jung-DongCiobanu, Radu IoanPark, Kang RyoungCionca, VictorPark, Kwi-IlCirillo, AndreaPark, Kyoung YoulCiti, LucaPark, NoseongCiuc, MihaiPark, PangunCiuciu, IoanaPark, Sang YoonCiuffoletti, AugustoPark, SangjoonCiuonzo, DomenicoPark, SehyunCiupala, Mihaela AncaPark, SeungheeClancy, EoinPark, Suk-HeeClark, CasparPark, Tae HwanClark, JamesPark, TaejinClarke, Keith C.Park, YongwanClarke, ThomasParkinson, DilworthClaßen, MartinParmehr, Ebadat GhanbariClaudel, ChristianParmiggiani, AlbertoClaudio, SavaglioParnandi, AvinashClayton, DwightParnianpour, MohamadCleary, KevinParolo, ClaudioCleij, Thomas J.Parra, LorenaCleland, Thomas A.Parrein, BenoitClement, BenoitParrein, BenoîtClément, HébertParrilla, LuisClement, PierrickParrinello, SandroClement, YuenParry, DaveClementi, FrancescoParthasarathy, SrinivasClevers, JanParziale, NickClifford, GariPasadas, Dario J.Climent-Pérez, PauPascoli, Stefano DiClosas, PauPascolo, PaoloClose, Dan M.Pascual Espada, JordanCluff, KimPascucci, SimoneCobianu, CornelPasero, ErosCobos, MaximoPasinszki, TiborCoburn, Craig A.Pasluosta, CristianCoca, AitorPasolini, GianniCocconcelli, MarcoPaspallis, NearchosCocolios, Thomas EliasPasquier, ThomasCoelho, João M. P.Passerone, RobertoCoelho, João PintoPassian, AliCohen, EliahuPasternak, GrzegorzCohen, RamiPastor, DanielCohenour, CurtisPastorelli, StefanoCola, AdrianoPata, PetrCole, ColinPatanè, FabrizioColla, ValentinaPatchay, SandhiranColl-Font, JaumePatel, Nirav NikunjCollings, NeilPatel, PankeshCollins, Christopher M.Patel, SanjayCollotta, MarioPatel, SaroshColombani, JeanPatel, SaurinColonnese, StefaniaPatela, SergiuszColosi, Horațiu AlexandruPateraki, MariaColuccia, AngeloPaterson, MauraColuzzi, RosaPatias, PetrosComby, FrédéricPatil, Ashok KumarComeau, FrankPatil, DevendraComeron, AdolfoPatil, MadhavComite, DavidePatras, PaulCommuri, SeshPatricio, JorgeCompagnone, DarioPatrick, ErinCompter, JohnPatrikakis, CharalamposConchon, EmmanuelPattanaik, HimansuConcilio, AntonioPatterson, ZacharyConde, Manuel FuentesPatti, DavideCondoluci, MassimoPau, AnnamariaConforto, SilviaPau, GiovanniCong, LongqingPauk, JolantaCongedo, Paolo MariaPaul, AnanyaConmy, RobynPaul, GeorgeConsani, CristinaPaul, Jonathan D.Conte, LanfrancoPaulette, LauraConteduca, DonatoPaulis, Francesco DeConti, AndreaPauliukaite, RasaConti, Antonia S.Paulo, Pedro M. R.Contreras, PedroPaulus, DietrichContreras-Ortiz, Sonia HelenaPaulus, Yannis M.Cookson, SimonPauly, KlaasCopic, DavorPaun, Maria-AlexandraCopot, CosminPavanello, FabioCoraluppi, StefanoPavelka, KarelCorchado Rodríguez, Juan ManuelPavliček, PavelCorchia, LauraPavlidis, IoannisCorcoran, PeterPaweł, PławiakCordeiro, CarlosPawlak, MichałCordera, RubénPawlak, RyszardCordero Fuertes, Juan AntonioPawlowski, AndrzejCordoba, AlbertoPawłowski, EligiuszCordoba, RicardoPayá, LuisCordolino Sobral, AndrewsPayan, CedricCoren, FrancoPayo, IsmaelCorgnati, LorenzoPazderski, DariuszCorigliano, AlbertoPaz-González, AntonioCorradi, FedericoPaziewski, JacekCorrales Ramon, Juan AntonioPearson, Matthew R.Correia, CarlosPecce, MarisaCorreia, SérgioPecorella, TommasoCorrigan, DamionPecori, RiccardoCorte, AntonioPedersen, Christian FischerCortellessa, GabriellaPedersen, HenrikCorujo, DanielPeham, ChristianCoskun, AhmetPei, LingCoskun, BulutPei, ZhichaoCosoli, SimonePei-Hsuan, TsaiCosta Rama, EstefaníaPeiner, ErwinCosta, Daniel G.Peláez-Moreno, CarmenCosta, Felipe DenardinPelc - Mieczkowska, RenataCosta, FilippoPelich, RamonaCosta, José A. F.Pelivanov, IvanCostabel, StephanPelka, MathiasCostafreda-Aumedes, SergiPellegrini, ChristineCostantini, FrancescaPellegrini, NicolaCostantino, DomenicaPellegrino, GiovanniCostanzo, AlessandraPellenz, Marcelo EduardoCostanzo, AntonioPellet, ClaudeCostanzo, SandraPelosi, PaoloCostello, Ben De LacyPelt, Daniël M.Couchot, Jean-FrançoisPeltokangas, MikkoCouper, KeithPena-Rios, AnasolCourault, DominiquePeng, ErwinCourtney, CharlesPeng, RuoguCoutinho, RodolfoPeng, XiCouto, Rodrigo S.Peng, Yan-TsungCovington, JamesPeng, YuexingCowling, MichaelPeng, YunCoxson, GregoryPeng, ZhengCozzolino, DanielPeng, ZhengyuCraciunescu, RazvanPeng, ZhenmingCragg, PeterPeng, ZhiliCragg, Peter JPennazza, GiorgioCraig, GregoryPenne, RudiCrandall, Aaron S.Pensieri, SaraCrane, Barbara A.Pepe, AntonioCretu, Ana MariaPepe, MassimilianoCrick, ChristopherPeppas, KostasCrilley, LeighPeppas, Kostas P.Crisan, Gloria CeraselaPerallos Ruiz, AsierCristea, CeciliaPeralta, DanielCristea, DanaPerego, PaoloCristina, Ana MajarenaPereira, EulaliaCrivelli, DavidePereira, Jean-MichelCrocco, LorenzoPereira, LucasCroce, Anna CletaPereira, Mauro FernandesCrosetto, MichelePereira, Olívia R.Crunteanu, AurelianPereira, Paulo RogerioCrussiere, MatthieuPereira, TâniaCruvinel, Paulo E.Perelli, AlessandroCruz, Homero ToralPerenzoni, MatteoCruz-Villar, Carlos-AlbertoPerera, RicardoCsillik, OvidiuPeres, António M.Csordas, AndrewPeres, EmanuelCsősz, ÉvaPeres, Henrique E. M.Ctistis, GeorgiosPeretti, RomainCtyroky, JiriPereyra, EduardoCuadras, AngelPérez Turiel, JavierCuartero, MaríaPerez, Aitor RoviraCucci, CostanzaPerez, AlfredoCuesta, EduardoPerez, RafaelCuesta, FedericoPerez-Castillo, RicardoCueva, Román Alcides LaraPérez-Cruz, JuanCui, BoruiPérez-Grande, IsabelCui, DixaoPérez-Juste, IgnacioCui, RongxinPérez-López, CarlosCui, YaoyaoPérez-Navarro, AntoniCui, YunxianPérez-Ocón, FranciscoCui, ZhaopengPerez-Ramirez, Carlos AndresCui, ZiqiangPerez-Ramirez, DanielCulshaw, BrianPerez-Uribe, AndresCulver, R. LeePergola, NicolaCummins, NicholasPergola, RNicolaCunha, MárioPerikos, IsidorosCurado-Teixeira, FranciscoPerini, RobertoCuriac, DanielPeris-Lopez, PedroCurran, James T.Perkins, NoelCürüklü, BaranPerotoni, Marcelo BenderCuypers, DieterPerrault, Katelynn A.Cwalina, KrzysztofPerreault, JonathanCyganek, BoguslawPerš, JanezCyr, FredericPersad, R.A.Czajewski, WitoldPersaud, KrishnaCzosnyka, MarekPersico, ValerioCzumbil, LeventePersson, AndersDa Costa, CesarPertusa, AntonioDa Costa, Daniel BenevidesPervan, Boris S.Da Costa, João PintoPescaru, DanDa Silva, José MachadoPesce, GiuseppeDa Silveira, MarcosPesch, Alexander H.Da Xu, LiPescosolido, LoretoDabbiru, LalithaPetcu, DanaDąbek, Paweł B.Peters, Gareth W.Dabrowski, Pawel S.Peters, Kara J.Daccache, AndrePeterson, JohnDagdeviren, CananPetit, NicolasDahlin, KylaPetkie, DouglasDai, GaoliangPetošić, AntonioDai, HaipengPetraglia, Mariane R.Dai, HongningPetráková, VladimíraDai, HoudePetrella, AndreaDai, JingPetrellis, NikosDai, NengliPetrič, TadejDai, ZhengfeiPetricevic, Slobodan J.Dai, ZhigaoPetropoulos, GeorgeD’Alessandro, AntonellaPetrou, PanagiotaDaliakopoulos, IoannisPetrovski, AndreiDalla Mora, AlbertoPetsa, ElliDalla Vedova, Matteo D. L.Petti, DanielaD’Aloia, MatteoPeuhkurinen, JussiDaly, KarenPeveler, WilliamDam, Van Anh T.Pevny, TomasDamaševičius, RobertasPeysakhovich, VsevolodDamkilde, LarsPezzotta, AlessandroD’andrea, CristianoPezzuolo, AndreaD’Andreagiovanni, FabioPfattner, RaphaelDanescu, RaduPfitzner, ChristianDanezis, ChrisPfost, MartinDanezis, GeorgiosPham, Duc TruongDang, VinhPham, Quoc-VietDang-il, ChoPham, Thinh HungDaniele, MichaelPham, Tien DatD’Aniello, GiuseppePham, Tuyen DanhDanielson, NeilPhan, Duy-ThachDanner, LukasPhan, Hoang-PhuongDantas, Mario A.R.Phan, HuyDante, AlexPhang, Swee KingDante, SilviaPhilipsen, Mark PhilipDao, Tien TuanPhillips, Andrew J. K.Dardanelli, GinoPhinyomark, AngkoonDarmon, MichelPhung, Quoc TriDa-Ronch, AndreaPhung, ToanDarsena, DonatellaPiancastelli, LucaDas, AnupPiao, XianjiDas, ArunPiazza, StephenDas, AruneemaPicard, DonaldDas, Diganta BhusanPicchio, RodolfoDas, JagotamoyPicco, LorenDas, SubhroPicco, LorenzoDatta, DebopamPicerno, PietroDatta, RupsaPichorim, Sergio FranciscoDau, VanPicone, MarcoDau, Van ThanhPięciak, TomaszDaughtry, Craig S.T.Pieczywek, Piotr M.Dautov, RustemPieraccini, MassimilianoDauvier, BrunoPieralice, FrancescaDavaji, BenyaminPieri, FrancescoDavalli, AngeloPierleoni, PaolaDavid, BeckerPieters, JanDavid, EliPifferi, ValentinaDavid, Jean-PierrePigatto, Daniel FernandoDavidrajuh, ReggiePigge, F. ChristopherDavidson, John W.Pignaton De Freitas, EdisonDavis, FrankPike, WilliamDavis, Tyler W.Piletsky, SergeyDavoli, LucaPilla, FrancescoDavydova, MarinaPillet, HeleneDawidowicz, KarolPilloni, VirginiaDawn, ArnabPillonnet, GaëlDawson, JohnPiltan, FarzinDe Alencar, Marcelo SampaioPimentel, RafaelDe Almeida, José Manuel M.M.Pina, PedroDe Angelis, AlessioPinar, AnthonyDe Angelis, GuidoPinchera, DanieleDe Arriba-Pérez, FranciscoPindado, SantiagoDe Bacco, CaterinaPing, JianfengDe Blasio, GabrielPingarron, JoseDe Bonis, AngelaPinhasi, YosefDe Cecco, MariolinoPinho, PedroDe Cubber, GeertPinkas, IddoDe Diego, Isaac MartínPiñol, PabloDe Dominicis, LuigiPinto, Andry MaykolDe Falco, PasqualePinto, Armando N.De Filippo, DanielaPiotrowski, ZbigniewDe Francesco, ErnestinaPiotto, MassimoDe Francesco, RaffaelePipa, Daniel RodriguesDe Freitas, Edison PignatonPirani, MohammadDe Freitas, Susana Isabel Pinheiro CardosoPirinen, PekkaDe Fuentes, Jose MariaPirjan, AlexandruDe Gaetani, CarloPiro, BenoîtDe Geest, BartPirola, MarcoDe Groot, Natasja Ms S.Piromalis, DimitriosDe La Blanca, Nicolas PerezPirozzi, SalvatoreDe La Cal, EnriquePisani, MarcoDe La Cruz Llopis, Luis J.Pisco, MarcoDe La Escosura-Muñiz, AlfredoPislaru-Danescu, LucianDe La Fuente, RaúlPissadakis, StavrosDe La Fuente-Nunez, CesarPistori, HemersonDe La O Serna, José AntonioPitarma, Rui AntónioDe La Torre Ibarra, Manuel H.Pitchappa, PrakashDe La Torre, ÁngelPitel, JanDe La Torre-Díez, IsabelPitkänen, TimoDe Lacerda, Raul L.Pitonakova, LenkaDe Lima Jr., Mauricio M.Pittella, ErikaDe Los Santos Álvarez, NoemíPitts, TeresaDe Luca, Anna ChiaraPiuzzi, EmanueleDe Luca, LuigiPivarčiová, ElenaDe Marchi, LucaPiyare, RajeevDe Maria, LetiziaPizzolante, RaffaeleDe Meij, Tim G.J.Pizzotti, MatteoDe Meo, PasqualePizzuti, StefanoDe Michele, CarloPlacidi, GiuseppeDe Miguel, ArgimiroPłaczek, BartłomiejDe Mingo Lopez, Luis FernandoPlaczek, MarekDe Monsabert, Benjamin GoislardPlatero, Carlos A.De Munari, IlariaPlatil, AntoninDe Nardis, LucaPławiak, PawełDe Oliveira, Mario AndersonPleissner, DanielDe Pascali, ChiaraPletersek, AntonDe Pauw, BenPleva, MatúšDe Poorter, EliPnevmatikos, NikosDe Sanctis, MauroPoco, JorgeDe Silva Indrasekara, AgampodiPodobnik, JanezDe Silva, VarunaPődör, AndreaDe Simone, Marco ClaudioPodržaj, PrimožDe Sousa Jr., Rafael T.Poenar, DanielDe Souza, Éfren LopesPogacnik, MatevzDe Stefano, LucaPoggi, GiovanniDe Vito, SaverioPoiesi, FabioDe, SuparnaPoigai Arunachalam, ShivaramDean Ben, Xose LuisPoisel, HansDean, KevinPolak, LadislavDebeir, OlivierPolanský, RadekDebela, Ahmed MehdiPolat, Emre OzanDebliquy, MarcPolcari, MarcoDeCoursey, ThomasPoletkin, KirillDefraigne, PascalePolicarpos, PapadopoulosDehghannasiri, RoozbehPolitis, ArchontisDehghan-Niri, EhsanPolo, FedericoDehzangi, OmidPolyakov, AlexanderDei, MichelePolydoros, AthanasiosDeilami, SaraPomares, SaúlDeinzer, FrankPomorski, DenisDeka, LipikaPompili, MassimoDel Campo, Francisco JavierPonce, HiramDel Din, SilviaPoncela, JavierDel Nogal Sánchez, MiguelPonomaryov, VolodymyrDel Río Celestino, MercedesPons, AlexanderDel Rio Fernandez, JoaquinPons, EnricoDe Silva, Liyanage ChandratilakPonta, LindaDel Valle Soto, CarolinaPontes, Maria JoseDel Vecchio, CiroPontin, AntonioDel Villar, IgnacioPonzoni Carvalho Chanel, CarolineDelaleau, EmmanuelPoonawala, Hasan A.Delalieux, StephaniePooranian, ZahraDelaney, Declan T.Poostchi, MahdiehDelbruck, TobiPop, Aniela I.Delcea, CameliaPopa, ValentinDelchev, KamenPopelka, StanislavDelebarre, ChristophePopescu, DanDelepine-Lesoille, SylviePopescu, Daniela-ElenaDelgado Prieto, MiguelPopescu, RadianDelgado-Gonzalo, RicardPopescu, VladDelibasis, Konstantinos K.Popoola, OlalekanDeligianni, DespinaPopov, PiotrDelikaris Manias, SymeonPopowicz, AdamDella Corte, FrancescoPoppinga, SimonDell’Amico, MauroPorcu, FedericoDellepiane, SilvanaPořízka, PavelDell’Olio, FrancescoPortelinha Júnior, Francisco MartinsDembele, SounkaloPorter, EmilyDemchenko, YuriPortillo, CarlosDemi, LibertarioPosada-Quintero, Hugo FernandoD’Emilia, GiulioPosadas-Castillo, CornelioDemir, NusretPoslad, StefanDemori, MarcoPost, Mark A.Den Hartog, FrankPostigo, Pablo AitorDendooven, PeterPostnikov, PavelDeng, JingPotenza, FrancescoDeng, QingxuPotika, KaterinaDeng, YingbinPotirakis, SteliosDenisov, Alexander N.Potortì, FrancescoDeniziak, StanisławPotter, HenryDenti, EnricoPottier, BernardDerlatka, MarcinPottier, EricDeRosa, ChristopherPoudel, Tej NarayanDeschinkel, KarinePoulakis, NikolaosDesmaris, VincentPound, MichaelDesmulliez, MarcPouresmaeil, EdrisDesroches, MaudePour-Ghaz, MohammadDestelle, FrancoisPourreza, AlirezaDev Jayant, RahulPous, NarcisDev, SoumyabrataPovinelli, Richard J.Devadas, Mary SajiniPownuk, AndrzejDevadoss, AnithaPowroźnik, PaulinaDevaraju, AnusuriyaPoza-Lujan, Jose-LuisDevkota, JagannathPozo, FrancescDevy, JérômePozza, RiccardoDewan, Ashraf M.Pozzebon, AlessandroDhakal, AshimPrabhu, RadhakrishnaDhakal, RajendraPradhapan, ParuthiD’Hondt, OlivierPrakash Kottapalli, Ajay GiriDi Bartolomeo, AntonioPrakash, RaviDi Bella, ClaudiaPramudianto, FerryDi Donato, LoretoPrandi, CatiaDi Flumeri, GianlucaPrasad, AbhnilDi Francesca, DiegoPrasad, Dilip K.Di Franco, CarmeloPrasad, MukeshDi Leo, MargheritaPrasad, VenkateshaDi Martino, FerdinandoPrasser, Horst-MichaelDi Martino, GerardoPratas, DiogoDi Natale, CorradoPrati, RonaldoDi Noia, Luigi PioPrato, MarcoDi Nuovo, AlessandroPrause, ChristianDi Paola, MarioPreciado, Miguel ADi Rienzo, LucaPrecup, Radu-EmilDi Sante, RaffaellaPreda, IonDi Simone, AlessioPredic, BratislavDi, HaibinPredoi, Mihai V.Di, JiangleiPreethichandra, PreethiDiakhate, MalickPrehofer, ChristianDiamant, RoeePreiner, DarkoDiamond, DermotPremebida, CristianoDianat, PouyaPrentkovskis, OlegasDiao, XiuminPreuveneers, DavyDias, PauloPreviati, MaurizioDias, Rosana AlvesPrezioso, GiuseppinaDíaz Nafría, José MaríaPřibilová, AnnaDiaz, Estefania MunozPriefer, RonnyDiaz-Barradas, Mari CruzPrieur, FabriceDíaz-Cruz, José ManuelPrigent, CatherineDibai-Filho, Almir VieiraPrijić, AnetaDiBenedetto, FrancescoPrimatesta, StefanoDickert, Franz L.Primiceri, ElisabettaDickson, D. BrucePrinz, Gary S.Dieber, BernhardPriori, SimoneDietzel, StefanProcek, MarcinDíez, Isabel De La TorreProcházka, AlešDíez, Luis FranciscoProchazka, DavidDiez, YagoProchniewicz, DominikDiGangi, JoshuaProffitt, RachelDiguet, Jean-PhilippeProkeš, AlešDilonardo, ElenaPronello, CristinaDimaki, MariaProvazník, IvoDimitrios, KogiasPrudenzano, FrancescoDimos, KonstantinosPryamikov, Andrey D.Dimoulas, CharalamposPryss, RüdigerDincer, CanPrzyborski, MarekDinesh, BhimareddyPrzybylek, PiotrDing, GuoruPrzybylski, MarekDing, MengPrzybyt, MałgorzataDing, WenboPrzysowa, RadosławDing, XiaorongPsarakis, Emmanouil Z.Ding, ZhaoyangPsarrou, AlexandraDinh, AnhPsimoulis, PanosDinh, KienPsuj, GrzegorzDinh, ThanhPtak-Chmielewska, AnetaDinh, ToanPu, CongDinis, RuiPu, XiongDinu, DanielPucci, AndreaDiou, ChristosPuchades, IvanDiraco, GiovanniPuche-Panadero, RubenDixit, YashPuente, SantiagoDjeziri, Mohand ArabPugi, LucaDjuric, AnaPuiu, MihaelaDjurović, IgorPujades, SergiDo Couto, António FidalgoPullanagari, Rajasheker R.Do, Phu ThinhPullano, Salvatore AndreaDobler, GregoryPuniach, EdytaDobosz, MarekPursley, BrennanDobre, CiprianoPushpavanam, KarthikDobre, OctaviaPustan, MariusDochshanov, AldenPutze, FelixDoerzaph, ZacharyPyo, SoonjaeDoeven, EganPyrchla, JerzyDogancay, KutluyilPyun, Jae-YoungDoglioni, AngeloQaisar, Sana U.Doh, NakjuQamar, ZeeshanDolabdjian, ChristopheQasaimeh, Mohammad A.Dolega, BoguslawQi, WangDomaszewicz, JarosławQi, YiDombek, GrzegorzQi, ZhixinDomijan, MirelaQian, Cheryl ZhenyuDomingues, InêsQian, XifengDomingues, Maria De FátimaQian, ZhenyunDominguez, Rocio B.Qiang, ZheDominguez, SergioQiao, YuansongDomino, Krzysztof A.Qiao, ZhijunDonati, ElisaQin, JieDonati, MassimilianoQin, SiDonelli, MassimoQin, YihengDoneux, ThomasQin, YongruiDong, BoxiangQin, ZhanDong, Chun-YuQin, ZhengruiDong, GuifangQiu, ChenDong, HaiQiu, ChenxiDong, JianzhiQiu, JianxiuDong, JunliangQiu, SenDong, KimQiu, TianshuangDong, LeiQiu, WeibaoDong, MingdongQiu, XianboDong, ShurongQiu, ZhenDong, TaifengQu, FengzhongDong, TaoQu, HaiboDong, WeiQu, HangDong, WenQu, HongweiDong, YingQuattrini Li, AlbertoDong, ZhenQue, LongDong, ZhuxinQueau, YvainDoni, Mihaela BadeaQuéau, YvainDonno, DarioQueluz, Maria PaulaDoran, DerekQuer, StefanoDorazco-González, AlejandroQuero, GiuseppeD’Orazio, TizianaQuero, José ManuelDorronzoro, EnriqueQuincey, PaulDos Santos, Alcindo A.Quinn, Mark KennethDos Santos, Eduardo NunesQuintana, Penelope.J.E.Dos Santos, Marco SoaresQuitadamo, LuciaDosen, StrahinjaQuynh Hoa, LeDouik, AhmedRabadan, JoseDoulamis, AnastasiosRabczuk, TimonDoulamis, NikolaosRabie, KhaledDouligeris, ChristosRabnawaz, MuhammadDouzals, Jean-PaulRackauskas, SimasDowrick, ThomasRácz, AnitaDoyle, Lucinda ElizabethRadac, Mircea-BogdanDoyle, Thomas E.Radecka, HannaDragic, PeterRadhakrishna, UjwalDrap, PierreRadkowski, RafaelDrapaca, CorinaRadonic, VasaDrapikowski, PawełRadu, AleksandarDrbohlavova, JanaRadwan, AymanDrese, Klaus StefanRaeis Hosseini, NiloufarDrewniak, SabinaRaffelsberger, ChristianDrews-Jr, PauloRafiq, Muhammad ImranDrexler, PetrRaghavan, NagarajanDrocco, MaurizioRagulskis, MinvydasDrost, BertramRahim, Md. Noor ADrusa, MariánRahman, Md ShihanurDu, ChangwenRahman, Md. MahbuburDu, DaweiRahman, MoshiurDu, E (Sarah)Rahman, MusfiqDu, JianhaoRahman, TauhidurDu, QianRahnejat, HomerDu, QingheRai, AshwinDu, YunchengRaiconi, AndreaDuan, HaibinRainieri, CarloDuan, JinmingRaith, StefanDuan, QiangRaj, SaurabhDuan, WeiliRajan, GinuDuan, XiyuRajapandiyan, PanneerselvamDuarte, FábioRajaraman, SivaramakrishnanDubbini, MarcoRajbhandari, SujanDubey, HarishchandraRajchowski, PiotrDubey, RameshwarRakocevic, VeselinDubey, RichaRaluca Maria, AileniDubuc, DavidRamachandran, AnupamDubuis, GuyRamachandran, K. I.Duchateau, NicolasRamalli, AlessandroDudczyk, JanuszRamanavicius, ArunasDudkowiak, AlinaRamapriyan, HampapuramDudley, SandraRamapriyan, Hampapuram K.Dufek, Janet S.Ramasso, EmmanuelDufresne, AnjaRambach, JasonDuguay, YannickRamchurn, Sarvapali D.Duka, AdrianRamella, GiulianaDulebenets, Maxim A.Ramini, AbdallahDumic, EmilRamirez Miquet, EvelioDumitru, Andra-CristinaRamirez, Juan ManuelDumstorff, GerritRamírez, WilsonDunai, LarisaRamirez-Paredes, Juan-PabloDunbar, AlanRamon Soria, PabloDunea, DanielRamos, HelenaDung, Le TheRamos, VictorDunik, JindrichRamos, VictoriaDuong, TrungRamos-Diaz, Jose MartinDupre, MatthieuRamos-Ortiz, GabrielDupuis, PascalRampa, VittorioDupuis, YohanRampun, AndrikDuraibabu, Dinesh BabuRamsköld, DanielDurán Acevedo, Cristhian ManuelRana, A.Duro Carralero, NatividadRanavolo, AlbertoDusny, ChristianRanjan, RajeevDutta, AniruddhaRanjit, SumanDutta, AyanRanjkesh, AmidDutta, GaurabRanmuthugala, DevDuvall, Rachelle M.Rantalainen, TimoDuy, Le ThaiRao, AravindaDvir, AmitRao, AshwinDyke, ShirleyRao, Raveendra K.Dylan, KobsarRao, SmithaDyo, VladimirRao, ZhushiDzantiev, Boris B.Raoux, MatthieuDziadak, BogdanRapiński, JacekDzidic, AlenRaposo, MariaDzimitrowicz, AnnaRaptis, IoannisDziurdzia, PiotrRaptis, TheofanisDziurzanski, PiotrRaquet, John F.Ebendorff-Heideprem, HeikeRäsänen, AleksiEbrahimi, AidaRascon, CalebEbrahimi, AmirRashedi, EhsanEbrahimkhanlou, ArvinRashkovska, AleksandraEconomou, AnastasiosRasmussen, Henrik K.Edelman, BradleyRasmussen, MichelleEdmonson, William WRaspopoulos, MariosEffenberg, AlfredRass, StefanEftekhar Azam, SaeedRassõlkin, AntonEgami, YasuhiroRasti, PejmanEgan, Paul F.Ratassepp, MadisEgorov, EgorRath, Jagat JyotiEguchi, KeiRathod, VivekEichhorn, MikeRaulin, Jean-PierreEickeler, StefanRauste, YrjoEisank, ClemensRauterberg, MatthiasEjsmont, JerzyRavankar, AbhijeetEkstrom, MikaelRavankar, AnkitEl Ioini, NabilRavì, DanieleElahi, HassanRawassizadeh, RezaElaksher, AhmedRay, AndrewElara, RajeshRay, BiplobElasha, FarisRay, Sayan KumarElbahhar, Fouzia BoukourRaya, RafaelElble, Rodger J.Raybaut, MyriamElbuken, CaglarRaysoni, Amit UEldhuset, KnutRaza, MohsinElfergani, Issa TamerRazevig, VladimirElfring, JosRazionale, Armando V.Elgendi, MohamedRazjouyan, JavadElhabian, Shireen Y.Razzaghi, RezaElhag, SamiRazzaque, Mohammad AbdurElhattab, AhmedRdzanek, Wojciech P.Elhoseny, MohamedRe, RebeccaElias, PanagiotisReale, FerdinandoElisa, KallioniemiReali Costa, AnnaElkhodr, MahmoudReátegui, EduardoEllis, DavidRebaudengo, MaurizioElmqvist, NiklasReboud, ChristopheEl-Osery, AlyRecchiuto, CarmineEl-Osery, Aly I.Rech, IvanElsayed, MohannadRedding, BrandonElshaw, MarkReddy Chittamuru, Sai VineelEl-shenawy, AhmedReddy, YenumulaElsts, AtisRedon, NathalieEltner, AnetteRedweik, PaulaEltoukhy, MoatazRefice, AlbertoEmadi, ArezooRegattieri, AlbertoEmrith, KhemrajReggiani, LucaEngelbrecht, JeanineReggiannini, MarcoEngelbrecht, RainerRegmi, BishnuEnglot, BrendanRego, GasparEnguita, José MaríaRego, PaulaEnoiu, Eduard P.Regterschot, RubenEnokibori, YuRegueiro, Carlos V.Enokido, TomoyaRehmani, Mubashir HusainEpifani, MauroReich, SiegfriedErdogmus, NesliReid, Russell C.Eremin, ArtemReid, Tyler G.R.Eren, FiratReig, CàndidEriksen, René LyngeReily, BrianErkoreka, AitorReina, Daniel GutiérrezErny, Guillaume L.Reinhardt, WolfgangErola, ArnauReinoso, JoseErsen, AliReinoso, OscarErtman, SławomirReis, Catarina I.Esashi, MasayoshiReis, João Carlos R.Escamilla-Ambrosio, Ponciano JorgeReis, Manuel J.C.S.Escobedo, CarlosReis, Nuno MEscolano, Sergio OrtsReljin, NatasaEscudero, CarlosRen, DahaiEsffahani, AlirezaRen, HaoEshel, NoamRen, LingyunEsmerian, KarekinRen, MingEspinilla, MacarenaRen, PinyiEspinosa, Hugo G.Ren, QiangEsposito, AntonioRen, ShunqingEsposito, ChristianRen, XimingEsteban, SegundoRen, ZhenEsteban-Fernandez De Avila, BertaRenard, Jean-BaptisteEstepa, RafaelRenaudin, ValérieEstêvão, João M.C.Renault, EricEsteves Da Silva, Joaquim C.G.Renevey, PhilippeEstevez, M. CarmenRenzi, PolyssenaEttlinger, AndreasRetscher, GüntherEven-Tzur, GiladReu, Phillip L.Everbach, Erich CarrReyes, Bersain A.Evsutin, OlegReyes, Pavel IvanoffEvtugyn, GennadyReyes-Ortega, FelisaEynian, MahdiReynaud, RogerEysel-Gosepath, KatrinRezaeieh, Sasan AhdiEzer, DaphneRezai, PouyaFabbri, BarbaraRezania, ShahabaldinFabbri, TommasoŘezník, TomášFabbrocino, FrancescoRezzoug, NasserFabbrocino, GiovanniRhee, HuinamFabelo, HimarRhudy, MatthewFabianowski, WojciechRiaza, AsuncionFabregat, RamonRibeiro Costa, Nuno AlexandreFaccini, FrancescoRibeiro Pinto, JoãoFaccio, GretaRibeiro, Artur LopesFacco, PierantonioRibeiro, EraldoFadda, MauroRibeiro, JoséFaez, SanliRibichini, RemoFafoutis, XenofonRicci, StefanoFagadar-Cosma, EugeniaRicciardelli, ElisabettaFagerstrøm, AsleRicciato, FabioFai A. Lo, KwongRichards, ChrisFai, Leung ChunRichards, DeborahFaigl, JanRichter, ChrisFaisal, Mustafa AmirRichter, PhilippFakiris, EliasRichtera, LukášFalcão, Ana PaulaRida, ImadFalcão, GabrielRifà-Pous, HelenaFalchi, FabrizioRiganti Fulginei, FrancescoFalco, GianlucaRighi, MarcoFalco, NicolaRighi, RodrigoFalconi, Mario ChristianRigoni, FedericaFaleiro, EduardoRiguidel, MichelFaleschini, FloraRijal, SantoshFaley, MichaelRiley, BryanFaller, JosefRiley, Kathryn R.Faller, Lisa-MarieRim, You SeungFalletti, EmanuelaRimeika, RomualdasFamaey, JeroenRinaldi, StefanoFamiano, MichaelRinaldo, RobertoFan, BinRinner, BernhardFan, DeliangRinnert, EmmanuelFan, JianxiRios-Figueroa, Homero V.Fan, MengbaoRiou, Laurent M.Fan, PingyiRipka, PavelFan, YuanchengRisi, MicheleFan, ZhaoyanRisitano, GiacomoFan, ZhengRisojević, VladimirFang, LeiRitchie, Matthew A.Fang, PengRivadeneyra, AlmudenaFang, Ronnie H.Rivalland, VincentFang, Shih-HauRivas Casado, MonicaFang, XudongRivera-Guillen, Jesus-RooneyFang, YihaiRizal, ConradFantozzi, SilviaRiziotis, ChristosFaraci, Francesca DaliaRizzardi, AlessandraFaragasso, AngelaRizzi, MariaFarahmand, FaridRizzo, GiuseppeFaralli, StefanoRizzolo, SerenaFaraut, GregoryRobert, JérémyFärber, BertholdRobert, OdolinskiFarias, GonzaloRobert, SzabolcsiFarias, MyleneRoberto, GasparriFarinella, GiovanniRobles, GuillermoFarkas, KataRobles, RamiroFarlik, JanRocca, FabioFarooq, MuhammadRocchitta, GaiaFarooque, Aitazaz A.Rocha, AlfredoFarshchian, MasoudRocha, JorgeFasano, AndreaRocha, Luís A.Fasel, BenediktRocio-Bautista, PriscillaFateixa, Sara Isabel AugustoRodrigues, HelenaFateri, SinaRodrigues, JoãoFatikow, SergejRodrigues, José MiguelFaucher, Sébastien P.Rodriguez Gonzalvez, PabloFazio, PeppinoRodriguez, DionisioFedeli, AlessandroRodriguez, Elkin FerneyFederici, StefaniaRodriguez, GeorgeFedorov, GeorgyRodriguez, J. Apolinar MuñozFeger, ReinhardRodríguez, Luis AcedoFei, BaoweiRodriguez, NancyFei, TaiRodriguez, NoelFeiertag, GregorRodríguez, Orlando CamargoFeist, DietrichRodriguez, Sergio AlbertoFekete, ZoltánRodríguez-Bernaldo De Quirós, AnaFeld, SebastianRodríguez-Delgado, MelissaFeldheim, VeroniqueRodríguez-Martín, ManuelFelici-Castell, SantiagoRodriguez-Mendez, Maria LuzFelipe Oliveira, SilvaRodriguez-Morales, FernandoFelis, IvanRodríguez-Pulido, Francisco J.Felisberto, PauloRodríguez-Reséndiz, JuvenalFeliu, VicenteRodriguez-Sanchez, CristinaFelix, Anderson AndréRoemer, FlorianFelski, AndrzejRoepcke, JurgenFemminella, MauroRoffo, GiorgioFeng, ChuangRogers, John E.Feng, DongmingRogier, HendrikFeng, GuodongRoh, ChanghyunFeng, QiboRohac, JanFeng, ShaojunRöhrig, ChristofFeng, YanmingRoig, Ignacio BoschFeng, YaozeRojas-Laguna, RobertoFensel, AnnaRojo-Álvarez, José LuisFenza, GiusseppeRoldán Gómez, Juan JesúsFerdyn-Grygierek, JoannaRoldán Pérez, JavierFereidountabar, AmirhosseinRolland, LucFerentinos, DinosRollins, AndrewFerguson, Frank T.Román-Castro, RodrigoFerhan, Abdul RahimRomaniello, RobertoFerin, GuillaumeRomano, PietroFerlin-Oliveira, SimoneRomanovskii, OlegFernandes, FábioRomeira, BrunoFernandes, FranciscoRomeo, AgostinoFernandes, HenriqueRomeo, LucaFernandes, João CatalãoRomero Cano, Victor AdolfoFernández Álvarez, PedroRomero Perales, ElenaFernández Caballero, AntonioRomero, Fernando CastañoFernández Carrobles, María Del MilagroRonen, ShukiFernandez, AlbertoRönnholm, PetriFernández, Óscar BelmonteRonzani, DanieleFernandez, RoemiRosa, Ambra R. DiFernández, TomásRosa, Isabel M.D.Fernández-Caramés, Tiago M.Rosalie, CedricFernandez-Carmona, ManuelRosario, DenisFernandez-Garcia, RaulRosati, SamantaFernández-Getino García, María JuliaRoscia, MariacristinaFernandez-Hernandez, JesusRoscini, LucaFernandez-Llatas, CarlosRose, Joseph L.Fernandez-Moral, EduardoRosi, AscanioFernández-Pacheco, Daniel G.Rosier, Peter F. W.Fernandez-Prieto, ArmandoRosner, DanielFernández-Soto, PedroRosolem, Joao BatistaFernandez-Veiga, ManuelRossella, FrancescoFernando, XavierRossi, FedericoFerrández-Pastor, Francisco JavierRossi, MassimilianoFerrando, IlariaRossi, Pierluigi SalvoFerrara, Maria AntoniettaRossi, RenéFerrarezi, RhuanitoRossi, StefanoFerrari, PaoloRossignol, JeromeFerrari, SilviaRosso, FedericaFerrari, VincenzoRosten, EdwardFerrario, Maria FrancescaRotariu, CristianFerraro, DavideRotariu, LucianFerraro, PietroRoth, BernhardFerré-Borrull, JosepRoth, Bradley J.Ferrein, AlexanderRoth, ChristianFerreira, BrunoRoth, KeelyFerreira, Hugo AlmeidaRoth, MichaelFerreira, João CanasRoth, Stephan V.Ferreira, JoaquimRothkrantz, LeonFerreira, LucasRoths, JohannesFerreira, Luís AndradeRouane, AmarFerreira, Marta S.Rouhani, HosseinFerreira, Rute A.S.Roumet, PierreFerrer, BelenRouse, ElliotFerrer, GonzaloRousseau, LionelFerrero Martim, FranciscoRoussos, EvangelosFerrero, Francisco JavierRout, BhimsenFerrero, RenatoRoy, AbhishekFerri, GiuseppeRoy, SpandanFerrigno, LuigiRoy, SurajitFerster, Colin JayRoy, SwarupFerus, MartinRozemeijer, JoachimFeuer, ArieRozga, PawelFiala, PavelRozman, JanezFiandrino, ClaudioRoztocil, JaroslavFiandrotti, AttilioRuan, HaowenFicco, MassimoRuano, AntonioFice, MartynRubagotti, MatteoFidalgo De Cortalezzi, Maria M.Rubio Alonso, HiginioFidalgo, EduardoRubio, ConstanzaFidan, BarisRubio, Jose De JesusFidani, CristianoRucco, RosariaFiebig, Uwe-CarstenRucka, MagdalenaFiedler, GoeranRudnitskaya, AlisaFiglus, TomaszRudykh, StephanFigueira, Rita BacelarRuelas, AdolfoFigueiredo Neto, Antônio MartinsRuff, AdrianFigueiredo, LinoRuffato, GianlucaFigueroa, KarinaRuffin, PaulFigueroa, MiguelRügamer, AlexanderFilchev, LachezarRühmer, DennisFilchev, Lachezar H.Ruhoff, AndersonFilip, RadimRuijters, DanielFilipe, PortelaRuiz Llata, MartaFilippeschi, AlessandroRuiz Rubio, LeireFilipponi, FedericoRuiz Zamarreño, CarlosFilippoupolitis, AvgoustinosRuiz-Armenteros, Antonio M.Filmer, M. S.Rumyantseva, Marina N.Fingas, MervRundo, FrancescoFinlayson, NeilRunnerstrom, EvanFiore, AlessandraRuotsalainen, LauraFiorentino, GabriellaRuppert, MichaelFiorentino, MicheleRuppert, Michael G.Fiorini, LauraRusak, ZoltanFirebaugh, Samara L.Ruschin, ShlomoFischer, GeorgRush, CindyFisher, AdrianRuss, JohnFisher, DanielRussell, ChristopherFister, IztokRusu, CristinaFit, NicodimRusu, EugenFitzpatrick, PaulaRusu, LilianaFlavell, RobertRůžek, RomanFleischer, HeidiRužić, IgorFleischmann, FriedrichRybarczyk, YvesFletcher, AndrewRydosz, ArturFletcher, KennethRyglicki, DavidFletcher, Steven J.Ryman, ErikFleyeh, HasanRyoo, Young-JaeFlorea, LauraRyu, HyunryulFlores Tapia, DanielRyuh, Young-sunFlores, GerardoRzepecka, ZofiaFlores, Jorge L.Rzhanov, YuriFlores-Abad, AngelSa, InkyuFlores-de-Santiago, FranciscoSaad, WalidFlores-Fuentes, WendySaadane, RachidFloriano, Andrés GarcíaSaadatnia, ZiaFlynn, DavidSaal, HannesFocsan, MonicaSabato, AlessandroFodor, MariettaSabban, AlbertFodor, Petru S.Saber, MohamedFoguel, Marcos ViniciusSaberioon, MehdiFolea, SilviuSabourin, ChristopheFolke, MiaSabuncu, Ahmet CanFonollosa Magrinya, JordiSaccomandi, PaolaFonollosa, JordiSachs, JuergenFonseca, JoséSacré, Pierre-YvesFontanelli, DanieleSadhu, AyanFontani, MarcoSadhu, BodhisatwaFonte, Cidália CostaSadjadi, FiroozFoong, ShaohuiSadok, Djamel F. HadjForaboschi, PaoloSadowski, ŁukaszFord, JohnSadre, RaminForeman, MatthewSaeed, BakhtiarForlines, CliftonSaeed, KhalidFormenti, FedericoSaeedi, RamyarFormisano, AntonioSafa, MeerFornaini, CarloŠafarič, RikoFornaro, ClaudioSafont, GonzaloFornaser, AlbertoSaga, SatoshiForouzanfar, MohamadSagan, VasitForsstrom, StefanSaggese, AlessiaFörster, AnnaSaha, AvijitFortino, GiancarloSaharan, LalitaFortuna, LuigiSaho, KenshiFortune, EmmaSahoo, JagrutiFosson, SophieSaijo, YoshifumiFoster, Christopher FosterSailhan, FrancoiseFoster, K. R.Sainz De Abajo, BeatrizFotia, LidiaSainz, NekaneFotiadis, DimitriosSaitoh, TakeshiFotopoulos, GeorgiaSakai, KenjiFoucher, SamuelSakai, TakenobuFoudeh, AmirSakalas, PauliusFoukalas, FotisSakellariou, MichaelFourati, NajlaSaki, FatemehFourlas, George K.Sakkalis, VangelisFourneau, Jean-MichelSalach, JacekFowler, BoydSalama, Khaled NabilFox, GlenSalamanca Miño, SantiagoFox, Neil I.Salamí, EstherFrabrizio, SantiSalamon, JustinFraddosio, AguinaldoSalamone, FrancescoFraga, Francisco J.Salanova Grau, Josep MariaFraga, Mariana AmorimSalaoru, IuliaFraga-Lamas, PaulaSalau, J.Fraile-Marinero, Juan-CarlosSalaün, PascalFrançais, OlivierSalazar, AddissonFrancalanza, EmmanuelSalazar, AugustoFrancesco, FerracutiSalazar-Torres, Jose De JesusFrances-Villora, Jose VicenteSalceda-Delgado, GuillermoFranchina, Flavio AntonioSaleem, Hussam SFrancis, DanielSalehi, MahsaFrancisco José, Sánchez SutilSales, SalvadorFranco, LeonardoSalguero, Alberto G.Francois, AlexandreSaliba, JacquelineFrançois, AlexandreSalido, DavidFrangi, AttilioSalinas Maldonado, CarlotaFranke, JörgSalmerón, José F.Fränti, PasiSalmi, AriFrappart, FrédéricSalmi, MikaFrattolillo, FrancoSalomoni, Sauro EmerickFravolini, Mario LucaSalowitz, NathanFrazão, OrlandoSaltaren, RoqueFreddi, AlessandroSaltas, VassilisFreddi, FrancescoSalvador, J.-PabloFredianelli, LucaSalvarani, NicolòFreina, LauraSalvati, DanieleFreitas, GustavoSalvatierra, RodrigoFrense, DieterSalvatori, RosamariaFresse, VirginieSalvi, DarioFrey, OlivierSalvi, GiampieroFridman, MotiSamanta, Pralok KumarFriedrich, Christoph M.Samaras, IoannisFriedt, Jean MichelSamek, OtaFritsch, DieterSametinger, JohannesFrogley, Mark D.Samhoundi, MohamedFröhlich, FlavioSamiappan, SathishkumarFromenteze, ThomasSamie, FarzadFrontoni, EmanueleSammut, CharlesFroriep, Ulrich PSampaolo, AngeloFrustaci, FabioSampietro, DanieleFryskowska, AnnaSamuel Malki Ebeid, EmadFu, ChunyunSamuel, Oluwarotimi WilliamsFu, HongSamuel, StanierFu, HuazhuSanchez Molina, Jorge AntonioFu, JichengSánchez, AlfredoFu, XiaomeiSanchez, Jean-baptisteFuchs, BenjaminSánchez, JoseFudickar, SebastianSanchez, Juanma LopezFugazza, CristianoSanchez, ManuelFuhrmann, ThomasSánchez, Manuel GarcíaFujdiak, RadekSanchez, NancyFujibuchi, ToshiohSánchez, René-VinicioFujinami, KaoriSanchez, VictorFujioka, TakahiroSanchez-Iborra, RamonFujisawa, TomotsumiSánchez-Monedero, JavierFujiwara, EricSanchez-Oro, JesusFujiwara, KoichiSánchez-Rodríguez, DavidFukuda, TakeshiSanchez-Rojas, Jose LuisFukushima, NorishigeSanchez-Salmeron, Antonio J.Funari, RiccardoSanchis-Cano, AngelFung, Wai-KeungSandak, AnnaFurferi, RoccoSandbakk, ØyvindFurnari, AntoninoSanders, JamesFursov, Vladimir AlekseevichSandner, ThiloFusco, AdeleSandoghchi, Seyed RezaFuse, TakashiSandre, OliverFusiek, GrzegorzSanduleac, MihaiFutagawa, MasatoSang, Sik YangG Menon, VarunSanislav, TeodoraGaal, MateSankowski, DominikGabai, RanSanli, AbdulkadirGabriel, Jose LuisSanmartin, PaulGabriel, VeluSanna, AndreaGachet Páez, DiegoSannino, GiovannaGaczynska, MariaSanphuang, VaritthaGade, MartinSan-Segundo, RubénGade, RikkeSant, PaulGadella, Theodorus WJSantamaria, Amilcare FrancescoGagliano, AntonioSantana, JoseGaglione, SalvatoreSantana-Mancilla, Pedro C.Gagneja, KanwalinderjitSantangelo, Maria FrancescaGai, KekeSanthi, B.Gaillot, Davy P.Santhiago, MuriloGajda, JanuszSantiago, IsabelGalante, FrancescoSantis, Michele DeGalan-Vidal, Carlos AndresSantoni, FrancescoGalas, RomanSantorelli, AdamGalati, GaspareSantos, AbelGalatus, RamonaSantos, Daniel Rodrigues DosGalbács, GáborSantos, HenriqueGalian, Raquel E.Santos, Jesús DanielGałka, JakubSantos, Juan M.Gallagher, KyleSantos, Luís PicadoGallardo, JesúsSantos, MatildeGallego, AntolinoSantos, RodrigoGallego, Jose RamonSantos, TelmoGallegos, JavierSantosa, HendrikGallegos-Funes, Francisco J.Santoso, FendyGallina, AlbertoSanwar Hosen, A. S. M.Gallo, FrancescoSanz Bobi, Miguel AngelGallo, MarianoSanz-Ablanedo, EnocGalmes, SebastiaSapena-Baño, AngelGalopin, NicolasSaponara, SergioGalstyan, VardanSarbishei, OmidGalvan-Tejada, Carlos EricSardesai, NaimishGalve, Joan MiquelSaremi, MehdiGamage, KelumSargolzaei, ArmanGambi, EnnioSarigiannidis, PanagiotisGamboa, HugoSarjaš, AndrejGan, DianeSarkar, ChayanGandhi, VaibhavSarkar, SoumicGandji, Navid P.Sarkar, TapanGandolfi, StefanoSarraguça, MafaldaGanesan, BaskaranSarras, IoannisGanim, ZiadSarry, FrédéricGans, Nicholas R.Sartori, LuigiGanti, Raghu K.Saruhan, BilgeGanzfried, SamSasai, HiroyukiGao, BinSasai, KazutoGao, DavidSasaki, KenGao, HuijunSassone, VladimiroGao, KaizhouSato, HiroyukiGao, LianruSato, ManabuGao, LongxiangSauermann, StefanGao, ShangceSavagatrup, SucholGao, SheshengSavaglio, ClaudioGao, WeiSaval-Calvo, MarceloGao, XiaofengSavazzi, PietroGao, XuefeiSavazzi, StefanoGao, YabiaoSavi, PatriziaGao, YansongSavin, Igor Yu.Gao, YujiSawacha, ZimiGao, ZheSawada, YoheiGao, ZhenzhenSaxena, NavratiGao, ZhiSaxena, NeeteshGaraba, ShunguSayed-Ahmed, Ezzeldin YazeedGarbaras, AndriusSaylam, KutalmisGarces, HugoSberveglieri, VeronicaGarcía Domínguez, Juan JesúsScagliola, MicheleGarcia Vazquez, Juan PabloScaioni, MarcoGarcía, AntonioScalenghe, RiccardoGarcía, CarmeloScampicchio, MatteoGarcía, Carmelo R.Scannapieco, AntonioGarcia, Diana VilelaScannapieco, Antonio FulvioGarcia, JordiScapaticci, RosaGarcía, Juan C.Scaradozzi, DavidGarcía, Mario OrtizScarano, SimonaGarcia, Nuno M.Scarpato, NoemiGarcia, PedroScarpiniti, MicheleGarcía, TaniaScarselli, GennaroGarcia-Ceja, EnriqueSchabowicz, KrzysztofGarcía-Chamizo, Juan ManuelSchaefer, Hans-EckhardtGarcía-Cortés, SilverioSchaffert, NinaGarcía-Cuesta, EstebanSchakel, WouterGarcía-Diego, Fernando-JuanSchall, Mark C.García-Fernández, Ángel F.Schauer, StefanGarcia-Garreton, GonzaloSchauer, ThomasGarcia-Guzman, JavierSchazmann, BenjaminGarcía-Herrero, JesúsScheers, BartGarcía-Iriepa, CristinaSchembra, GiovanniGarcía-Magariño, IvánScheme, ErikGarcía-Massó, XavierSchena, EmilianoGarcia-Melendez, EduardoSchenato, LucaGarcía-Olmo, JuanScheuermann, AlexanderGarcia-Palacios, EmilianoSchiavone, GiuseppeGarcia-Pena, AxelSchiavulli, DomenicoGarcia-Perez, ArturoSchiff­ner, Mar­tinGarcía-Pineda, MiguelSchilt, StephaneGarcía-Rubio, CarlosSchindelhauer, ChristianGarcía-Rupérez, JaimeSchirhagl, RomanaGarcia-Saavedra, AndresSchirrmann, MichaelGarcia-Santos, VicenteSchlachetzki, JohannesGarcia-Segura, SergiSchlaefer, AlexanderGarcia-Silvente, MiguelSchlegel, Joshua P.Garcia-Tobar, JavierSchlegel, ThomasGardel, AlfredoSchmidt, AdamGardel-Vicente, AlfredoSchmidt, JochenGardi, AlessandroSchmidt, MichaelGardiner, BryanSchmidt, Mikołaj KajetanGarg, AkhilSchmihalter, UrsGarg, LalitSchmitt, CorinnaGargiulo, GaetanoSchmitt, KatrinGargiulo, Gaetano DSchmitt, RobertGarmire, ElsaSchmitz, AlexanderGarnero, GabrieleSchmuker, MichaelGartia, ManasSchneider Von Deimling, JensGarzelli, AndreaSchneider, Fabio KurtGarzón Oviedo, Mario AndreiSchneider, HartmutGarzón, José A. CastellanosSchneider, MartinGasbarri, PaoloSchneider, MichaelGąska, AdamSchoefs, FranckGasparini, LeonardoSchoening, TimmGašparović, MateoSchoffelen, Jan-MathijsGastaldi, LauraScholkmann, FelixGasulla, ManuelScholl, Philipp M.Gatteschi, ValentinaScholten, KeeGatto, EmanuelaScholz, MarkusGaudin, ValerieScholze, SebastianGauglitz, GünterSchön, SteffenGaur, GirijaSchöning, MichaelGautier, MatthieuSchönle, PhilippGavet, YannSchormans, JohnGay, MichelSchrag, GabrieleGazzola, EnricoSchudlo, LarissaGazzoni, MarcoSchuetz, MartinGbadebo, AdenowoSchultz, StephenGe, CuiSchulz, FlorianGe, MaorongSchulz, RuthGe, XiaohuaSchulze Lammers, PeterGe, YufengSchumacher, JensGebicki, JacekSchumacher, ThomasGębicki, JacekSchumann, Guy J.-P.Gellini, CristinaSchuster, ThomasGeman, OanaSchütze, AndreasGemmeke, HartmutSchütze, OliverGenest, MarcSchwartz, Andrew J.Geng, ChunhuaSchwartz, David EricGeng, JianghuiSchwarz, BenediktGeng, LinxiaoSchwarz, ChrisGeng, WeidongSchwarz, StefanGenge, BélaSchwarz, Ulrich TheodorGenovese, MarcoSchwendner, JakobGenovesi, SimoneSchwenker, FriedhelmGentile, PiergiorgioSchwieger, VolkerGeorge, LaurentSchwotzer, MatthiasGeorges, MarcŚcibisz, IwonaGeorgiadis, CharalamposScida, KarenGeorgieva, BilianaScioscia, FlorianoGeorgiou, OrestisScirea, MarcoGeradts, ZenoScordamaglia, ValerioGerasimaitė, RūtaScott, Amy M.Gerasimova, Yulia V.Scott, AndreaGerbode, SharonScozzari, AndreaGere, AttilaScully, Christopher G.Geri, AlbertoSebastiano, FabioGerostathopoulos, IliasSebe, FrancescGeršak, GregorSeco Granja, FernandoGeurts, TomasSeco, FernandoGhadimi, HaniehSecu, Elisabeta-CorinaGhafar-Zadeh, EbrahimSedky, MohamedGhanmi, HelmiSedlák, PetrGhannad-Rezaie, MostafaSee, Chan H.Gharakheili Habibi, HassanSeel, ThomasGharanjik, AhmadSeeling, PatrickGhareh Baghi, ArashSeelye, AdrianaGhari Neiat, AzadehSeet, Boon-ChongGhatkesar, Murali KrishnaSegovia-Fernandez, JeronimoGhavidel, AliSeguin, FabriceGhayvat, HemantSegundo, Maurício PamplonaGhazanfari, AmirSegura Garcia, JaumeGhazi, GaliaSeiberl, WolfgangGheorghiu, EugenSeibt, MichaelGheorghiu, MihaelaSeiichi, SudoGheorghiu, Razvan AndreiSeitz, JochenGherlone, MarcoSeitz, W. RudolfGhibaudo, GérardSeixas, AdéritoGhimenti, SilviaSekercioglu, AhmetGhinea, Razvan IonutSekerinski, EmilGhisi, AldoSekiguchi, KazumaGhoraani, BehnazSekretaryova, AlinaGhoreyshi, Seyed MohammadSelci, StefanoGhosh, ArijitSellami, AkremGhosh, BidishaSelvaprabhu, PoongundranGhosh, SumitSelyanchyn, RomanGhysels, MélanieSemanjski, IvanaGia, Tuan NguyenSemkin, VasiliiGiachetti, AndreaSempere-Payá, Víctor MiguelGiacomini, RenatoSendra, SandraGiakos, GeorgeSensinger, JonGialelis, JohnSenthilnath, J.Giannakeas, NikolaosSeo, Dong-HoanGiannakopoulos, TheodorosSeo, HwajeongGiannelli, CarloSeo, JeongHwaGiannetti, AmbraSeo, JunwonGiannetto, MarcoSeo, Min-WoongGiannini, VincenzoSeow, CheekiatGianniou, Michail Sepasgozar, S. M. E.Giansanti, LuisaSepulveda Florez, Martha JohannaGiarre’, LauraSepulveda, AbdonGibaldi, AgostinoSequeira, AnaGiberti, HermesSerafín, VerónicaGidlund, MikaelSeraj, FatjonGierens, KlausSerale, GianlucaGiergiel, MariuszSerban, AdrianaGiernacki, WojciechSergiou, CharalambosGiesko, TomaszSerino, SilviaGigante, Giovanni E.Serio, CarmineGijón Nogueron, GabrielSerodio, CarlosGijsberts, ArjanSerpelloni, MauroGil, BernardSerrano, João Manuel Pereira RamalhoGil, DavidSerrano, Jose JavierGil, EmilioSerrano, LuisGil, Eric De SouzaSerrano, SalvatoreGil, GustavoSerry, MohamedGil, Jorge JuanServières, MyriamGilbert, James M.Servin, ManuelGillanders, Ross NSešek, AleksanderGillham, MichaelSetti, FrancescoGillich, Gilbert-RainerSeufert, MichaelGini, FulvioSevcenco, IoanaGini, GiuseppinaSeveno, RaynaldGinis, PieterSeveri, StefanoGioia, CiroSeyedi, Mir HojjatGioli, BeniaminoSeyyed Monfared Zanjani, JamalGionata, SalviettiSfarra, StefanoGiordan, DanieleSferlazza, AntoninoGiorgetti, AlessioSgobbi, Lívia FlorioGiorgetti, AndreaSgora, AggelikiGiorgio, DianaSha, ChaoGiorgio, IvanShaban, HebaGiosan, IonShafai, CyrusGirau, RobertoShafapour Tehrany, MahyatGirelli, Valentina AlenaShafer, MichaelGiubileo, FilippoShaffer, JamesGiuffrida, Mario ValerioShafiei, ManhazGiuliano, RomeoShafique, MuhammadGiungato, PasqualeShafiullah, GMGiusti, ElisaShah, PratikGizzi, LeonardoShah, PuravGjevestad, Jon GlennShah, TejalGlaser, SebastienShah, Umer HameedGleich, DusanShahbaz, MuhammadGlennie, CraigShahbazi, MozhdehGlisic, BrankoShaheen, SusanGlorieux, EmileShahidi, RezaGłowacki, MichałShahjamali, Mohammad MehdiGlowacz, AdamShahrestani, SeyedGnade, Bruce E.Shahrour, IsamGnatowski, TomaszShaker, Hamid RezaGnyba, MarcinShakev, NikolaGockenbach, ErnstShakthivel, DhayalanGodah, WalyeldeenShalaby, MohamedGodina, RaduShalev, GilGodinez Tello, RichardShamonin, MikhailGodinez, Humberto C.Shamseldin, TamerGodoy, EduardoShan, MaoGoedemé, ToonShang, FangGoel, RahulShang, SonghaoGoenezen, SevanShang, YilunGoes, Luiz Carlos SandovalShang, YuGoga, NicolaeShanmugam, BharanidharanGoh, Yee MeyShao, DangdangGoiffon, VincentShao, JinyouGoit, Jay PrakashShao, LeiGokhale, Vikrant JayantShao, PengGola, ArkadiuszShariati, AliGoldoni, EmanueleSharma, DeeptiGoldstein, MarkusSharma, Piyush SindhuGolen, Erik F.Sharma, Rajnish NGoleva, RossitzaSharma, SanjivGolnabi, Amir H.Sharma, Shree KrishnaGolob, MarjanSharma, VishalGoltz, Douglas M.Shatte, AdrianGomes, DiogoShaw, KirstyGomes, EduardoShayeganrad, GholamrezaGómez Aguilar, José FranciscoShaygan, MehrdadGómez Mancilla, Julio CésarShe, BingGomez, Jose M.She, ZheGómez, LuisSheinker, ArieGomez, María JesusShellaiah, MuthaiahGomez-Dans, JoseShelley, KirkGomez-Lazaro, EmilioShemer, AmirGómez-Pavón, Luz Del CarmenShen, ChongGomez-Romero, JuanShen, DazhongGomez-Ruiz, Jose AntonioShen, JunGonçalves, Luiz M. G.Shen, Jun-HongGonçalves, Paulo Jorge SequeiraShen, QingGonçalves, Rogério SalesShen, YaochunGong, WeiShen, YuanGonzález Briones, AlfonsoShen, YuechengGonzález Cortés, AraceliSheng, BoGonzález De La Rosa, Juan JoséSheng, DuoGonzalez De Los Reyes, Rafael CorsinoSheng, YuboGonzález Herráez, MiguelShepherd, JonathanGonzález Manzano, LorenaSherratt, R. SimonGonzalez Monroy, JavierShestakov, Nikolay. V.González Pérez, IsaíasSheu, Chia-RongGonzález Ruiz, VicenteSheu, Jeng-ShinGonzález, AlejandroSheu, Jeng-TzongGonzález, AmadorSheu, Meng-LiehGonzález, ApolinarShevtsov, SergeyGonzález, Carina SoledadShevtsov, Sergey N.Gonzalez, FernandoShi, BoxinGonzalez, GabrielShi, guangmingGonzález, JesúsShi, HongliangGonzalez, JoaquinShi, HonglingGonzalez, JoseShi, JunboGonzález, Luis CrovettoShi, RonghuaGonzalez, Luis SanchezShi, ShuoGonzález, PascualShi, Wei (Wilbur)Gonzalez, RodrigoShi, XiaowenGonzález-Ayestarán, RafaelShi, ZhiguoGonzález-Camarena, RamónShiau, Jaw-KuenGonzález-Hidalgo, ManuelShieh, Wann-YunGonzalez-Ortega, DavidShields IV, C. WyattGonzález-Zamora, ÁngelShigeta, RyoGonzalo, JesúsShih, Ching-longGoodall, Todd RichardShih, Kuei-PingGoodhew, SteveShih, Po-JenGoorts, PartrikShih, SteveGoossen, KeithShih, Ting-YenGope, ProsantaShih, YiChangGorawski, MichalShikano, YutakaGorcin, AliShikida, MitsuhiroGórecki, TomaszShilov, NikolayGorgutsa, StepanShim, Duk-SunGorretta, NathalieShim, Yoon-BoGórski, FilipShimada, KunioGórski, MarcinShimada, UdaiGorte, BenShimura, TomoyaGosalbez, JorgeShin, Dong JinGosetti, FabioShin, JitaeGosselin, BenoitShin, Jong-DugGostar, Amirali KhodadadianShin, SangwooGosztolya, GáborShin, Su RyonGotta, AlbertoShinbo, KazunariGouaïch, AbdelkaderShinkuma, RyoichiGoud, K YugenderShinmura, FumitoGough, RyanShinoda, KazumaGounaridis, DimitrisShinohara, MasanaoGouttefarde, MarcShirahama, KimiakiGouvêa, Paula M. P.Shiroma, Wayne A.Gouwanda, DarwingShirvanimoghaddam, MahyarGoverni, LapoShivaramaiah, Nagaraj C.Govindan, PramodShivdasani, Mohit NareshGow, JasonShlyagin, MikhailGoyal, SiddharthShmaliy, Yuriy S.Gozali, Alfian AkbarShojafar, MohammadGozdowski, DariuszShokrolah Shirazi, MohammadGoze, ChristineShpacovitch, VictoriaGrabham, Neil J.Shrestha, RajuGrace, PaulShrestha, SudhirGraditi, GiorgioShrirao, AnilGragnani, Gian LuigiShrivastava, AatmeshGraham, ScottShtenber, GiorgiGramaglia, MarcoShtepliuk, IvanGrammalidis, NikosShtrepi, LouenaGranato, DanielSi, LiangGrangeat, PierreSi, WeishengGranica, SebastianSiadat, MaryamGranichin, Oleg N.Sibbett, ScottGrantham, MichaelSibilski, KrzysztofGrassi, SilviaSica, StefaniaGrasso, MarzioSiciliani, MarioGrau, GerdSiddiki, S.M.A. HakimGravina, RaffaeleSidiropoulos, ChristosGray, AndrewSidiropoulos, PanagiotisGray, Michael D.Siebenaler, ShaneGreen, DavidSiegel, JoshuaGreen, ScottSielużycki, CezaryGreener, JesseSierpiński, GrzegorzGregor, IngoSierra-Castaner, ManuelGregor, PetrSierra-Hernández, Juan ManuelGregory, JohnSierra-Rosales, PaulinaGreidanus, HarmSifuentes, E.Greinert, JensSigman, MichaelGrela, JakubSignori, AlessioGrelowska, GrażynaSiirtola, PekkaGrewal, YadveerSik-Lányi, CecíliaGriep, MarkSikora, JanGriffin, JamesSikora-Fernandez, DorotaGriffin, Peter B.Sikorski, WojciechGriggio, FlavioSila-Nowicka, KatarzynaGrigorie, Teodor LucianSilic, MarinGrill, RomanSilva, Agnelo R.Grilo, CarlosSilva, AntónioGrimaldi, DomenicoSilva, Bhagya NathaliGrimaldi, StefaniaSilva, CatarinaGritli, YasserSilva, Gabriela VenturaGrobelnik, MarkoSilva, Hugo MiguelGrochla, KrzysztofSilva, IvanovitchGroen, Thomas A.Silva, José LuísGroenli, Tor MortenSilva, LígiaGrondin, FrancoisSilverston, ThomasGrosch, TheodoreSilwal, AbhiseshGross, JasonSim, Sung-HanGrossi, MarcoSima, ChaotanGrout, Ian A.Simanjuntak, Firman MangasaGroves, RogerSimas Filho, Eduardo F.Groza, BogdanŠimek, JiříGruber, JonasSimeunović, MarkoGruber, ThomasSimian, DanaGrudzien, Colin JamesSimion, Cristian E.Gruev, ViktorSimon Araya, SamuelGruić, IgorSimon, Ann M.Grulkowski, IreneuszSimon, AnnieGruppioni, EmanueleSimon, GyulaGrzechca, DamianSimoncelli, SabrinaGrzeskowiak, MarjorieSimone, AndreaGu, FengshouSims, NeilGu, FuboSingh, DhananjayGu, FuqiangSingh, GautamGu, HaichangSingh, KeshavGu, HuanSingh, Keshav D.Gu, TaoSingh, Kunwar K.Gu, WentingSingh, MadhusudanGu, YanleiSingh, NavragGU, YuandongSingh, RanjanGualandi, IsaccoSingh, Surya P.Guaragnella, CataldoSingha, SubhankarGuariglia, EmanuelSinghal, KunalGuarneri, Giovanni AlfredoSinha, JasmineGuarnieri, Andrea MontiSinharoy, IndranilGuascito, Maria RacheleSinibaldi, AlbertoGuccione, PietroSioris, ChristopherGude, MaikSioshansi, RamteenGudmundsson, Jon TomasŠipoš, MartinGudmundsson, SnorriSips, PatrickGüemes, AlfredoSiqueira Dias, José A.Guerci, JosephSirakov, NikolayGuerra, AnnaSirghi, LucelGuerra, RaúlSisinni, EmilianoGuerra, VictorSitnik, RobertGuerreiro, ArielSivanathan, AparajithanGuerrero, Juan I.Sivaraman, GaneshGuerrero-Castillanos, FermiSivashankar, ShilpaGuerrero-Ibáñez, J. A.Sjödahl, MikaelGuerri, Juan CarlosSkarlatos, DimitriosGuerrieri, AntonioSkeie, Nils-OlavGui, GuanSkilodimou, Hariklia D.Gui, HaichaoSkládal, PetrGuida, DomenicoSklivanitis, GeorgiosGuidetti, RiccardoSkog, IsaacGuidi, FrancescoSkopeliti, AndrianiGuijarro, LuisSkrzypczyński, PiotrGuijt, RosanneSkyllas-Kazacos, MariaGuilbert, DamienSlamanig, DanielGuimaraes, Dayan AdionelSlavič, JankoGuivant, JoseSleewaegen, Jean-MarieGul, RubiSletten, MarkGulati, ShellySlingsby, AidanGulinatti, AngeloSlouka, ChristophGulizzi, VincenzoSmall, AlexGuminski, RobertSmall, DavidGuna, JožeSmarsly, KayGunawardana, UpulSmirnov, AlexanderGünther, ManuelSmith, Eric P.Guo, DejunSmith, StewartGuo, FuchunSmith, StuartGuo, HongzhiSmith, YorkGuo, JianlinSmolka, BogdanGuo, JimingSmulko, JanuszGuo, QingboSmyl, DannyGuo, ShuangzhuangSnoeys, WalterGuo, Tai L.So, HongyunGuo, TingSo, JungminGuo, WenzhongSoangra, RahulGuo, XinfeiSoares Júnior, AmílcarGuo, YangmingSoares, Vasco N.G.J.Guo, ZhouSobaszek, MichałGupta, AshishSobolev, Nikolai AndreevitchGupta, InderSobral, Andrews CordolinoGupta, RishabhSobrino, Xosé Antón VilaGurov, IgorSocoró, Joan ClaudiGursoy, DogaSodhro, Ali HassanGustavsson, TorbjörnSodnik, JakaGu-Stoppel, ShanshanSoekadar, SurjoGuterman, HugoSöffker, DirkGutiérrez García, Miguel ÁngelSoh, Jun HuiGutierrez, Carlos A.Soheilian, BahmanGutiérrez, Eduardo QuevedoSohgawa, MasayukiGutierrez, HectorSohn, Hong-GyooGutiérrez, JonSohn, Jung WooGutiérrez, Juan ManuelSo-In, ChakchaiGutiérrez-Capitán, ManuelSoin, NavneetGutierrez-Heredia, GerardoSöken, Halil ErsinGutjahr, KarlheinzSokolova, NadezdaGuzek, DominikaSola, DanielGuzman, MarceloSolano-Correa, Yady TatianaGuzmán, PaulaSolar, HectorGuzman-Chavez, Ana DinoraSoleimani, VahidGuzman-Quesada, JoseSoler Aznar, MariaGuzman-Sepulveda, Jose RafaelSolimene, RaffaeleGuzzi, JérômeSolla, MercedesGwak, JeonghwanSolmaz, GuerkanGyongy, IstvanSoltani, Mohsen N.Gyrard, AmelieSoltani, PayamGyurcsányi, RobertSołtysik-Piorunkiewicz, AnnaHa Lee, ByeongSoman, RohanHa, IlkyuSommer, MartinHaack, BarrySomov, YevgenyHaak, HarmSon, ByungrakHaas, RüdigerSon, Chang-HwanHäberlin, AndreasSon, DongheeHaberman, MichaelSon, Hyoung IlHabib, AnowarulSon, JunggabHabib, Md SelimSon, YunsikHabisreuther, TobiasSong, Byung CheolHachaj, TomaszSong, CaixiaHaddad, Diego BarretoSong, ChaoyunHaddad, YoramSong, EdwardHaddadi, KamelSong, GangbingHadjicostis, ChristoforosSong, HeechunHadjimitsis, DiofantosSong, HoubingHaesler, SebastianSong, MinkyuHafiz, Md Abdullah AlSong, YinchenHafuka, AkiraSong, YongzeHagedoorn, Peter-LeonSong, ZhanHagelauer, AmelieSonntag, Matthew D.Hagen, GunterSonobe, ReiHaghparast, MajidSopanen, JussiHagner-Derengowska, MagdalenaSophocleous, MariosHahaj, TomaszSoria, SilviaHaider, GolamSoriano, AntonioHaider, Mohammad FaisalSorici, AlexandruHaigh, Paul AnthonySorli, BriceHaijun, ZhangSorli, MassimoHaindl, RichardSorrichetta, AlessandroHaitjema, HanSośnica, KrzysztofHajder, LeventeSotelo Monge, Marco AntonioHajduch, GuillaumeSotner, RomanHájek, PetrSoto, RobertHaji Maghsoudi, OmidSouihi, SamiHajializadeh, DonyaSoundappan, ThiagarajanHajimirsadeghi, MohammadSousa Pavani, GustavoHajnal, ZoltánSousa, Armando JorgeHakala, IsmoSousa, InêsHakem, NadirSousa, JoaquimHakonen, AronSouza, FranciscoHakuta, KohzoSouza, Richard DemoHaladjian, JuanSovják, RadoslavHalder, ArnabSpachos, PetrosHall, Andrew J.Spagnolo, VincenzoHall, Carlos AlbertoSpanakis, Emmanouil G.Hall, Neal ASpanò, AntoniaHallaji, MiladSparavigna, Amelia CarolinaHallil, HamidaSparks, ErinHam, SuyunSpearrin, MitchellHamacher, DennisSpecogna, RubenHamad-Schifferli, KimberliSpencer, Billie FHamedi, AmirmasoudSpencer, BruceHamilton, TaraSpencer, EdmundHammoudeh, MohammadSperanza, DomenicoHampp, NorbertSperlì, GiancarloHamza, BenSperling, BrentHan, BingSpezzi, LoredanaHan, ChongzhaoSpicer, ChristopherHan, Dong SeogSpigulis, JanisHan, FengtianSpinazze, AndreaHan, GunheeSpinsante, SusannaHan, Jen-YuSpivak, ArthurHan, JungheeSpizzichino, ValeriaHan, JungongSportelli, Maria ChiaraHan, MingSpringer, ShmuelHan, SangUkSpüler, MartinHan, Shi-yuanSpyridopoulos, TheodorosHan, ShuaiSreekanth, Kandammathe ValiyaveeduHan, Soo DeokSresht, VishnuHan, WenlinSrinivasan, AvinashHan, YoukyungSrinivasan, KathiravanHan, Young-GeunSriram, RashmiHancock, Craig MatthewSrivastava, Yogesh KumarHandkiewicz, AndrzejSroka, RyszardHanke, UlrikStahl, FrankHansen, ClintStahl, UllrichHansen, JohnStaiano, AntoninoHansen, Mark FStajanca, PavolHao, GuangboStamate, EugenHao, ZhaoStamatopoulos, Constantine A.Haque, A.N.M.M.Stamos, KostasHaque, RezwanulStan, GeorgeHaque, Rubaiyet IftekharulStanacevic, MilutinHara, ShinsukeStancic, IvoHare, KimStančin, SaraHarezlak, KatarzynaStanic, SamoHarguess, JoshStanker, Larry HenryHarik, El Houssein ChouaibStankiewicz, OlgierdHariri, Hassan HusseinStankovski, VladoHarizanov, StanislavStansfield, BenHärmä, HarriStarecki, TomaszHarman, StephenStark, NinaHarms, JanStaruch, MargoHarnois, MaximeStateczny, AndrzejHarren, Frans J.M.Stateczny, Andrzej EugeniuszHarris, PaulStathopoulos, NikolaosHarris, WilliamStatkiewicz-Barabach, GabrielaHartigh, Ruud J.R. DenStatsyuk, Natalia V.Hartke, BerndStavenga, DoekeleHartl, Darren J.Stavn, Robert H.Haruki, MitsuruStavness, IanHarvey, RichardStavrakis, StavrosHasan, SouleimanStavropoulos, PanagiotisHasegawa, HideyukiStavropoulos, Thanos G.Hashemi Beni, LeilaStavroulakis, Georgios E.Hashemi, MortezaSt-Charles, Pierre-LucHasni, HasseneSteele, John P HHassan, FirasSteele, Vaughn R.Hassan, ModarSteen Redeker, ErikHassanalian, MostafaSteenhaut, KrisHassanin, HanyStefanescu, RazvanHassui, AmauriStefani, AlessioHatzfeld, ChristianStefania, MonicaHaubelt, ChristianStefano, PaoloniHauk, OlafStefanov, AndrejHausman, SławomirStefanov, KonstantinHaviar, StanislavSteidl, StefanHavlík, JanStein, Manuel S.Hawash, ZaferStepanov, Oleg A.Haworth, JamesStephane, CaroHayajneh, ThaierStepien, KrzysztofHayat, AkhtarŠter, BrankoHayat, MunawarSterle, OskarHe, ChuSterling, DavidHe, FangningStetter, RalfHe, HuijingStevens-Navarro, EnriqueHe, JiayuanStewart, BrianHe, Jing SelenaStewart, RebeccaHe, KaijianSthal, FabriceHe, LiStiharu, IonHe, LimingStilli, AgostinoHe, LongStinco, PietroHe, LuxiaoStine, Keith J.He, QiangStipanicev, DarkoHe, SuiningStirling, LeiaHe, XianqiangStiros, Stathis C.He, XiaoyuanStoffelen, AdHe, YanStoikov, Ivan IvanovichHe, ZhihaiStoimenov, LeonidHeadley, William ChristopherStojak Repa, KristenHeberger, KarolyStolarczyk, AgnieszkaHéberger, KárolyŠtolc, SvoradHebrard, LucStopforth, RiaanHedegaard, BrockStoppa, DavidHeemstra De Groot, SoniaStoppini, AurelioHeeren, DerekStorace, MarcoHefferman, GeraldStork, WilhelmHefnawi, MostafaStornelli, VincenzoHegde, NagarajStortini, Angela MariaHeidari, HadiStrait, Megan K.Heijnen, Petra W.Straub, JeremyHeikkinen, Jani ErikStreitferdt, DetlefHeikkonen, JukkaStrelniker, Yakov M.Heilbronner, UrsStreltsov, Anatoly V.Hein, BjörnStriccoli, DomenicoHein, JoachimStrle, DragoHeise, RainerStrle, GregorHeiselberg, HenningStrohmeier, MartinHelmus, Jurjen RienkŠtroner, MartinHelseth, LarsStroulia, EleniHemmati, FarzadŠtruc, VitomirHenderson, RobertStruk, PrzemyslawHenesey, LawrenceStrumillo, PawelHeng, LionelStrzelecki, MichalHenley, RobertStuart, SamHenriques, Renato FilipeŠtumberger, GorazdHensel, StefanSu, BinHeo, JinseokSu, Bo-chiuanHeo, MoonbeomSu, Ching-liangHer, Shiuh ChuanSu, DanHeras, Dora BlancoSu, DaobiligeHerkommer, AloisSu, JingHernandez Bennetts, VictorSu, JudithHernández Creus, AlbertoSu, JunweiHernández Sosa, José DanielSu, Ming-ShouHernández, ÁngelaSu, Pi-GueyHernández, DavidSu, RongHernández, David NaranjoSu, RuoyuHernandez, Jesus C.Su, Tung-ChingHernandez, LuisSu, YanjunHernandez, MonicaSu, YiHernández, NoeliaSu, YishanHernández-Ramos, José LuisSu, ZhijuanHernando, JordiSua, Yong MengHernando, JorgeSuarez, AlvaroHernando, MiguelSuárez-Albela, ManuelHerráez, JoséSubasi, AbdulhamitHerráez-Hernández, RosaSubramani, SivaHerraiz, Joaquín LópezSubramani, ThiyaguHerrera, ManuelSubramaniyan, ManivannanHerrera-May, AgustínSuciu, GeorgeHerrero-Agustín, José-LuisSudhir, VivishekHerrmann, IttaiSuematsu, KoichiHerrmann, J. MichaelSuen, Jacky Y.Herrnsdorf, JohannesSugano, MasashiHervás, RamónSuh, Doug YoungHerzog, Joseph B.Suh, In-SooHerzog, OttheinSuh, Myoung-GyunHespel, LaurentSuh, Young SooHess, LauraSuhr, J.K.Hesselbarth, AnjaSui, YaoHeuer, HenningSujecki, SlawomirHeuss-Aßbichler, SorayaSuk, TomášHewson, MichaelSukhov, SergeyHeyd, RodolpheŠuligoj, FilipHeydari, ShahramSullivan, Rani W.Hianik, TiborSułowicz, MaciejHibino, HiroshiSumets, MaximHickey, Stephen G.Summerscales, JohnHicks, YuliaSun, ChenHidalgo López, José AntonioSun, ChenhuHieyong, JeongSun, Chung-HsunHilkert, James M. (Jim)Sun, DaliHill, ChrisSun, FenggangHilloulin, BenoitSun, GongchenHinkov, BorislavSun, GuangcaiHinks, JamieSun, GuanghaoHirofumi, NogamiSun, GuodongHiromori, AkihitoSun, HuaHiroshi, IshidaSun, IwenHirsch, ThomasSun, JianHirsikko, Anne EmiliaSun, JiapengHirtle, Stephen C.Sun, JiguangHiryu, ShizukoSun, JingtaoHitchens, MichaelSun, JinpingHitz, MartinSun, KangHively, DeanSun, KeXunHjelme, Dag RoarSun, KeyeHlubina, PetrSun, MengHo, Chian C.Sun, MinhongHo, DeanSun, PengHo, Jun-WonSun, RuopengHo, SiuchunSun, RuoyuHo, Tan-JanSun, ShangpengHoblos, GhalebSun, ShuliHochreiner, ArminSun, WeifengHoebeke, JeroenSun, WeiweiHoegner, LudwigSun, Yung-ShinHoelzl, GeroldSun, Yun-LuHoffman, LuisSun, YuqiongHoffmann, AlwinSun, YuxiangHoffmann, AndreasSundaram, SureshHoffmann, DanielSundararajan, AnaniahHoffmann, Markus M.Sung, Chang KyungHoffmann, StefanSung, Gun YongHolade, YaoviSung, Guo-mingHolak, KrzysztoSunny, S. M. Nahian AlHölbl, MarkoSuppa, AntonioHolder, LawrenceSuprun, ElenaHole, Lars R.Suraneni, PrannoyHolgado-Terriza, Juan AntonioSuresh, Mahima AgumbeHoller, StephenSuria, AteeqHolmes, ChristopherSurl, LukeHolmgren, AndrewSurmick, DavidHolst, AndersSurowka, ArturHolst, ChristophSusmel, SabinaHolzinger, AndreasSutherland, KennethHomayouni, SaeidSutin, AlexanderHommes, AlexanderSütő, JózsefHomsy, AlexandraSutton, GordonHonda, KatsuhiroSuvorova, SofiaHondongwa, DonaldSuzuki, KenichiroHoneine, Jean-LouisSuzuki, NaoyaHoneine, PaulSuzuki, ReijiHong, Chang-KiŠvaco, MarkoHong, Cheng-YuŠvanda, MilanHong, DaesikSvecko, RajkoHong, JaesungSvogor, IvanHong, JooyoungSwami, NathanHong, Jung-HongSweetman, MartinHong, Suk MinSwensen, John P.Hong, Wei-ChiangŚwiderski, WaldemarHong, Youn-SikŚwit, GrzegorzHongkun, LiSybis, MichalHopp, TorstenSyed-Abdul, ShabbirHoppe, MatthiasSykioti, OlgaHoque, SanaulSynnott, JonathanHorabik, JózefSyrovy, TomasHorak, EmaSysoev, VictorHoremuz, MilanSzabo, LorandHorla, DariuszSzabó, SzilárdHorne, MalcolmSzabo, Thomas L.Horng, Gwo-JiunSzczęsna, AgnieszkaHorng, Tzyy-ShengSzczęsny, SzymonHorrillo Güemes, CarmenSzczuko, PiotrHorsley, AndrewSzedlak-Stinean, Alexandra-IuliaHortal Quesada, EnriqueSzékács, InnaHorvat, BojanaSzekely, GyorgyHorváth, JuditSzewczyk, RomanHorvath, RobertSziebig, GaborHorvath, TomasSzirányi, TamásHoshino, YoheiSzkaliczki, TiborHoshyarmanesh, HamidrezaSzkulmowski, MaciejHosoya, NaokiSzpytko, JanuszHossain, M. ShamimSzulczyński, BartoszHossain, MahmudSzulwic, JakubHossain, Md. DelwarSzweda, RozaHosseinzadeh, MehdiSzwoch, MariuszHossny, MohammedSzwoch, WioletaHotovy, IvanSzymborski, TomaszHou, Kun-meanSzymczyk, PiotrHou, LeiTabatabaei, NimaHou, LianpingTabb, AmyHou, ShuangTabib-Azar, MassoodHou, YunfeiTaborri, JuriHouben, SebastianTadepalli, SirimuvvaHoward, IanTadokoro, YukihiroHowcroft, JenniferTaebi, AmirtahàHowes, PhilipTaffese, Woubishet ZewduHowitt, Crispin A.Taghizadeh, OmidHoxley, DavidTaghvaeeyan, SaberHożyń, StanisławTahara, HironobuHrad, JaromirTahavori, FatemehHrishikeshavan, VikramTaheri-Tehrani, ParsaHristoforou, EvangelosTaherizadeh, SalmanHsia, ShaoYiTakahashi, YasutakeHsiao, Chun-ChingTakamasu, KiyoshiHsiao, Fu-yuenTakashima, KazutoHsiao, Hsu-ChunTakebayashi, HidekiHsiao, Ta-ChihTakeda, RyoHsieh, Cheng-HsiungTakeda, YasunoriHsieh, Hsun-PingTakemura, HiroshiHsieh, Hung-LinTakemura, KenjiroHsieh, Hung-YunTalaat, AhmedHsieh, KuangwenTalavera, EdgarHsu, Feng ChiTalbi, AbdelkrimHsu, Heng-TungTaleat, ZahraHsu, Li-taTaleb, A.Hsu, Shih-HsiangTalebifard, PeymanHsu, Ting-YuTalla, VamsiHsu, Wei-ChunTamagnone, MarioHsu, Wei-FengTamás, LeventeHsu, Yu-LiangTamberg, GertHu, Ai-PingTamer, AykutHu, ChuanTamil, LakshmanHu, DaodaoTammaro, UmbertoHu, Fang Q.Tamošiūnas, VincasHu, FengTamura, HirokiHu, JianjunTamura, TomonoriHu, JiankunTan, BingfengHu, JunTan, BoHu, JunhuiTAN, Chin YawHu, MiaoTan, Chun KiatHu, NingTan, HaowenHu, QijunTan, Hwee-PinkHu, QingwuTan, JeanneHu, ShaTan, LiHu, ShaohanTan, LianshengHu, ShuowenTan, NingHu, SimonTan, QiulinHu, WeiduoTan, SayHu, WeifeiTan, ShurunHu, XiaogongTan, ThiamteckHu, XiaosongTan, Yew TeckHu, YihuaTanaka, MasayukiHu, YingjieTanaka, YoHu, Yuh-ChungTanbour, Emad Y.Hu, ZhaozhengTang, BinHu, ZhongxuTang, DianpingHuan, XunTang, HongHuang, Cheng-LiangTang, LinHuang, Cheng-ShengTang, LiqunHuang, Chih ChungTang, Lu-AnHuang, Chih-ChingTang, SuhuaHuang, Chih-HsienTang, WenwuHuang, Chih-WeiTang, XinhuaHuang, Chih-YuanTang, YuxingHuang, Ching-JerTang, ZhichuanHuang, Chi-YenTani, HirofumiHuang, Chung-ChiTani, JacopoHuang, DiTaniguchi, Rin-ichiroHuang, Genin GaryTanner, EdenHuang, Guang-BinTao, KaiHuang, GuangyanTao, MingHuang, HaiyanTao, XinHuang, HaoTao, XuyuanHuang, Helen J.Tapetado, AlbertoHuang, HuaweiTapete, DeodatoHuang, JennyTappia, Paramjit S.Huang, JianxiTarabini, MarcoHuang, JiaruiTaramelli, AndreaHuang, Ji-JerTarantino, CristinaHuang, JingfengTarantino, LauraHuang, JingyiTaravat, AlirezaHuang, KejunTărniceriu, DanielaHuang, LimingTashev, IvanHuang, Nien-TsuTassi, AndreaHuang, Pei-ChiTassou, ChrysoulaHuang, Qing-AnTatar, ErdincHuang, QinghuaTate, Rothwelle J.Huang, QinlongTatlas, Nicolas-AlexanderHuang, Shih-ChangTatullo, MarcoHuang, Shih-ChiaTauro, FlaviaHuang, Shiuh-JerTavakoli, MahmoudHuang, SunanTavakolian, KouhyarHuang, WeiminTavares, João Manuel R. S.Huang, WenliTavassolian, NegarHuang, XianTavernier, FilipHuang, XiaodongTayler, MichaelHuang, XinboTedjini, SmailHuang, YanboTeixeira, Adunias Dos SantosHuang, YanjunTeixeira, Fernando A.Huang, YingTeixeira, Fernando L.Huang, YishuoTeixeira, Marcos F.S.Huang, YuTejeda-Lorente, AlvaroHuang, YunTemiz, YukselHuang, Zi-GuiTemko, AndriyHuck, AlexanderTenbohlen, StefanHudec, RobertTengfei, ChangHudson, JenniferTennina, StefanoHue, OlivierTenreiro Machado, J. A.Huertas, César SánchezTeo, Chek SingHuertas, GloriaTeo, Rodney Swee HuatHughes, IfanTeo, Soo KngHughes-Riley, TheodoreTeodorescu, FlorinaHuh, JungwonTeranishi, NobukazuHuh, Jun-HoTerebes, RomulusHuh, Yun SukTerroso-Saenz, FernandoHui, MaTervonen, Jouni K.Hui, xieTesei, AlessandraHulsey, LeroyTessarolo, MartaHung, Ho-LungTestoni, NicolaHung, Jui-chungTeti, RobertoHung, Kuo-YungTetienne, Jean-PhilippeHung, Ming-ChihTeunissen, PeterHunyadi, BorbálaTeuvo, SuntioHuo, Fang-JunTeymourian, HazhirHuo, LinshengThalhammer, GregorHuotari, MattiThamaraparambil Jayaram, DhanyaHusanu, ElenaTharmarasa, RatnasinghamHussain, Abir JaafarTheiler, JamesHussain, AmjadTheodorakopoulos, IliasHussain, S.M. SuhailTheodoridis, EvangelosHussain, TanveerTheodoropoulos, DimitrisHuston, DryverThibault, SimonHuttunen, HeikkiThiel, MichaelHutu, Florin DoruThiele, TobiasHuynh, Thanh-CanhThielemann, ChristianeHwang, Chi-HungThielens, ArnoHwang, Chi-SunThiery, YannickHwang, Dong-HwanThilakarathna, KanchanaHwang, Han-JeongThom, ChristianHwang, Keum CheolThomas, DiegoHwang, Paul A.Thomas, Jean BaptisteHwang, Ruey-BingThomas, Peter JHwang, Seong OunThomas, Stewart J.Hwang, Suk-WonThombre, SarangHwang, TzonelihThompson, AlexHwang, WonjunThompson, DaveHwang, YonghaThomsen, Erik VilainHyakawa, YuichiThramboulidis, KleanthisHyakutake, TsuyoshiThummala, PrasanthHyde, ChristianTiakas, EleftheriosHytönen, VesaTian, FengchunHyun, Seung-HoTian, Gui YunIacomi, FeliciaTian, HeIacomussi, PaolaTian, JiandongIacovacci, VeronicaTian, JieIakovidis, DimitrisTian, LimeiIandolo, BeniaminoTian, ZengshanIannazzo, DanielaTian, ZhenhuaIanoul, A.I.Tian, ZongshunIbaraki, SoichiTic, VitoIbaraki, YasuomiTiemann, MichaelIbarlucea, BergoiTing Goh, ShuIbarra-Arenado, ManuelTiti, HaniIbarra-Castanedo, ClementeTiu, Brylee DavidIbuki, TatsuyaTivive, Fok Hing ChiIdiano, D’AdamoTiwari, Kumar AnubhavIdzkowski, AdamTixier-Mita, AgnesIeng, SiohoiTjahjowidodo, TegoehIera, AntonioTjin, Swee ChuanIervolino, PasqualeTkáč, JánIgasaki, TomohikoTlelo-Cuautle, EstebanIglesias, Carlos BorregoTo, PeterIglesias, FélixTodorov, YankoIglesias, MiguelTodri-Sanial, AidaIgoe, DamienTognetti, AlessandroIhantola, SakariToivonen, JuhaIida, DaisukeTokuda, TakashiIizuka, KotaroToledo, Claudio Fabiano MottaIizuka, MakitoTolentino, MatthewIjaz, Muhammad FazalTolosa, EzequielIkeda, AkihikoToma, Daniel MihaiIkeda, MakotoToma, KojiIkmo, ParkToma, ManaIkonomidou, Vasiliki N.Tomai, EleniIliev, IvoTomar, Vikrant SinghIlioudis, Christos V.Tomarchio, OrazioIliyasu, Abdullah M.Tomas-Burguera, MiquelIm, HyungsoonTomašić, IvanImai, NiltonTomasiello, StefaniaImamura, GakuTomasz, RymarczykImasu, RyoichiTomaszewski, DariuszImbiriba, TalesTombelli, SaraImboden, MatthiasTomer, Vijay K.Impedovo, DonatoTomic, IvanaImperatore, PasqualeTomic, SlavisaImperatore, StefaniaTomisa, TomislavInagaki, TetsuyaTomita, ShunsukeInci, FatihTommaselli, Antonio M. G.Ino, KosukeTonacci, AlessandroInoue, TakuyaTondolo, FrancescoIoakimidis, ChristosTong, WeitianIoannidis, CharalabosTonkin, EmmaIoannidis, DimosthenisTonoli, AndreaIodice, AntonioToral Marín, SergioIommelli, SalvatoreTorbick, NathanIonescu, BogdanTöreyin, HakanIonete, Eusebiu IlarianTorfs, TomIonita, SilviuTorres, LizethIqbal, Hafiz M.N.Torres, PedroIraci, NunzioTorres, Pedro M. B.Irannezhad, MasoudTorres-Cisneros, MiguelIribarne, LuisTorres-Gomez, IsmaelIrukuvarghula, SandeepTorres-Moren, Jose-LuisIsaac, KakkattukuzhyTorres-Roman, Deni LibradoIsaacs, Jason T.Torres-Sanchez, JorgeIsakov, DmitryTorres-Sospedra, JoaquínIshaq, IsamTorti, EmanueleIshigami, GenyaTortia, CristinaIshii, KenichiTortini, RiccardoIshikawa, DaitaroTortonesi, MauroIshikawa, JunTortschanoff, AndreasIshikawa-Nagai, ShigemiTorun, HamdiIshmanov, FarruhTorunbalci, Mustafa MertIslam, Md. MonirulTosato, PietroIslam, S.M. RiazulTosh, Deepak K.Islam, SalekulTosi, DanieleIslam, Shama NazTóth, TündeIsmagilov, Flur R.Toubal, LotfiIsola, GaetanoTourani, RezaIson, DavidTowsyfya, HosseinIsrael, MartinToxiri, StefanoIstrate, DanTragos, Elias Z.István, BókkonTrakadas, PanagiotisIto, SeigoTramarin, FedericoIto, TakeshiTrammell, Scott A.Itoh, TamitakeTran, Binh Q.Itoyama, KatsutoshiTran, Gia KhanhItuero, PabloTran, HuyIturrate, IñakiTrashin, StanislavIvanov, Ilia N.Traver, VicenteIvanov, RadoslavTraverso, FedericoIvanov, RosenTrebar, MiraIvanov, StepanTrejo, LuisIvashov, SergeyTremeau, AlainIvorra, EugenioTreuillet, SylvieIwami, KentaroTrevathan, JarrodIwasaki, AkiraTriantafyllidis, AndreasIyota, TaketoshiTrichopoulos, GeorgiosIzadyar, AnahitaTrihinas, DemetrisIzquierdo, AlbertoTrilles, SergiIzquierdo, Estibaliz MartinezTrinder, JohnIzquierdo, Miguel Ángel GarcíaTripathy, NirmalyaIzu, NoriyaTripunitara, MaheshIzumi, ShintaroTrögl, JosefIzydorczyk, JacekTroglia Gamba, MicaelaJ. G. Teunissen, PeterTroiani, EnricoJaakkola, AnttiTroisi, SalvatoreJabari, ShabnamTrommer, GertJackson, NathanTrommer, Gert F.Jacob, EduardoTroncone, AntonelloJacob, RomainTrongnhan, LeJacobs, PeterTronin, A. AndrewJacobsen, Knut StanleyTrotta, AngeloJácome, CristinaTrovati, MarcelloJacquir, SabirTrung, Tran QuangJacquot, MaximeTruong Hong, LinhJafari, RahelehTruong, LongJafari, SoheilTruong, NguyenJaffrezic-Renault, NicoleTruong-Hong, LinhJaganathan, ArunTrusiak, MaciejJágerská, JanaTrutoiu, LauraJahn, MarcoTsai, Chi-YiJaitner, ThomasTsai, Chun-MingJakieła, SławomirTsai, Kang-TingJakóbczak, Dariusz JacekTsai, Meng-HsunJakovels, DainisTsai, Ming-FongJakšić, OlgaTsai, Pei-WeiJaksic, VesnaTsai, Pei-YunJakubik, Wiesław P.Tsai, Tsuey-linJakubowska, MałgorzataTsai, Wan-YuJalal, AhmadTsai, Yao-HongJamin, ChristopheTsai, Yuh-ShowJamone, LorenzoTsai, Yu-TingJan, Shau-ShiunTsaklis, PanagiotisJanaideh, Mohammad AlTsang, Kim FungJanas, DawidTsangouri, EleniJang, Ho WonTsao, Hen-WaiJang, Jae-WonTsao, YuJang, Jiun-HueiTsay, CHien-YieJang, Kyung-InTseles, DimitriosJang, Seung-YupTseng, Ching-ShiowJang, SoohwanTseng, Chwan-LuJangal, FlorentTseng, Shu-MingJanioud, PierreTseng, Victor Farm-GuooJanjic, JovanaTseng, Wei-LungJankowski, ŁukaszTserepi, AngelikiJankowski, MichałTsigaridas, Georgios N.Jankowski-Mihułowicz, PiotrTsiigkaridis, John (Ioannis)Janota, AlešTsinos, ChristosJansen, BartTsiropoulou, Eirini EleniJansen, Jacob WillemTsoulis, DimitriosJansen, Robert W.Tsow, Francis (Tsing)Jansson, Jussi-PekkaTsukada, ManabuJanting, JakobTsuru, Takeshi GoJantunen, ErkkiTsyganova, Julia V.Janusas, GiedriusTU, ChengJara, AntonioTu, RuiJarchi, DelaramTuci, ElioJarolmasjed, SanazTudorache, FlorinJaromin-Glen, KatarzynaTullo, EmanuelaJaroszewicz, LeszekTumarkin, Andrey V.Jasielec, Jerzy J.Tung Chong, David WongJasinski, GrzegorzTung, Ryan C.Jasiul, BartoszTura, AndreaJaskolka, JasonTurchetta, RenatoJato-Espino, DanielTurchin, IlyaJauregui, Juan CarlosTurel, MatejkaJáuregui-Vázquez, DanielTurini, GiuseppeJavaid, Ahmad Y.Tuzson, BelaJavali, ChitraTwu, Ruey-ChingJavanmard, MehdiTyystjärvi, EsaJaw, Jen-JerTzallas, AlexandrosJayaraman, ChandrasekaranTzemanaki, AntoniaJechow, AndreasUbeda, AndresJędrzejewska-Szczerska, MałgorzataUbertini, FilippoJedrzejewski, Kazimierz PiotrUccheddu, FrancescaJeerapan, ItthiponUchaikin, SergeyJeff, ChilesUchikoba, FumioJekeli, ChristopherUchiyama, KenjiJekova, IrenaUcuncu, MuhammedJemrić, TomislavUddin Ahmed, MobyenJen, Chun-PingUddin, Mohammad SalahJeng, Jen TzongUdrea, BogdanJeng, YihUeberschär, OlafJenkins, KennethUeda, TaroJenkins, R. BrianUehara, NobuoJensen, Brian D.Uezato, TatsumiJeon, GwanggilUgail, HassanJeon, Ju-WonUgbolue, UkadikeJeon, MansikUhl, AndreasJeon, SeokwooUhlemann, SebastianJeon, Yong-HoUjihara, MasakiJeong, Chang KyuUlapane, NalikaJeong, Gu-minUllah, AMM SharifJeong, Han-YouUllah, IhsanJeong, Hee-JinUllo, SilviaJeong, Hoon EuiUllrich, TorstenJeong, Jong SeobUlman, VladimírJeong, YaesukUlrich, MarkusJeong, YoonchanUltes, StefanJesenik, MarkoUmbaugh, ScottJesionek, MarcinUme, I. CharlesJesson, DavidUmek, AntonJevtic, RuzicaUnderhill, Peter RossJezierski, JanUngurean, IoanJha, MithileshUngureanu, FlorinaJi, HaoUrai, MinoruJi, JinchenUrasinska-Wojcik, BarbaraJi, XiaoyuUrban, Matthew W.Ji, YonghoonUrbieta, AitorJi, ZhanlinUrgese, GianvitoJia, BoruUriarte, Rafael BrundoJia, JiabinUrquiza Aguiar, LuisJia, KunUrricelqui, JavierJia, PengUrsu, IoanJia, XiaotingUrsutiu, DoruJia, YanweiUruba, VáclavJia, YuUrzaiz, GabrielJian, FujiUshida, ShunJiang, ChunxiaoUslaender, ThomasJiang, DaiUslar, MathiasJiang, FanUtkin, AndreiJiang, HaoUttam, ShikharJiang, JingUysal, FarukJiang, JunkeUysal, IsmailJiang, MingjunUzel, SebastienJiang, QiVachali, PreejithJiang, RuinianVaculovičová, MarketaJiang, SongVaezi, MojtabaJiang, XiaoningVagionas, ChristosJiang, XinVahabi, HenriJiang, XuchuanVaiana, RosolinoJiao, PengchengVaidya, BinodJiao, YuboVainer, Boris G.Jiawen, JianVaisocherová - Lísalová, HanaJimenez Angeles, LuisVakhitov, AlexanderJimenez, CeciliaValença, JónatasJimenez, FelipeValenta, VáclavJimenez, LuisValente, AntonioJimenez, RicardoValentini, FedericaJiménez-Herranz, José MiguelValentino, MarioJiménez-Martínez, RicardoValenzuela, HectorJin, HaimingValera, EnriqueJin, HongxiaoValero, Eva M.Jin, HuValero-Lara, PedroJin, KyongValiente, DavidJin, Kyong HwanValigi, Maria CristinaJin, MiaoValis, DavidJin, Ren ChengVallan, AlbertoJin, XiVallati, CarloJin, ZhanpengVallet, BrunoJin, ZhigangVallicrosa, GuillemJin, ZongwenValsesia, AndreaJing, QingshenValsesia, DiegoJing, TaoValtierra-Rodriguez, MartinJiralerspong, TrongmunVamanan, BalajeeJiří, ŠebestaVan Bennekom, W.P.Jirka, SimonVan Der Eerden, FritsJobbágy, JánVan Der Loos, HendrikJoerger, MathieuVan Der Pol, EdwinJohansson, Adam JohannesVan Der Sar, ToenoJohansson, ChristerVan Der Veen, Alle-JanJohnson, Blake N.Van Gemert, Jan C.Johnson, Brian AlanVan Gerven, P.W.M.Johnson, MichaelVan Hamme, DavidJohnson, ThomasVan Helleputte, NickJohnston, Steven J.Van Hoof, ChrisJolfaei, AlirezaVan Hulle, MarcJolly, PawanVan Laerhoven, KristofJonas, StephanVan Leeuwen, MartinJonassen, MariusVan Nguyen, BinhJones, ErickVan Schooten, Kimberley S.Jones, J CliffVan Schyndel, RonJones, John WVan Torre, PatrickJong, Gwo-jiaVan Vliet, MarijnJonmohamadi, YaqubVan Winden, StevenJonsdottir, JohannaVandecasteele, KaatJonsson, MagnusVandooren, JenniferJorge, HiginioVanfleteren, JanJosé, RuiVangelista, LorenzoJoseph, WoutVanhille, ChristianJoshi, AnshulVanrumste, BartJoshi, HemduttVanus, JanJoshi, NiravkumarVardasca, RicardoJoshi, SiddharthVardoulis, OrestisJoshi, TanmayaVarga, PalJouan, Pierre-YvesVargas, JavierJoung, Hyou-ArmVargas-Rodriguez, EverardoJourdan, GuillaumeVargas-Rosales, CesarJovanović, DraganaVarghese Chacko, JenuJovic, MilicaVarghese, BlessonJózefczak, ArkadiuszVarghese, JobinJóźwiak-Niedźwiedzka, DariaVargiu, EloisaJu, HeongkyuVarona, PabloJu, HuangxianVarray, FrancoisJu, XiangyangVarvarigou, Theodora A.Ju, ZhaojieVaseashta, AshokJuan, AndrewVashist, Sandeep K.Judek, SlawomirVasilache, DanJudge, PhilipVasilakos, XenofonJugessur, AjuVasilateanu, AndreiJulrat, SakolVasilescu, AlinaJun, Martin Byung-GukVasiliev, AlexeyJung, AndrásVassallo, Raquel FrizeraJung, Hyung-SupVasylieva, NataliaJung, JasonVatavu, AndreiJung, SunghunVautrin-Ul, ChristineJung, YongwonVaz, CarlosJung, YunhoVazquez Garcia, CarmenJungeblut, ThorstenVázquez, Andrés S.Juránek, RomanVázquez, CarlosJusas, VaciusVazquez, Rebeca MartinezJwo, Dah-JingVčelák, JanKaabouch, NaimaVeerbeek, JanneKabir, AbuzarVeettil, Sreeja VadakkeKabiri Ameri, ShidehVega, Francisco M.Kaboli, MohsenVega, MiguelKacem, NajibVeganzones, Miguel AngelKacem, ThabetVegas, JesúsKacik, DanielVehkaoja, AnttiKaczmarek, MariuszVelázquez, RamiroKaczmarek, MichałVelez, GorkaKaddoum, GeorgesVelotta, RaffaeleKafarski, MarcinVelotto, DomenicoKahirdeh, AliVelten, AndreasKahng, SungtekVenčkauskas, AlgimantasKaimakamis, Evangelos K.Vendittozzi, CristianKaiser, ThomasVenette, Steven J.Kaiwartya, OmprakashVenini, PaoloKajimoto, ShinjiVenkat, VangavetiKakarountas, AthanasiosVenkataraman, RajeshKaklauskas, ArturasVenkatesan, RajasekarKalamkar, Sanket S.Venkatesan, RamachandranKalantar-Zadeh, KouroshVenkatesh, A.G.Kalayci, Tahir EmreVenkateswara, KrishnaKalkman, JeroenVentura, Joao OliveiraKallen, VictorVenturini, FrancescaKalliovaara, JuhaVenugopal, NiranjanKalman, HaimVenuti, GiovannaKalogianni, DespinaVerbelen, TimKaltiokallio, OssiVerbree, EdwardKamali, SaeedVerde, FrancescoKamamichi, NorihiroVerdini, FedericaKamanina, Natalie V.Verdun, JéromeKamarianakis, YiannisVereshchaka, Alexey A.Ka-Meng, LeiVergara, DiegoKameoka, JunVerhagen, SandraKamezaki, MitsuhiroVermeer, MartinKamienkowski, Juan EstebanVerona, EnricoKamienski, Carlos AlbertoVeronese, FabioKamilaris, AndreasVeronis, GeorgiosKamimura, TakafumiVersacci, PaoloKamisalic, AidaVerstichel, StijnKampmann, PeterVerwaeren, JanKamsu-Foguem, BernardVerweij, MartinKamyar, RezaVespignani, MassimoKan, Chi-waiVestergaard, Mun’delanji C.Kan, ZhenVettore, AntonioKanaev, AndreyVezzani, RobertoKanarachos, StratisVezzetti, EnricoKandammathe Valiyaveedu, SreekanthViassone, MilenaKandris, DionisisVicent, José F.Kanematsu, HideyukiVicente, Ferreira De Lucena JuniorKang, BooJoongVicinanza, DiegoKang, ByungseokVictores, Juan G.Kang, ChulhunVidal-Álvarez, GabrielKang, DaeshikVidal-Verdu, FernandoKang, Do Hyuk ’DK’Vidal-Verdú, FernandoKang, Do HyunVidev, StefanKang, InpilVidic, JasminaKang, JamesVidoni, RenatoKang, JeeunViefhues, MartinaKang, Jin S.Viegas, VítorKang, LeiVieira, D. A. G.Kang, Li-WeiVieira, TaianKang, Min GyuVien, Quoc-TuanKang, Moon GiViens, MartinKang, MyeongsuViespe, CristianKang, SanggyuVigneron, AntoineKang, Soon JuVignoli, ValerioKang, Suk-JuViik, JariKang, YuejunVikas, VisheshKangas, MichaelVilajosana, XaviKangin, DmitryVilanova, XavierKaniewski, PiotrVilcu, Gabriel EduardKanik, MehmetVilla, FedericaKanno, TakahiroVilla, PaoloKano, ShinyaVillagra, VictorKanso, HusseinVillagrossi, EnricoKantarci, BurakVillani, Maria GabriellaKanter, Theo G.Villar, José R.Kańtoch, EliaszVillas-Boas, Paulino R.Kanuk, JanVillatoro, JoelKao, Hung-AnVilleneuve, EmmaKaplan, AndreyVilliger, MartinKaplon, JanVinagre, EugéniaKapoulas, VaggelisVincent, Robert K.Kapuscinski, TomaszVincenzo, ContiKarabinis, Athanasios I.Vinogradov, SergeyKarakatsanis, Nicolas A.Vinogradov, Sergey L.Karakatsanis, Nikolaos A.Vinuesa, RicardoKaraliopoulos, MerkouriosVinueza Naranjo, Paola GabrielaKaranis, PanagiotisViola, FabioKarargyris, AlexandrosViolakis, GeorgiosKaratzas, KostasVirdis, AntonioKarayannis, ChrisViricelle, Jean PaulKarczmarek, PawełVirzonis, DariusKargas, GeorgeVisconti, PaoloKari, ChadiVisentin, FrancescoKarim, LutfulViskadourakis, Zacharias A.Karim, Mohammad RezaulVitacca, MicheleKarimi, HassanVitaletti, AndreaKarimi, Hassan A.Vítek, StanislavKarimov, ArturViter, RomanKarkanis, Stavros A.Vitetta, GiorgioKaroń, GrzegorzVitry, PaulineKarsai, ArpadVivas Albán, Oscar AndrésKarvonen, JuhaVlad, DiaconitaKaryotis, VasileiosVladeanu, CalinKasalynas, IrmantasVladyko, AndreiKašalynas, IrmantasVladymyrov, MykhailoKasapakis, VlasiosVlahu-Gjorgievska, ElenaKashevnik, AlexeyVo, B.-N.Kashino, KunioVogel, ThomasKasneci, EnkelejdaVoglhuber-Brunnmaier, ThomasKasprowski, PawełVogt, MartinKasprzak, WlodzimierzVohland, MichaelKassal, PetarVoicu, SIKassanos, PanagiotisVoiculescu, IoanaKasser, MichelVolk, JánosKästner, ChristianVollmer, MichaelKastner, WolfgangVolmer, MariusKateris, DimitriosVolná, EvaKatina, Polinpapilinho F.Volosencu, ConstantinKatsaros, KonstantinosVoloshin, Arkady S.Katsura, ShinjiVona, BarbaraKatunin, AndrzejVorwerk, JohannesKaur, AmandeepVoß, StefanKaur, AmanpreetVougioukas, EmmanouilKaur, AmardeepVoulodimos, AthanasiosKauranne, TuomoVourna, PolyxeniKaushal, HemaniVoutetaki, Maria–StylianiKaushik, AjeetVoyant, CyrilKavakli-Thorne, ManolyaVozel, BenoitKavehei, OmidVozzi, GiovanniKawahara, TomohiroVrigneau, BaptisteKawalec, AdamVu, Hai CanhKawamura, KensukeVu, Ngoc SonKawanishi, YasutomoVujaklija, IvanKawarazaki, NoriyukiVukovic, NatashaKawoos, UsmahVulic, MilivojKaya, TolgaVulpe, AlexandruKazimierski, WitoldVyskočil, VlastimilKazuhiro, KondoWackerlig, JudithKazys, RymantasWackrow, ReneKebkal, KonstantinWada, DaichiKedzierski, MichalWada, YujiKefauver, Shawn CarlisleWaddell, EmanuelKehl, FlorianWadduwage, Dushan N.Keler, AndreasWade, Eric R.Keller, DustinWagnac, EricKeller, MatthiasWagner, BernardoKellnhofer, PetrWagner, Simon AKelly, Christopher V.Wagner, StevenKelly, JamesWagner, ThorstenKelner, JanWahlström, JohanKemker, RonaldWahrburg, ArneKemper, BjörnWajman, RadosławKennedy, DavidWalas, KrzysztofKennett, RonWalczak, KrzysztofKenter, AlmusWaldmann, ChristophKeogh, EamonnWaldmann, DanièleKeogh, PatrickWalendziuk, WojciechKeplinger, TobiasWalgraeve, ChristopheKepski, MichalWalker, AlanKereszturi, AkosWalker, AnthonyKereszturi, GaborWalker, Harrison C.Kerkides, Petros G.Walker, JacquelineKerman, KaganWalla, PeterKern, StefanWallace, R BruceKerr, DermotWallscheid, OliverKersten, TobiasWalsh, Kerry BKersten-Oertel, MartaWalter, Jonathan PKeser, TomislavWalters, E. GeorgeKeshavarz Hedayati, MehdiWaltl, MichaelKeshtkaran, Mohammad RezaWan, BaolinKeskinarkaus, AnjaWan, FengKeski-Saari, SaritaWan, HaoKhaksar, WeriaWan, Kai TaiKhalaf, WalaaWan, TaoKhalifa, SaraWandowski, TomaszKhaliq, JibranWang, AiningKhaloo, AliWang, BangKhalsa, SahibWang, BoKhambhati, Ankit N.Wang, CaixiaKhamukhin, AlexanderWang, Cheng-LiangKhan, A. MohammadWang, Chi-KueiKhan, Atif AliWang, Chiu-YenKhan, FaheemWang, ChunlingKhan, HamzaWang, CongKhan, M. ArsalanWang, DadongKhan, Md Muhidul IslamWang, DanlingKhan, NaserWang, DianhuiKhan, PervezWang, DingKhan, TareqWang, DongKhan, WajahatWang, Dung-AnKhanna, RaghavWang, FeiKhanna, ShrutiWang, Fu-KangKhatibi, Akbar A.Wang, FukeKhatibi, MiladWang, GangKhatir, SamirWang, GuochaoKhenchaf, AliWang, GuodongKhodabandeh, AmirWang, GuoquanKhodadad, DavoodWang, HaoKhodaei, ZahraWang, HongboKhodasevych, IrynaWang, HongquanKhodasevych, Iryna E.Wang, HongwuKhorov, EvgenyWang, HuaKhoshelham, KouroshWang, HuaqingKhoshmanesh, KhashayarWang, HuizhiKhosla, AjitWang, Jia-JungKhouakhi, AbdouWang, JianfengKhyam, Mohammad OmarWang, Jianguo JackKia, Seyed MostafaWang, JianzhouKia, Solmaz S.Wang, JiaoKiang, Jean-FuWang, JidongKianoush, SanazWang, JingKiefer, PeterWang, JinqiaoKieninger, JochenWang, Jin-YuanKierat, WojciechWang, JuKiersztyn, AdamWang, JunhongKihei, BillyWang, KaiKikuchi, HiroakiWang, KanKillane, IsabelleWang, KangKim, AlbertWang, KevinKim, BongjinWang, KezhiKim, Byeong HeeWang, LanKim, Byeong-GyuWang, LeiKim, Byung-GyuWang, LiangKim, ChangjaeWang, LifengKim, CheonshikWang, LinKim, ChulhongWang, MiKim, DaeEunWang, Ming-shiKim, DaeheeWang, MingshuKim, Dae-YoonWang, Ming-ShyanKim, Do HwanWang, Min-HawKim, Do YoungWang, Mu-chunKim, DonghanWang, NingKim, Dong-JooWang, PengfeiKim, DongseobWang, PengtaoKim, Dong-SeongWang, PichaoKim, Du YongWang, Pi-ChungKim, Duck YoungWang, PingKim, DukhyeonWang, PoKim, Felix SunjooWang, QiKim, Gi-WooWang, QiaosongKim, Hee-SeokWang, QingKim, Ho-kyungWang, QingQingKim, HwangnamWang, QixinKim, HyogonWang, S.Kim, Hyoung-NamWang, ShangguangKim, Hyug-HanWang, ShaohuaKim, Hyun JaeWang, ShaopengKim, HyunbumWang, ShengkaiKim, Hyung SeokWang, Sheng-ShihKim, HyungminWang, ShuhangKim, Hyung-sinWang, TengKim, Ill WonWang, WeiKim, In-HoWang, WenKim, InsooWang, WenjingKim, Jae JoonWang, WentaoKim, JanghoWang, WenyiKim, JayoungWang, XiangrongKim, JeongraeWang, XiaoyanKim, Jin-HyukWang, XinKim, JinwookWang, XingweiKim, JonghoekWang, XuanKim, Jong-HoonWang, XudongKim, Jong-HwanWang, XueweiKim, JongweonWang, XuguangKim, JoohwanWang, XuyuKim, JoongheonWang, YanKim, Ki-IlWang, YangKim, KukjooWang, Yao-TienKim, Kyeong-seopWang, YiKim, KyoohyunWang, YideKim, KyujungWang, YinKim, Kyung RokWang, YipingKim, KyungsangWang, YonghuiKim, MinjaeWang, YongxiangKim, Moon IlWang, YueKim, Myun-SikWang, Yu-LinKim, Nam-YoungWang, Yun-MingKim, RobinWang, Yu-TeKim, Sang-HaWang, ZhelongKim, SanghoekWang, ZhengKim, SangkilWang, ZhenxinKim, SangtaeWang, ZhenyuKim, SeokminWang, ZhiguangKim, Seong JinWang, ZhiyuKim, Seong-EunWannous, HazemKim, SoheeWard, JonathanKim, Sung SukWarner, MartinKim, Sung-BaeWarren-Smith, StephenKim, SunghoWas, JaroslawKim, Sung-ManWashizawa, YoshikazuKim, SunwookWasson, AntonKim, Tae HyunWatanabe, FumiyaKim, TaehongWatanabe, ShinichiKim, Tae-HyungWatier, BrunoKim, Tae-ilWatkins, A. NealKim, TaesicWatson, DennisKim, Tae-SungWatson, RobertKim, Tai HoonWatson, SimonKim, Teun-TeunWaxman, Justin P.Kim, WansooWay, MichaelKim, WonheeWazid, MohammadKim, WonjunWebb, Michael R.Kim, Yong-GukWeber, AndreasKim, Yong-HoonWeber, FelixKim, Yong-HwaWeber, KarinaKim, Young GabWeber, KonradinKim, Young-ChonWeber, RobertKim, Young-DukWeber, ThomasKim, YoungilWebster, ChristopherKim, YoungjooWebster, EricKim, YoungokWebster, JohnKim, Young-RokWebster, John G.Kimoto, ShigeruWeddell, AlexKinahan, David JWegener, JoachimKincses, ZoltánWehrmeister, MarcoKind, Matias CarrascoWei, DongyanKinet, DamienWei, GangKing, Eoin A.Wei, GuohuaKinugawa, JunWei, HemingKirchhoff, Jon R.Wei, KayaKirchner, KathrinWei, MenglianKirchner, MatthiasWei, QingshanKirei, Botond SándorWei, QingshuoKirkko-Jaakkola, MarttiWei, Shih-ChungKiselev, IliaWei, WeiKish, LaszloWei, YangKisner, AlexandreWei, ZhenzhongKiss, BálintWeidinger, TamásKissinger, ThomasWeigel, MartinKitani, TomoyaWeigel, RobertKitano, ClaudioWeinberg, MarcKiyono, KenWeintrit, AdamKlantsataya, ElizavetaWeiss, ThomasKlauberg, CarineWeissgerber, FloraKlein, ItzikWeitzen, Jay A.Kleinschmidt, João HenriqueWells, B. EarlKliks, AdrianWeltzien, CorneliaKlinck, HolgerWen, Chih-YuKlingbeil, LasseWen, KunliKlose, CorneliaWen, ZhenKłosowski, GrzegorzWencel, DorotaKlotzkin, DavidWendel, JanKluge, FelixWendzel, SteffenKluge, TilmannWenger, ChristianKnaflitz, MarcoWeppner, JensKnauth, StefanWerle, DirkKnight, ChristopherWerner, SteffenKnoll, AloisWest, Simeon J.Knorn, SteffiWesterdahl, DaneKnowles, MikeWetterlind, JohannaKnutas, AnttiWeyn, MaartenKnypiński, ŁukaszWhang, MincheolKo, Byoung ChulWhelan, Matthew J.Ko, HyounghoWhitton, Mary C.Ko, HyunhyupWicks, MichaelKo, Kwang HeeWidger, PhillipKobayashi, FutoshiWidhalm, PeterKobayashi, HirokazuWieczorek, Piotr Z.Kobayashi, MakikoWiekhorst, FrankKobayashi, MasakiWielandt, StijnKobayashi, YoshikazuWielgosz, MaciejKobsar, DylanWientjes, EmilieKoc, Ali BulentWieringa, FokkoKoc, WładysławWierzba, PawełKochkin, PavloWierzbicki, DamianKocian, AlexanderWietfeld, ChristianKociuba, WaldemarWijnhoven, RobKockmann, MarcelWikner, JacobKocur, DusanWiktor, ArturKofidis, EleftheriosWilhelm, JayKofuji, Sergio TakeoWilkins, RossKoga, Jun-ichiroWillander, MagnusKogia, MariaWillberg, ChristianKoh, JaehanWilliams, JonathanKoh, Je-sungWilliams, Paul JKoh, Seok-JooWilliamson, CareyKöhler, BerndWilliamson, Geoffrey A.Kohut, PiotrWillis, Andrew R.Koike, KunihiroWillmott, JonKoike, YasuharuWillsch, MichaelKoike, YoshikazuWilmer, ChristopherKoinkar, PankajWilmes, AnjaKoirala, Binod PrasadWilson, AdrianKõiva, RistoWilson, AlphusKojima, FumihideWilson, Christopher G.Kokado, KentaWin, Khin ThanKokar, Mieczyslaw M.Win, Khin YinKokinou, EleniWinkler, Joab R.Kokkalis, PanagiotisWinkler, Thomas E.Kokkinos, ChristosWinter, StephanKokkinos, PanagiotisWiora, JózefKokkonis, GeorgeWischik, ClaudeKokkoris, GeorgeWitayangkurn, ApichonKokogiannakis, GeorgiosWitczak, MarcinKokubo, HisashiWitkowska Nery, EmiliaKołakowska, AgataWöhrle, HendrikKolanowski, Jacek L.Wojciechowski, AdamKolasiński, PiotrWojciechowski, WadimKolb, AndreasWójcikowski, MarekKolias, ConstantinosWojkiewicz, Jean-LucKolios, StavrosWojtas, JacekKollár, MojmirWolański, WojciechKolomvatsos, KostasWolff, MarcusKommers, PietWolffenbuttel, Reinoud F.Komodromos, MichaelWollweber, MerveKomori, MochimitsuWon, MyounggyuKoncar, VladanWondraczek, KatrinKong, DemingWong, Chi LokKong, FannianWong, Danny K. Y.Kong, Ling BingWong, DavidKong. Q.Wong, David Shan HillKong, QingzhaoWong, Kainam ThomasKong, XiangruiWong, Ka-LeungKonieczny, ŁukaszWong, Kin-hongKonorski, JerzyWong, LawrenceKonovalov, SergeyWong, Lawrence L.P.Konovaltsev, AndriyWong, LeslieKonstantas, DimitriWong, Ling TimKonstantinidis, Theocharis G.Wong, Ling-timKonstantinos, VotisWong, Steve HangKonstantopoulos, CharalamposWongkasem, NantakanKonstantynovski, KostyantinWoo, Jong-KwanKontoni, Denise-PenelopeWoo, Wai LokKontou, EleftheriaWood, Richard L.Konushin, AntonWoodhead, IanKoohbor, BehradWoods, JohnKootstra, GertWook Baik, SungKopáčik, AlojzWorch, RemigiuszKopackova, VeronikaWotzka, DariaKopelevich, OlegWoźniak, MarcinKoplin, ChristofWoznowsk, PeteKoppányi, ZoltánWright, Darryl E.Kopra, KariWright, LoganKoprinkova-Hristova, PetiaWróblewski, GrzegorzKopyt, PawełWu, An-yeuKor, Ah-LianWu, BianKorbel, PiotrWu, CelimugeKordestani, MojtabaWu, ChangshanKordi Ghasrodashti, ElhamWu, Cheng-WeiKordi, BehzadWu, ChenshengKoren, KlausWu, Chi-ChangKoreň, MilanWu, Chung-Tse MichaelKornhuber, StefanWu, ChunleiKorobiichuk, IgorWu, HangbinKorobko, Dmitry A.Wu, HuixuanKorposh, SerhiyWu, JianfengKorzun, DmitryWu, JiankangKos, AntonWu, KaiyuKos, MarkoWu, LiaoKoskela, MarkusWu, LifengKošnar, KarelWu, LinKostamovaara, JuhaWu, LongfeiKostelecký, JakubWu, Lyndia C.Kostin, Vladimir NikolaevichWu, N. EvaKostrzewski, MariuszWu, QiKosuke, SugawaWu, QingqingKothapalli, Sri-Rajasekhar (Raj)Wu, QisongKothgassner, Oswald DavidWu, ShangbinKotsiantis, SotirisWu, ShaoenKottapalli, Ajay Giri PrakashWu, Shih-JehKotyński, RafałWu, Shih-LinKotze, BenWu, Shin-TsonKouba, JanWu, Shu-PaoKouchaki, SamanehWu, Tzong-ChenKoucheryavy, AndreyWu, Wei-ChiangKoulamas, ChristosWu, XiaoKoulaouzidis, AnastasiosWu, XiaofengKoumela, AlexandraWu, XuejianKoumoulis, DimitriosWu, YiKouris, LASWu, ZhengKõuts, TarmoWu, ZhishenKoutsoudis, AnestisWu, ZhongKoutsoukos, XenofonWu, ZiyanKoutsoureli, MatroniWujanz, DanielKoutzarova, TatyanaWujcik, EvanKouyoumdjieva, SylviaWydra, MichalKouznetsov, Rostislav D.Wysocki, MarianKovalevsky, Andrei V.Xavier-de-Souza, SamuelKowalczyk, MarcinXhakoni, AdiKowalczyk, SebastianXia, XiaohuKowalski, LukaszXian, XiaojunKowalski, PiotrXiang, DeliangKowerko, DannyXIANG, WeidongKoyama, Christian N.Xiang, XianboKoyuncu, ErdemXianyu, YunleiKozak, Drazan,Xiao, FengKozlov, AlexanderXiao, LiangKraatz, Heinz-BernhardXiao, WenKraatz, SimonXiao, XuKraft, MarekXiao, YongKrajnik, TomasXie, JieKralevska, KatinaXie, JinKraniotis, DimitriosXie, LeiKrasiński, AdamXie, ShangranKrasnoslobodtsev, Alexey V.Xie, XiufengKrasteva, VesselaXie, YongKratzke, NaneXie, ZhijianKrause, ColleenXing, FeiKrause, Hans-JoachimXiong, HuilinKrauze, PiotrXiong, KeKravchenko, Ivan IXiong, NaixueKravchenko, VictorXiong, RuiKrejcar, OndrejXu, ChongKrell, Mario MichaelXu, GuirongKremens, RobertXu, GuobaoKrief, FrancineXu, HeKrishna Gumma, MuraliXu, HongliKrishnan, SiddharthXu, HuijuanKrisp, Jukka MatthiasXu, JianKritikos, KyriakosXu, JiangtaoKrivetskiy, ValeriyXu, JiaqiangKroeger, SilkeXu, LisongKról, MichałXu, LizhiKrolczyk, GrzegorzXu, LuKrólczyk, GrzegorzXu, MingweiKrólczyk, JolantaXu, NanKrólicka, AgnieszkaXu, Sheng-yongKromanis, RolandsXu, TaoKronander, JoelXu, TianhuaKroos, ChristianXu, TingtingKropf, JohannesXu, TongyangKroupa, MartinXu, WeiKrug, RobertXu, WeitaoKrüger, BjörnXu, WenjunKrull, ClaudiaXu, XiaoguangKruss, SebastianXu, XiaohuaKryjak, TomaszXu, XiaolinKrylov, Vladimir A.Xu, XiaominKrypiak-Gregorczyk, AnnaXu, XiuruKrzeszowski, TomaszXu, YongKrzysztof, NausXu, YuehangKrzywiecki, ŁukaszXu, ZheKtena, AphroditeXuan, HaifengKubanek, MariuszXuan, ShouhuKube, ChristopherXyrichis, AndreasKubik, ZdenekYadav, MarshleenKubo, IzumiYahiaoui, RédaKubryakov, ArsenyYahiaoui, RiadKuc, Tae-YongYalin, Azer P.Kučera, MariánYamada, IchiroKučerová, AničkaYamamoto, KyosukeKudela, PawelYamamoto, ToshiyukiKudryavtsev, VladimirYamamura, ShoheiKuijk, MaartenYamana, KazushigeKujala, TuomoYamane, SatoshiKuladinithi, KoojanaYamasaki, YuichiKularatne, DhanushkaYamazoe, NoboruKulathumani, VinodYan, HongxiangKulesza, WlodekYan, JinKulinich, SergeiYan, QiaoKulka, JozefYan, QibenKulkarni, AtulYan, ShuangyiKulkarni, KedarYan, WaiyeungKulyukin, VladimirYan, YanjunKumar Jena, ChinmayYañez-Sedeño, PalomaKumar Mishra, YogendraYang, BaijianKumar, A. AnilYang, ChengKumar, Ganesh P.Yang, ChenguangKumar, Jitendra (Jitu)Yang, Chia MingKumar, ManojYang, Chih-TsungKumar, PankajYang, DongkaiKumar, PradeepYang, Eileen Chih-YingKumar, SajeeshYang, FanKumar, ShailabhYang, FangKumar, Suryadevara NagenderYang, FengKumar, VimalYang, GuangKumar, VivekYang, HaifengKundu, TribikramYang, HongKung, Chung-WeiYang, Hsiao-YuKung, Hsu-YangYang, HuaKung, Yu-ChunYang, JasonKunkel, JulianYang, JianhuaKunze, KaiYang, JieKuo, Ching-ChangYang, John XunKuo, Chung-HsienYang, Jong-RyulKuo, Tsung-RongYang, Josh Tsun-HuaKuo, Tung-WeiYang, JunjingKuo, Yaw-WenYang, KaiyuanKuo, Yong-LinYang, LanKuok, Sin-ChiYang, Lee-XiengKurabayashi, DaisukeYang, LiangliangKurabayashi, ShuichiYang, LilyKuravi, SaradaYang, LinghuiKurda, RawazYang, LipingKurdi, HebaYang, Mau-TsuenKurihara, ToruYang, MijiaKuritka, IvoYang, MingKurkina, TetianaYang, Ming-DerKurlyandskaya, Galina V.Yang, ShaowuKuroda, RihitoYang, ShimingKurouski, DmitryYang, SungKurrant, DougYang, Sung-BongKurum, MehmetYang, TingKurzatkowska, KatarzynaYang, Wei-BinKusne, Aaron GiladYang, WenchengKusnerova, MilenaYang, WuqiangKussul, NataliiaYang, XiaoleiKusyk, JanuszYang, XuKuttner, ChristianYang, YangKuusik, AlarYang, YiKuznetsov, YuryYang, YuanKuzuhara, DaikiYang, Yuan-SenKwak, DaehanYang, YunjieKwak, Jin TaeYang, YunzeKwak, Keun-ChangYang, ZhengKwak, Moon K.Yang, ZhenyuKwan, ChimanYang, Zhi-BoKwasnicki, Richard MarkYannis, GeorgeKwok, Ngai MingYano, AkiraKwon, Goo-RakYanushkevich, Svetlana N.Kwon, JunseokYao, DezhongKwon, MinhaeYao, FanKwon, Oh JoongYao, JunKwon, Soon-HongYao, LeehterKwon, Soon-wookYao, LinKwon, Sun IlYao, LinaKwon, Tae J.Yao, TianKwon, Tae-YubYao, TiankaiKwon, YoungeunYao, XiaKyamakya, KyandoghereYao, ZhengKycko, MarlenaYapici, Murat KayaKyslan, KarolYaqoob, IbrarKyung, Ki-UkYardimci, Nezih TolgaLa Malfa Ribolla, EmmaYarnold, MatthewLa Spada, LuigiYassine, AbdulsalamLa Spina, RitaYasuoka, YumiLaamrani, AhmedYasutomi, KeitaLabardi, MassimilianoYasyukevich, YuryLabati, Ruggero DonidaYavari, EhsanLabib, MahmoudYazdani Nezhad, HamedLabiod, HoudaYazdani, AmirLabuta, JanYazdkhasti, SetarehLacava, TeodosioYazidi, AnisLacaze, BernardYe, EnyiLacidogna, GiuseppeYe, HangLackovic, IgorYe, HuaiyuLadani, RajYe, QunLaefer, DebraYe, WeilinLaefer, Debra FYe, YanfangLaewunruen, SakdiratYe, YuLaflamme, SimonYe, YuanLagarde, FlorenceYeh, Chien-HungLage, EnnoYeh, Hen-Geul (Henry)Lagkas, ThomasYeh, Kuo-HuiLagudi, AntonioYeh, Sheng LihLahtela, VilleYeh, Sheng-ChengLai, AntoniaYeh, Yin-TingLai, BinYehezkeli, OmerLai, Chao SungYelamarthi, KumarLai, Chin-FengYen, Li-HsingLai, ChinLunYeo, Woon-HongLai, Chiu-KengYeom, EunseopLai, CristianYeom, SeokwonLai, Po-HsiangYeong, WaiLai, PuxiangYesilkoy, FilizLai, StefanoYeum, Chul MinLai, Ying-ChihYi, JingangLaidig, DanielYi, June-HoLajos, NagyYi, TinghuaLalande, Jean-FrancoisYi, XinpingLalléchère, SébastienYifat, YuvalLam, Kwok-hoYildirim, Kasim SinanLamberti, AlfredoYilmaz, HuzeyfeLamberti, FabrizioYilmaz, MehmetLameiro, ChristianYim, SehyukLami, IhsanYin, FanghuiLamonaca, FrancescoYin, GuodongLampert, AstridYin, JunjunLampoltshammer, ThomasYin, PenghangLan, GuohaoYin, ShouyiLan, Kun-ChanYin, WuliangLanceros, SenentxuYin, XiangLancis, Basam MuslehYin, XiaoluLancis, JesúsYing, KaiLandaluce, HugoYli-Kaakinen, JuhaLandowska, AgnieszkaYoe, HyunLandulfo, EduardoYogeesh, MaruthiLaneve, GiovanniYoma, Néstor BecerraLang, Hans PeterYoneda, KeisukeLang, JochenYoo, ByungseokLang, MichaelYoo, Hyeon JoongLang, UdoYoo, JaehyunLange, Benjamin A.Yoo, Seong-eunLangford, ZacharyYoo, YounghwanLanghammer, BirgittaYoon, BoraLangley, DanielYoon, GilwonLangley, PhilipYoon, HyosangLangone, RoccoYoon, HyungchulLantsoght, Eva Olivia LeontienYoon, IkjuneLanz, OswaldYoon, Ji-WookLanza-Gutierrez, Jose M.Yoon, JongheeLanzolla, Anna M. L.Yoon, JonghoLapray, Pierre-JeanYoon, MinyoungLari, ZahraYoon, SeokhoonLarijani, HadiYoon, YokeLarson, ChrisYorgos, StephanedesLarsson, ChristerYoshioka, HirokiLary, David JYou, Cheol HwanLashkari, BahmanYou, XingeLaski, Pawel AndrzejYoun, Ik-HyunLasky, TyYoung, AaronLasri, TuamiYoung, KymberlyLassen, MikaelYoungjoo, KimLatif, TahmidYousefi, BardiaLatif, YasirYousif, Jabar H.Lattanzi, DavidYoussef, TarekLattanzio, Veronica Maria TeresaYoussf, OsamaLatychevskaia, TatianaYu, Chih-MinLau, DenvidYu, Chin-pingLau, MichaelYu, ChunyangLauneau, PatrickYu, HanLaura, MorettiYu, JiayongLaureti, StefanoYu, Ki JunLautner, GergelyYu, ManzhuLavagetto, FabioYu, ShengweiLavagno, LucianoYu, ShiqiLavalliere, MartinYu, Tzyy LeonLavine, BarryYu, WenxianLavric, AlexandruYu, XinLavrova, OlgaYu, XunLaw, Darin J.Yu, YangLaw, Yee WeiYu, YanguangLawrynczuk, MaciejYu, YifanLawson, CharlotteYu, YingjieLay-Ekuakille, AiméYu, YunLazar, ZsofiaYu, ZetaLazarescu, Mihai TeodorYuan, BingLazaridis, PavlosYuan, JieLazaro, AntonioYuan, LeiLázaro, JesúsYuan, QiangqiangLázaro, José L.Yuan, QilongLázaro, María TeresaYuan, QuanLázaro-Galilea, José L.Yuan, Shyan-MingLazaroiu, George CristianYuan, WenzhenLazarou, Georgios Y.Yuan, ZhenLazarou, StavrosYue, JingLazarov, AndonYue, LanLazarus, NathanYue, TaoLazecky, MilanYue, WenzhenLe Bars, FabriceYueksel, HazarLe Bas, Pierre-YvesYuen, ChauLe Bastard, CédricYuen, PeterLe Bihan, FranceYufera, AlbertoLe Kernec, JulienYun, Ji-HoonLe Moullec, YannickYun, SangkiLe Traon, OlivierYun, YoungsunLe, Hieu HanhYunusa-Kaltungo, AkiluLe, NamTuanYurovsky, Yury YuLe, Trung KienYuste-Delgado, A.J.Le, TuanZabala, MichaelLe, ZhangZaballos Diego, Juan AgustínLeach, RichardZabalza, JaimeLeal-Junior, ArnaldoZabek, DanielLeandro, DanielZabini, FlavioLebedev, Alexander AlexandrovichZaborowicz, MaciejLebel, KarinaZachariades, ChristosLebioda, MarcinZagajewski, BogdanLeBlanc, RogerZagar, BernhardLecacheux, AlainZaghari, BaharehLeccese, FabioZagrodny, BartłomiejLechene, Balthazar (Pierre)Zaharia, GheorgheLechler, ArminZahiri, MohsenLedo, AnaZahorian, Stephen A.Lee, E.C.Zaidi, Shabi AbbasLee, AhyoungZaitsev, Dmitry L.Lee, Boon GiinZakaria, AmerLee, Byung ChulZalama, EduardoLee, Byung MooZalejska-Jonsson, AgnieszkaLee, ChaBumZaltron, AnnamariaLee, Chang-HunZalyubovskiy, VyacheslavLee, ChangwonZamay, TatianaLee, Chao-HsienZambon, IlariaLee, Cheng-ChiZaminpardaz, SafooraLee, Che-RungZamora, WillianLee, Chia-HungZanchetta, GiulianoLee, Christopher H. T.Zang, YapingLee, DaehoZangeneh-Nejad, FarzadLee, Dae-SikZangle, ThomasLee, En-yuan JoshuaZangróniz, RobertoLee, Gi-JaZanlungo, FrancescoLee, GyuZapata Sierra, AntonioLee, Gyu MyoungZappa, GiovanniLee, Hsiang-ChiehZappatore, MarcoLee, Hyeong JaeZarifi, Mohammad H.Lee, Hyo JongZarouchas, DimitriosLee, HyokiZarpalas, DimitrisLee, HyoungsoonZarpelão, BrunoLee, Hyung-MinZarpelão, Bruno BogazLee, HyunjaeZarri, Gian PieroLee, Hyunjoo JennyZarrin, JavadLee, HyunsooZatelli, PaoloLee, Il-GuZaťko, BohumírLee, Jae OneZawadzki, JarosławLee, Jae WoongZayas, Almudena DiazLee, JaehongZazpe, RaulLee, JaehwangZega, ValentinaLee, JaesungZegarra Flores, JesusLee, JaeyoungZeiri, LeilaLee, JangmyungZeitler, AxelLee, JayZelensky, Nikita P.Lee, JihoonZema, Demetrio AntonioLee, JinseokZembaty, ZbigniewLee, Jin-YongZemcik, PavelLee, Jong-HaZemková, ErikaLee, Jong-HeunZeng, HulieLee, Jong-JaeZeng, JiangyuanLee, Jong-SeokZeng, ShuwenLee, JongSubZeng, YeluLee, JuZeng, YijianLee, Jung HoonZeng, YongLee, Jung KeunZennaro, MarcoLee, Jung-RokZet, CristianLee, Jung-RyunZettel, JohnLee, Jun-HakZetterling, Carl-MikaelLee, JunyoungZeydan, EnginLee, Ju-YiZhai, LiangLee, KangwonZhang, A.PingLee, KeekeunZhang, BaochengLee, KeunhwaZhang, BinLee, Min-HoZhang, ChaoLee, Moon GuZhang, ChengLee, MoonjinZhang, ChiLee, SangyoonZhang, DanLee, Sang HunZhang, DimingLee, Sang HyunZhang, FengjiaoLee, Sang WookZhang, GongLee, SanghoZhang, GuangmingLee, Sang-MaeZhang, GuoLee, SangukZhang, GuoqingLee, Sang-WoongZhang, GuoshengLee, SangyounZhang, HaiLee, Seok JaeZhang, Haichong K.Lee, Seong-CheolZhang, HaihongLee, Seung-BeckZhang, HaijunLee, Seung-JungZhang, HankuiLee, Shie-jueZhang, HaoLee, Soo SukZhang, HaopengLee, Suk JinZhang, HongLee, SungonZhang, HongshengLee, SunwooZhang, HuanxinLee, Tae YoonZhang, HuiLee, TaekkyeunZhang, JianzhongLee, TaeyoonZhang, JiashuLee, TaikjinZhang, JiaxinLee, Tian-FuZhang, JieLee, Victor R.Zhang, JingjingLee, Wen-YaZhang, JingxiongLee, WoncheolZhang, JinnanLee, Woo-KyungZhang, JitaoLee, Yao-JenZhang, JunLee, Yih HongZhang, KuanLee, YoongduZhang, KuangLeedle, Kenneth J.Zhang, LeiLeeke, MatthewZhang, LiangpeiLees, John E.Zhang, LifuLefebvre, GrégoireZhang, LigangLegleiter, CarlZhang, LiqiangLe-hegarat, SylvieZhang, MingzheLehmann, RüdigerZhang, MuLehtola, VilleZhang, QilinLei, ChongZhang, QingLei, Kin FongZhang, QinqinLei, MingZhang, RuiLeightley, DanielZhang, RunLeister, WolfgangZhang, ShaolinLeitão, CátiaZhang, ShuaiLeite Filho, Waldemar C.Zhang, ShuqingLeitner, GerhardZhang, SijieLeliaert, JonathanZhang, SongsongLemaire, EdwardZhang, SuLemeune, AllaZhang, TingtingLemon, ChristopherZhang, TishengLeMoyne, RobertZhang, TongLenau, TorbenZhang, WengangLengden, MichaelZhang, WenjunLenik, JoannaZhang, WentaoLenzo, BasilioZhang, WenzengLenz-Wiedemann, VictoriaZhang, WumingLeo, MarcoZhang, XiLeón, CarlosZhang, XianmingLeón, JulioZhang, Xian-MingLeonard, GregZhang, XiaoLeonardi, FrancescaZhang, XiaochenLeonardi, Salvatore GianlucaZhang, XiaotongLeonarduzzi, Roberto FabioZhang, XinMingLeonesio, MarcoZhang, XuLepore, MariaZhang, XudongLeppäkoski, HelenaZhang, XueboLera, FranciscoZhang, XuyunLerga, JonatanZhang, Ya-nanLerma, ClaudiaZhang, YanjieLeroux, Ian DanielZhang, YanningLeroux, PaulZhang, YanxiaLesch, AndreasZhang, YewenLesiak, PiotrZhang, YiLeso, VerusckaZhang, YonggangLete, CeciliaZhang, YongguangLetu, HusiZhang, YuLeukhin, AnatoliiZhang, YuanzhiLeung, Hui MinZhang, YudongLeung, Jonathan Y.S.Zhang, YumingLeuschen, CarlZhang, YunshengLevanon, NadavZhang, YushuLevi, AlessandroZhang, YutingLevin, NoamZhang, ZhaoweiLevine, MindyZhang, ZhenjiangLewis, GregoryZhang, ZhidongLhotska, LenkaZhang, ZhiguoLi, BaihuaZhang, ZhouLi, BaoqiangZhang, ZiyangLi, BeibeiZhang, ZonghuaLi, BeiwenZhao, BoxuanLi, BingZhao, ChunLi, BoZhao, DonglinLi, ChengmingZhao, HangLi, ChongZhao, HubinLi, Chun-HsingZhao, HuichanLi, DachuanZhao, JingzhouLi, De-junZhao, KaichuLi, DeminZhao, LianLi, DeshiZhao, MingjunLi, DinghuaZhao, NaizhuoLi, DuanZhao, PengxiangLi, FeiZhao, ShuheLi, FuhuaZhao, ShunyiLi, GangZhao, SichengLi, GuofaZhao, SihaoLi, GuoyuanZhao, WenbingLi, HaijunZhao, WenfengLi, HaoZhao, XianhuiLi, HongZhao, XinxinLi, JianZhao, YangLi, JianliZhao, YanliLi, JianzhaoZhao, YiliangLi, JinZhao, YongliLi, KangZhao, ZhanLi, KezhiZhao, ZhongliangLi, LeiZhao, ZongshanLi, LiangZhao, ZuofengLi, LinZharkova, Galina MLi, LongjiangZheng, GuanglouLi, NaZheng, GuoanLi, NingZheng, Hai-TaoLi, PanZheng, Jia-XinLi, PeijunZheng, LingxiangLi, QingguoZheng, PaiLi, RuyaZheng, QingbinLi, ShuoZheng, QiyeLi, SongnianZheng, XintingLi, TaoZheng, YinqiangLi, TianchengZheng, YuanjinLi, WeiZheng, ZhongLi, WeijieZhong, LihengLi, WenjuanZhong, YongminLi, WenliangZhong, ZhangduiLi, WenlingZhou, AoLi, XiaoZhou, BingpuLi, XiaofengZhou, BoLi, Xiaohua (Edward)Zhou, CimingLi, XiaojunZhou, FengLi, XiaoluZhou, HaiboLi, XiaopengZhou, HailingLi, XinmingZhou, HongliangLi, XueyouZhou, JianhuaLi, XuranZhou, JiehanLi, YanhuaZhou, MengChuLi, YanshengZhou, QiangLi, YeZhou, QifanLi, YiZhou, QinLi, YinZhou, QuLi, YiyanZhou, Xiao DongLi, YongZhou, XiaoboLi, Yung-HuiZhou, YufengLi, YunpengZhou, YunlaiLi, YunziZhou, ZhenyuLi, ZhengZhou, ZimuLi, ZhenhaiZhu, AijunLi, ZhibinZhu, BenpengLi, ZhijieZhu, BowenLi, ZhixiongZhu, ChenxinLi, ZhiyiZhu, ChunshengLi, ZhongliangZhu, DakaiLi, ZijianZhu, FengLi, ZishenZhu, HepingLi, ZuchuanZhu, HongziLian, IanZhu, LingliLiang, ChengchaoZhu, PeihuaLiang, Chia-PinZhu, QingyuanLiang, Chiu-kuoZhu, XiaolinLiang, HaoZhu, YananLiang, HuaweiZhu, YuhaoLiang, HuiZhu, YungangLiang, JianmingZhu, ZhangmingLiang, JieZhu, ZheLiang, JinxingZhu, ZhigangLiang, JiuzhenZhu, ZhiguangLiang, MengbingZhuang, BohanLiang, Tsair-ChunZhuang, JunLiang, XinwuZhuang, YuanLiang, YanZhukova, ValentinaLiao, BinZhukowski, PawelLiao, Chun-FengZhuo, YueLiao, Jan-RayZi, BinLiao, Jiunn-DerZi, YunlongLiao, Kuo-ChihZidansek, AleksanderLiao, Tien-HaoZiębiński, AdamLiao, Yi-HungZiebold, RalfLiaparinos, Panagiotis F.Ziegler, DominikLicitra, GaetanoZikova, NadezdaLie, ArneZikria, Yousaf BinLienhart, WernerZilic, ZeljkoLiesenberg, VeraldoZilinskas, AntanasLiKamWa, RobertZimbelman, Eloise G.Lim, Deok WonZimmermann, AlfredLim, GeunbaeZimmermann, StefanLIM, GilbertZingg, Jean-MarcLim, Hyung JinZinno, RaffaeleLim, Jae-HanZiółkowski, MarcinLim, Kwan HuiZiolkowski, MarekLim, SejoonZiolkowski, RobertLim, SungjoonZito, Giuseppe AngeloLim, Yah LengZivanovic, StanaLim, Yee YanZivcak, MarekLima, José LuisZiyatdinova, GuzelLin, Chang HongZlatanova, SisiLin, Chia-HerZografopoulos, DimitriosLin, Chien-ChouZolfagharian, AliLin, Chien-hongZollhöfer, MichaelLin, Chih-mingZolotová, IvetaLin, Chih-PingZorbas, DimitriosLin, Chin-FengZorzi, MattiaLin, ChingShunZotti, AldobenedettoLin, Chi-YingZou, Fangxin (Frank)Lin, Chu-EnZou, LiangLin, Chun-LingZou, QinLin, Chun-WeiZou, QiongjingLin, FengZou, XiangjunLin, Gong-RuZou, YuLin, GungunZubair, MuhammadLin, Hao-TingZubizarreta, AsierLin, Hsueh-chunZucali, MaddalenaLin, Huei-YungZuccarello, BernardoLin, HungyenZumbühl, AndreasLin, JerryZuñiga, Jose Maria Sebastian YLin, Jing (Janet)Zuo, LeiLin, Kuo-YiZupan, EvaLin, LinhanZurita, GroverLin, MaohuaZweiri, YahyaLin, Meng FangZych, MarcinLin, Ming-BoZymbler, Mikhail

